# Estimation of minimal data sets sizes for machine learning predictions in digital mental health interventions

**DOI:** 10.1038/s41746-024-01360-w

**Published:** 2024-12-18

**Authors:** Kirsten Zantvoort, Barbara Nacke, Dennis Görlich, Silvan Hornstein, Corinna Jacobi, Burkhardt Funk

**Affiliations:** 1 Institute of Information Systems, Leuphana University, Lüneburg, Germany; 2 Department of Clinical Psychology and Psychotherapy, Faculty of Psychology, Technische Universität Dresden, Dresden, Germany; 3 Institute of Biostatistics and Clinical Research, University Münster, Münster, Germany; 4 Department of Psychology, Humboldt-Universität zu Berlin, Berlin, Germany

**Keywords:** Psychiatric disorders, Therapeutics, Outcomes research

## Abstract

Artificial intelligence promises to revolutionize mental health care, but small dataset sizes and lack of robust methods raise concerns about result generalizability. To provide insights on minimal necessary data set sizes, we explore domain-specific learning curves for digital intervention dropout predictions based on 3654 users from a single study (ISRCTN13716228, 26/02/2016). Prediction performance is analyzed based on dataset size (*N* = 100–3654), feature groups (F = 2–129), and algorithm choice (from Naive Bayes to Neural Networks). The results substantiate the concern that small datasets (*N* ≤ 300) overestimate predictive power. For uninformative feature groups, in-sample prediction performance was negatively correlated with dataset size. Sophisticated models overfitted in small datasets but maximized holdout test results in larger datasets. While *N* = 500 mitigated overfitting, performance did not converge until *N* = 750–1500. Consequently, we propose minimum dataset sizes of *N* = 500–1000. As such, this study offers an empirical reference for researchers designing or interpreting AI studies on Digital Mental Health Intervention data.

## Introduction

The rapid advancement of artificial intelligence (AI) in various industries has spurred great anticipation for its transformative power in health care^1,2^. One area that particularly stands to benefit from AI-based improvements is mental health^3,4^. With 16% of global disability-adjusted life years attributed to them and staggering economic costs, mental disorders are immensely burdensome for individuals and societies alike^5^. Further, mental disorders are heterogeneous in their treatment needs, and AI promises a resource-efficient way to personalize, scale and improve mental health care^4,6–8^. However, among the central challenges in realizing AI’s envisioned potential within mental health interventions (MHIs) is the limitation of data set sizes^4,6,8–10^.

In contrast to diagnostics or public health data^3^, median data set sizes of machine learning (ML) application studies with MHI data barely exceed 100–150 patients^4,8,9,11^. Digital mental health interventions (DMHIs) allow for an easier collection of datasets than face-to-face (f2f) therapy^7,12^, but median data set sizes are still only 155–350^7,13,14^. This is problematic because prediction power is notoriously known to be overestimated in such small data set sizes^15–17^.

Sajjadian et al.^9^ found that MHI studies with small data set sizes reported significantly higher performance metrics than methodologically sound studies (*p* = 0.005). Further, they reported that 71% of the 59 investigated studies lacked an appropriate validation method and instead reported single test set or cross-validation (CV) results. Zantvoort et al.^13^ reported that DMHI dropout prediction models trained on small data sets produced the highest CV results but performed worst on the larger test set. As a result, several authors caution the interpretation of the current state of results and warn about possible consequences. Deploying an ungeneralizable model risks suboptimal care, deteriorating patient outcomes, wasted resources, and, thus, ultimately leads to the opposite of the intended effects^6,9,13,18,19^.

Despite their undebatable relevance, minimal necessary sample sizes, as they are standard in classical statistical settings, are uncommon in ML applications^20^. While no all-encompassing solution is available, a key approach for better understanding them are learning curves^20–22^. A recent study by Giesemann et al.^21^ produced such learning curves for dropout predictions in f2f psychotherapy and suggested 300 data points as a minimal necessary sample size. However, they only used eight patient-reported features and did not investigate overfitting or result variance. Further, only minimal insights are available into the interaction effect of sample sizes, model types and the number and type of features in DMHI data. Flexible models approximate realities’ complexity well, however, they risk overfitting, especially on small data sets^10,13,23^. Simple models tend to produce more stable results but risk disregarding valuable information^24–26^.

Additionally, the effectiveness of any model significantly depends on the nature and number of predictors^22,24^. Especially for DMHIs, feature numbers can quickly grow into hundreds of variables^12,27^. At the same time, data protection and adherence concerns call for a data minimalism approach^12,28,29^. Moreover, several papers have reported that fewer hand-crafted variables improved their results^12,30,31^.

In conclusion, the key questions repeatedly arising in ML studies in DMHIs are (1) how the dataset size influences the results^9,12,21^, (2) which of the ample algorithms to implement^21,26–28,30,32^, and (3) which of the abundant possible variables to use^12,27,30^. The current study aims to investigate the interdependence of these questions by analyzing the learning curves for dropout predictions across (1) six models with varying levels of flexibility and (2) six feature groups differing in their predictive power and extent. Beyond test set performance levels, the results will be investigated regarding their variance, generalizability from the training to test set, and convergence trajectory to derive insights into minimal necessary data set sizes. To this end, we leverage 3,654 users’ data from digital eating disorder prevention interventions provided to the general public in Germany^33^. Eating disorders are highly prevalent^34^ and associated with immense levels of suffering^35^. While DMHIs are effective in preventing and treating EDs, intervention dropout is a substantial issue among them^36^. Measures such as guidance can mitigate dropout but are costly^37,38^. Using AI to identify users at risk of dropping out allows for optimizing resource allocation and improving outcomes regardless of the availability of final symptom scores^30,37,38^. As such, within the limits of a single-dataset case study, this paper seeks to provide insights to improve the design and interpretation of ML studies on DMHI data.

## Results

### Final Values

The final data set comprised 3654 users, of whom 63% were classified as dropouts. Feature groups ranged from 2 features (F) (simple questionnaire), over 7 (simple behavior), 13 (selected behavior), 51 (extended questionnaire), and 64 (mixed) to a maximum of 129 features (extended behavior) in addition to the intervention information. The descriptive statistics, including for the training and test set, can be found in Supplementary Table 1.

Naïve Bayes (NB), Logistic Regression (LR), Support Vector Machines, (SVM) Random Forest (RF), adaBoost and shallow Multilayer Perceptron Neural Network (NN) models were trained with 10-fold CV on 80% of the assumed data set sizes between 100 and 3,654 users and evaluated on the test set of 731 users. Hyperparameters differed across settings (e.g., regularization for 7 vs 129 features), and are published in this study’s GitHub repository. All detailed result metrics are published in Supplementary Table 2. Supplementary Table 3 holds the p-values for the DeLong tests.

### Predictive Power of Feature Groups

**Fig. 1:**
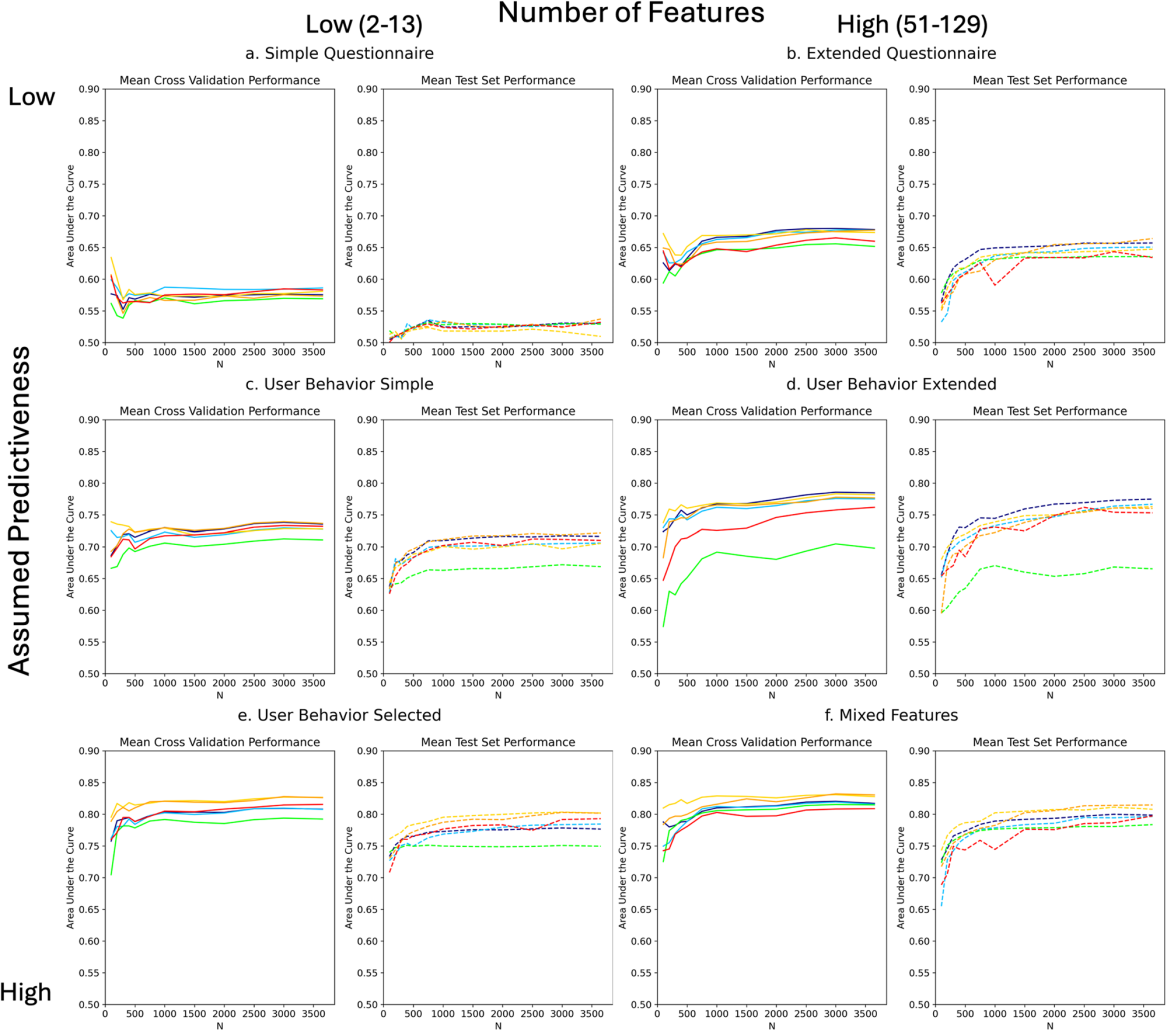
Training and test learning curves per feature type. Learning curves for (**a**) simple questionnaire, (**b**) extended questionnaire, (**c**) simple user behavior, (**d**) extended user behavior, (**e**) selected user behavior, and (**f**) mixed features. Each panels shows the respective mean AUC score for the Cross-Validation on the training data (solid line) on the respective left and mean test data performance (dotted line) on the right side. The colors of the lines represent the model types, i.e., Logistic Regression (dark blue), Support Vector Machines (light blue), Naïve Bayes (green), Random Forest (yellow), adaBoost (orange) and Neural Network (red).

Approximating the prediction performance in terms of the area under the curve score (AUC) via the best model on *N* = 3,654, the assumed predictive power across feature types was confirmed. As shown in Fig. 1a, b, there was no information in the simple (0.53 test AUC) and only moderate (0.66 AUC) in the extended questionnaire data. The simple behavior data (Fig. 1c) already achieved an AUC of 0.72, which was increased to 0.77 for the extended (Fig. 1d) and 0.80 for the selected (Fig. 1d) behavior data. From there, the mixed features (Fig. 1f) only slightly increased results to 0.81 AUC. Since the simple questionnaire data had no predictive power, its results will only be discussed in the context of overfitting.

### Overfitting on Small Data Set Sizes

Overfitting was a substantial problem for the small data set sizes (*N* ≤ 300), such that the CV results exceeded the test results by up to 0.12 in AUC (*on average* 0.05, see Supplementary Table 2). With increasing data set sizes (*N* ≥ 500), overfitting was substantially reduced for all features (mean 0.02, max. 0.06 AUC), except the simple questionnaire (mean 0.05, max. 0.07 AUC). Both the extent of overfitting on small data set sizes and its reduction with increased data set sizes varied across the (1) feature types and numbers and (2) model types.

Firstly, in terms of feature types, low-information feature groups (simple and extended questionnaire, Fig. 1a, b) were the most likely to overfit. For data set sizes of *N* ≤ 300, their avg. difference between the training and test scores without NB was −0.07 (max. −0.12 AUC). Choosing the winning model based on CV scores for the simple questionnaire data led to up to 70% of the results being >0.61 AUC despite a useless model (Table 1). Further, for these two feature types, up to *N* = 300 training results got worse with increasing data set size (avg. −0.03, max. −0.06 AUC) as seen in Fig. 1a, b. The same was visible in the simple behavioral data (Fig. 1c) but less severe and only for RF and SVM (avg. and max. −0.02 AUC for *N* ≤ 500).

**Table 1:** Overfitting as share of training Cross-Validation results (in %) that are at least +0.10 AUC higher than the respective test results per model and feature type

	LR	SVM	NB	RF	adaBoost	NN
***N*** = **100**						
Simple Questionnaire	0.40	0.40	0.30	0.70	0.60	0.40
Extended Questionnaire	0.50	0.60	0.10	0.60	0.50	0.50
Simple Behavior	0.40	0.40	0.30	0.50	0.40	0.20
Extended Behavior	0.30	0.30	0.00	0.50	0.50	0.10
Selected Behavior	0.20	0.10	0.00	0.10	0.30	0.10
Mixed Features	0.20	0.50	0.10	0.30	0.20	0.20
***N*** = **300**						
Simple Questionnaire	0.20	0.20	0.20	0.20	0.10	0.20
Extended Questionnaire	0.10	0.10	0.00	0.10	0.10	0.20
Simple Behavior	0.10	0.10	0.20	0.20	0.10	0.10
Extended Behavior	0.10	0.00	0.00	0.10	0.10	0.00
Selected Behavior	0.00	0.00	0.00	0.00	0.00	0.00
Mixed Features	0.00	0.00	0.00	0.00	0.00	0.00
***N*** = **500**						
Simple Questionnaire	0.10	0.10	0.10	0.10	0.10	0.10
Extended Questionnaire	0.00	0.00	0.00	0.00	0.00	0.00
Simple Behavior	0.00	0.00	0.00	0.00	0.00	0.00
Extended Behavior	0.00	0.00	0.10	0.00	0.00	0.00
Selected Behavior	0.00	0.00	0.00	0.00	0.00	0.00

For the extended behavior, selected behavior and mixed data, gaps between training and test set performance for N ≤ 300 were also prevalent but less severe (avg. −0.05, max. −0.09 AUC). For these three most informative feature groups, both training and test results increased with data set sizes (Fig. 1d–f), and the models winning in the training scores consistently also produced the highest test scores. Hence, the extent of overfitting in the results decreased as the information value of the features increased.

In terms of the number of features, the very small groups (simple questionnaire with 2, and simple behavior with 7 features) overfitted slightly more than their larger counterparts (14, 51 and 129 features). However, this effect was slightly reversed when increasing from selected behavior (13 features, mean 0.04 AUC, max. 0.06) to extended behavior (129 features, mean 0.05, max. 0.09) or mixed features (64 features, mean 0.05, max. 0.09).

Secondly, regarding model types, simpler models were less likely to overfit. As reported in Table 1, at *N* = 100, the share of CV results with at least +0.10 higher AUC than the test results was by far the lowest for NB (avg. 13%). On the other end of the spectrum, the tree-based models overestimated mode performance by at least +0.10 AUC in 42% (adaBoost) and 45% (RF) of the cases. However, across all models, these shares dropped substantially (avg. 7-8%, Table 1) for *N* = 300 and to mostly 0% by *N* = 500 (Table 1). Thus, the effect that more sophisticated models overfit more than simple models diminished with increasing data set size.

### Variance of Results

**Fig. 2:**
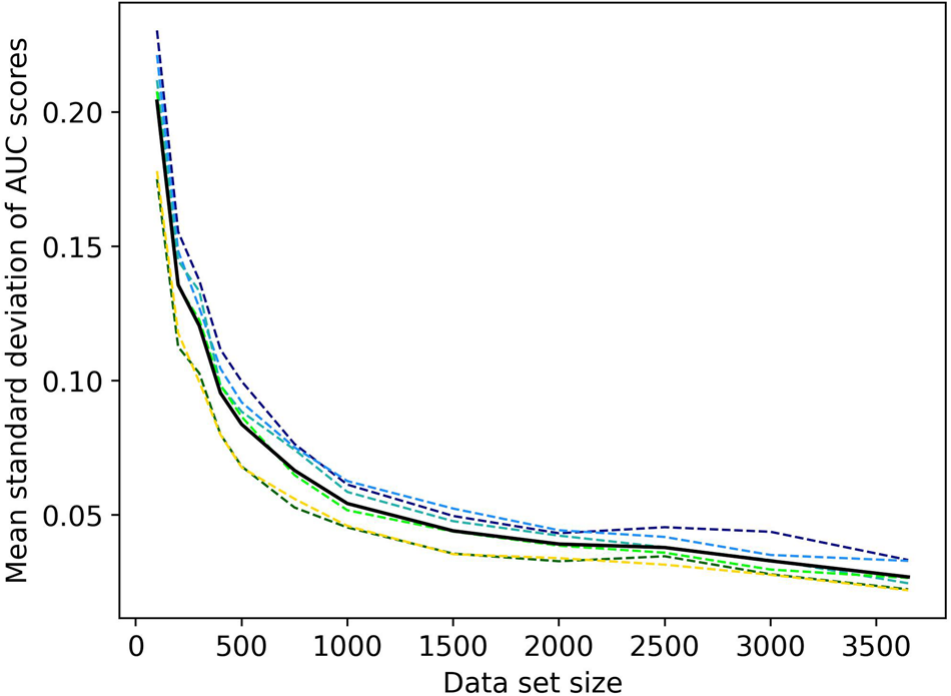
Cross-validation result variance per feature type. Mean standard deviation of the single folds’ area under the curve score as dotted lines in different colors per feature type i.e., simple baseline (dark blue), extended baseline (light blue), simple behavior (turquoise), extended behavior (light green), selected behavior (dark green), mixed features (yellow). Mean across all features in black solid line.

As shown in Fig. 2, the prediction results of the individual validation folds were highly unstable for small data set sizes. The AUCs’ standard deviation (S.D.) averaged across runs was by far the highest for *N* = 100 at 0.20 AUC. As such, the variability of AUC results spanned across a large part of the AUCs scale of 0-1, with the expectation to be between 0.5 (no information value) and 1 (perfect score). This variability steeply declined as the data set size increased as it had already halved by N = 400 (S.D. 0.10, Fig. 2). After that, it continued to drop, with the lowest average value in our results being S.D. 0.03 AUC at *N* = 3,654. As such, one can expect stable, thus similar, results for repeated calculations on large data, however, the results can largely differ when using small data sets.

Parallel to the observations in overfitting, the result variance was highest for the unpredictive feature groups. The single validation folds of *N* = 100 in the simple questionnaire data covered the entire AUC score range from very bad to very good (AUC mean 0.60, ±S.D. 0.37–0.83, min. 0.00, max. 1.00). Variance was lowest but still very high for the selected behavior data (AUC mean 0.70, ±S.D. 0.52–0.94, min. 0.10, max. 1.00).

### Performance Convergence per Model

**Fig. 3:**
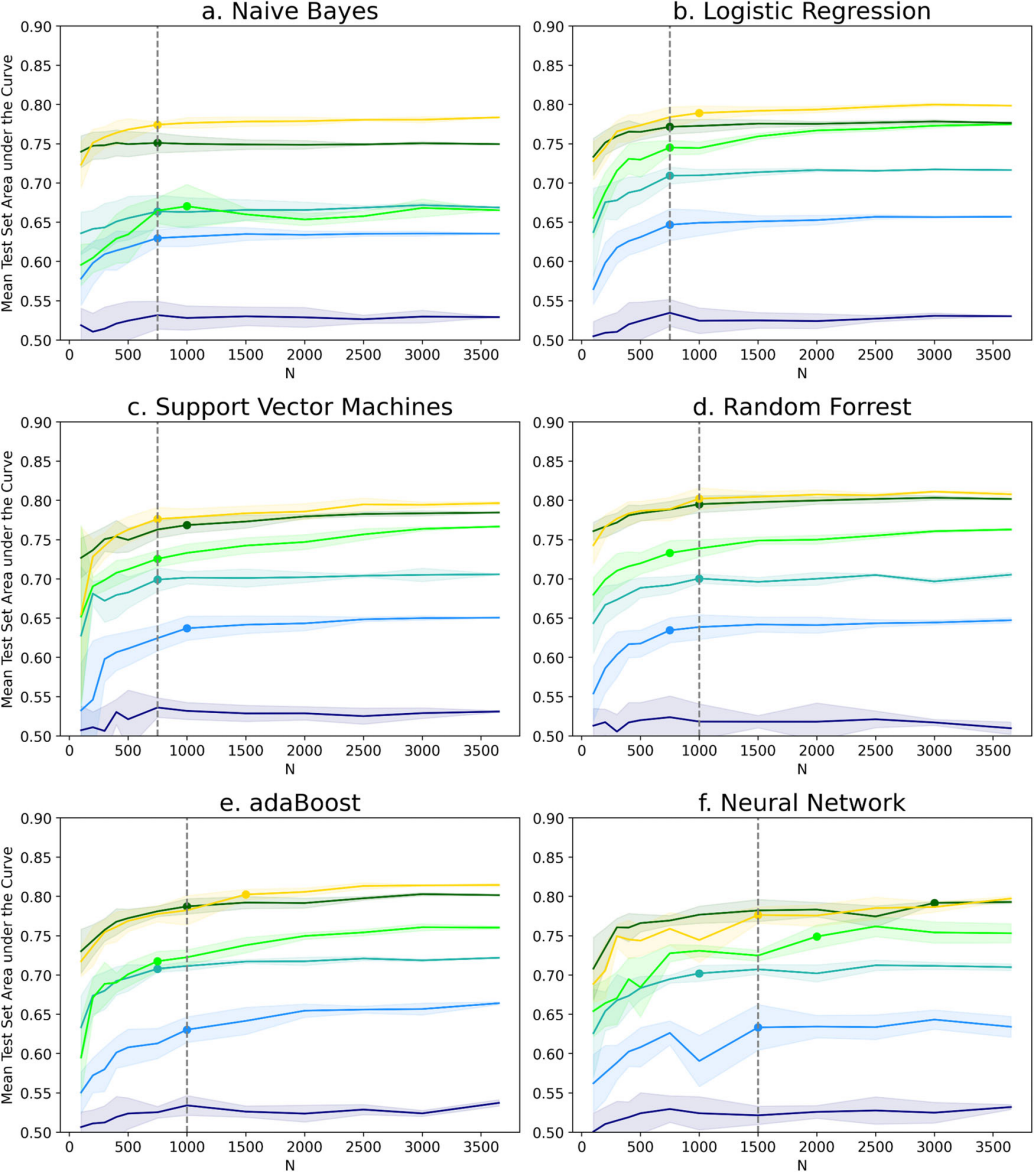
Test learning curve and convergence curves per model type. Learning curves on the test data per model: (**a**) Naive Bayes, (**b**) Logistic Regression, (**c**) Support Vector Machines, (**d**) Random Forest, (**e**) adaBoost, and (**f**) Multilayer Perceptron Neural Network. The colors indicate the different feature types, i.e., simple questionnaire (dark blue), extended questionnaire (light blue), simple behavior (turquoise), extended behavior (light green), selected behavior (dark green), mixed features (yellow). The respective mean area under the curve score is shown as sold horizontally plotted line and their S.D. as shaded area around it. Knee points indicate point of performance convergence as colored circles for the individual and grey dotted line as median across feature types. Knee points are not shown for simple questionnaire due to lack of predictive power.

The convergence points of the test set performance differed per model type and are shown in Fig. 3. The simple questionnaire results are shown in the graphs but ignored in the calculations as there was no predictive power to converge towards.

The simpler models NB, LR and SVMs (Fig. 3a–c) all had a median convergence point of *N* = 750. The more sophisticated tree-based models converged later at *N* = 1,000 (Fig. 3d, e), followed by the NN at *N* = 1500 (Fig. 3f). The NB (Fig. 3a) had no performance improvement (+0% AUC) when provided with large data set sizes (*N* = 3,654 instead of 750), whereas LR (Fig. 3b) and RF (Fig. 3d), on average, grew +2%. SVMs (Fig. 3c) and NNs (Fig. 3f) could slightly better leverage the largest data set (+3%) but were surpassed by adaBoost (Fig. 3e) on average increasing the AUC between *N* = 750 and 3,654 by +5%.

It is noticeable that the NN (Fig. 3f) showed oscillation and larger variability in the results for much longer than the other models, where this only occurred for very small data sets. Training it on the small data sets partly ( < 20% of runs) gave convergence warnings.

### Marginal Value and Convergence of Additional Features

The marginal benefit of complex features was highest for large data set sizes, and more predictive feature groups tended to converge at higher data set sizes (Fig. 3).

**Fig. 4:**
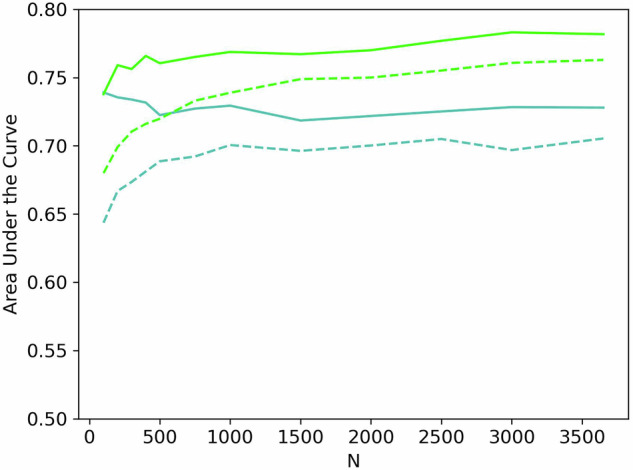
Random Forest simple versus extended behavior features. Random Forest area under the curve for Cross-Validation on training data (solid lines) and test data (dotted lines) learning curve for simple behavior data (F = 7) in green and extended behavior data (F = 129) in turquoise.

Adding the extended questionnaire features to the simple ones (F = 51, dark and light blue lines in Fig. 3) continuously improved results as the data set size grew (avg. 0.51–0.53 versus 0.55–0.66 test AUC for *N* = 100–3,654). The same was the case for increasing the simple to the extended behavior data (avg. 0.63–0.70 versus 0.64–0.75 test AUC, turquoise and light green lines in Fig. 3). Due to overfitting on *N* = 100, the best CV results for the simple behavior were equal to those of the extended behavior data (AUC = 0.74), despite being lower on the test set (0.64 vs 0.68 AUC), as shown in Fig. 4. This effect faded with increasing data set size, and at N = 500, even the test set performance of the extended group surpassed the simple one’s CV scores.

Similarly, using selected instead of extended behavioral data was most beneficial on the small data sets (+0.08–0.03 test AUC difference at N = 100–3,654, Fig. 1e, d). Generally, for all models but the NB, the extended behavior data curve (light green in Fig. 3) was the steepest after *N* = 1000, such that it was closing the gap to the selected behavior features. For LR (Fig. 3b), it even had already matched the selected behavioral data’s performance at *N* = 3654.

Adding more than 50 questionnaire features to the selected behavior data for the mixed data set (yellow in Fig. 3) first led to slightly less (*N* ≤ 200, avg. difference in test AUC −0.02), then equal (*N* = 300–500, 0.00), and ultimately slightly better performance (*N* > 500, +0.01). As the only exception, using selected (F = 13) instead of simple (F = 7) behavioral data was always beneficial, but most so on the small data sets (avg. +0.12–0.08 test AUC difference for *N* = 100–3,654).

### Model and Feature Combinations

Naive Bayes (NB, green in Fig. 1) obtained competitive test results (top3 models) for smaller data set sizes, specifically for the extended questionnaire (*N* ≤ 750), mixed features (*N* ≤ 400), selected behavior (*N* ≤ 200), and simple behavior (*N* = 100). However, NB never outperformed the respective other top3 models (*p* > 0.05). Furthermore, as shown in Fig. 1c–e, NB significantly underperformed compared to the other models for behavior data, particularly for extended features and larger data set sizes (*p* < 0.05).

Logistic Regression (LR, dark blue in Fig. 1), on the other hand, performed very well in almost all settings. It consistently outperformed most models for the extended questionnaire data for *N* = 200–500 (*p* < 0.05). For *N* > 500, LR continued performing well but was first matched by RF and later (*N* > 2500) by adaBoost. In the extended behavior data, LR was below or equal to RF for *N* ≤ 200 but significantly outperformed all models (*p* < 0.05) with few exceptions after that.

Support Vector Machines (SVMs, light blue in Fig. 1) mainly performed in the mid-field but were most competitive with a linear kernel in the two extended feature types. As such, they performed similarly to the top model LR on extended behavior data for *N* > 2500 (*p* = 0.06–0.08) and regularly outperformed (*p* < 0.05) NB, NN and adaBoost.

Similarly to LR, Random Forest models (RFs, yellow in Fig. 1) performed very well, especially for the highest information feature types. They consistently outperformed all models for selected behavior and mixed features, with the only regular exception being adaBoost for *N* > 750 in selected behavior and *N* > 1000 in mixed features.

adaBoost (orange in Fig. 1) tended to perform better with larger data set sizes. For the highest information features, it progressively caught up to RF as of *N* > 400. Additionally, adaBoost performed very well in the simple behavior data (*N* > 100) and the extended questionnaire data (*N* > 1500).

Multilayer Perceptron Neural Networks (NN, red in Fig. 1) were among the top3 models for simple behavior (*N* > 750) and selected behavior (*N* > 200) data and occasionally performed well for extended behavior data. NN’s most competitive results were for data set sizes of 1500 or more, where it was most likely to outperform NB, LR, or SVMs.

### Recall-Precision Tradeoff

While the detailed results are discussed on the AUC only for clarity and brevity, the following reports the noteworthy tendencies for recall and precision at the default threshold of 0.5. All detailed metrics, including balanced accuracy, f1-score, precision, and recall, are published in Supplementary Table 2.

adaBoost generally achieved among the highest recall scores across runs, but in the case of the respective simple and extended versions of the questionnaire and behavior data, it was at the expense of precision. A similar pattern was observed for the NB model, which either had high recall or high precision but never a winning balance. For selected behavior and mixed features, the NN and adaBoost models achieved the most balanced result between recall and precision. However, as reported above, they were outperformed by the RF model in terms of AUC, which—at the default threshold—achieved higher precision than recall.

## Discussion

Sophisticated ML models promise to disrupt mental healthcare through resource optimization and personalization^7,9^, for example by lowering dropout^38^ and improving health outcomes^39^. However, in DMHI settings, median data set sizes barely reach 155–350^7,9,27^. Such data set sizes are known to overfit and have been proven to not suffice for many sophisticated models^15–17^. However, very limited insights are available as of which data set size these problems are mitigated in DMHI settings. Therefore, the current study leveraged a dataset 10–24-times as big as the reported medians to evaluate performance levels, internal generalizability and variance across different feature groups (i.e., low to high predictive power with F = 2–129) and six model types (Naïve Bayes, Logistic Regression, Support Vector Machines, Random Forest, adaBoost, and Multilayer Perceptron Neural Network models).

Our first key finding confirms that CV results on small, thus most common, data set sizes overestimate the prediction performance. Especially worrisome is that the effect was exacerbated for uninformative features, such that a useless model had up to a 70% likelihood of returning seemingly good CV scores. Further, we reproduced the negative correlation between data set sizes and CV results^9,13^ for *N* ≤ 300 and partly *N* ≤ 500 for the least predictive features. In these settings, such high training results were associated with the worst test results^13,18^. While overfitting was also prevalent in *N* ≤ 300 for the more predictive features, it was lower, and the best training translated to the best test results. Further, among all features, the individual validation scores were highly unstable for *N* ≤ 300 (S.D. 0.13–0.20 AUC). Evaluating on a single fold is common^9,18^, and publication bias risks an overrepresentation of the higher end of that variance in published studies^8,40^. Thus, we conclude that results from data set sizes of *N* ≤ 300 imply a substantial risk of being inflationary and ungeneralizable, especially for features with low predictive power.

A second, closely related key result is that CV scores on small data sets risk underestimating the superiority of complex versus simple features. This is caused by, firstly, large data being necessary to leverage additional features and, secondly, simple features overfitting more. For the largest feature group (F = 129), our data set size even may have been too small as it continued catching up to the already converged selected feature’s performance. However, more research on larger data sets is necessary to investigate this hypothesis. Therefore, we tentatively confirm previous findings^12,31^ that hand-crafted and theoretically driven selected features are preferable, especially for small data sets.

The third key result confirms that simpler models are less likely to overfit but converge earlier and are less competitive for higher data set sizes. More flexible models, on the other hand, heavily overfit small data sets but produce the best results on the high information features, especially for large data set sizes. Consistent with theory and empirical evidence^25,41^, particularly NB gave robust results but was not very competitive overall. On the other end of the spectrum, especially RF and SVMs seemed very competitive on noisy and small data sets but actually overfitted^25,42,43^. adaBoost performed badly on small but was most effective in leveraging large data sets. RF was one of the two most competitive algorithms across settings, already efficiently leveraging mid-sized data sets for predictive features^25^. LR was the second competitive algorithm, confirming its balance of overfitting less on small data sets^43^ but only partly being outperformed in large data sets. The fact that LR is easier to interpret and faster to train than the tree-based models emphasizes its essential role as a staple baseline model to beat^6,27,31^.

The fourth key finding is that prediction performance in our study did not converge until *N* = 750 for simpler, and 1000–1500 for more sophisticated models. Both are substantially above Giesemann et al.’s^21^ findings that their results stopped improving at *N* = 300. A possible explanation is that their study on f2f-therapy investigated only eight features, which all fall in our extended baseline definition. As a result, their maximum test AUC score (*N* = 10,000) was 0.62, which our extended baseline data also achieved at *N* = 300. Further, in our data, more predictive features partly converged later than those with less information value. One possible hypothesis could, therefore, be that their earlier convergence point may be due to the limit of available predictive information in the features used. Thus, we conclude that more sophisticated models paired with larger data set sizes (*N* > 750) are necessary to approximate the true potential for the common feature groups in DMHIs.

Beyond the potentially still too-small sample size of 3654, this paper has several limitations. Firstly, it is only one case study, and while concurring with previous knowledge, this study per design does not suffice to reliably differentiate between setting-specific and generalizable tendencies. Further, the study at hand only focuses on internal generalizability and does not evaluate the models on an external data set. As models already overfitting the internal validation are unlikely to generalize to new data sets, our study constitutes a first step in the improvement of generalizability in this research area^18^. Regarding sample bias, the interventions considered are preventative and the sample only comprises self-referred female participants. Additionally, the five study arms were heterogeneous in their content, lengths, and user symptom strength^33^. As pooling interventions already mitigates overfitting^13^, results may differ if repeated on a single intervention. However, this also implies that overfitting in this study may be underestimated, making the proposed increase of minimal data set sizes even more critical. Hence, the current study presents first insights, but more research is necessary to confirm the proposed minimal data set sizes. As a second limitation, while the operationalization of the outcome and feature groups was empirically and theoretically founded, many other options^12,27,44^ are possible and may influence results. We proposed six different feature groups representing low to high predictiveness for intervention dropout, but they would, for example, differ in health outcome predictions^27,45^. Further, although recent works substantiate the assumption that our findings still apply^9,11,19^, features such as neuroimaging or biological data are not considered in the current study. The same limitation applies to pre-processing steps and model choice, including more sophisticated Neural Networks than the shallow MLP used. Fourthly, while using the elbow method allows an analytical approach to determining convergence, it does not consider the trade-off of the cost that additional data points induce. Further, oscillations can influence elbow points, though mitigated by choosing the global instead of local elbow point.

In terms of recommendations, we, firstly, strongly discourage mistaking CV or, even less so, single test set results for suitable performance measures on small data set sizes (*N* = 100–300). Doing so exacerbates publication bias and causes ungeneralizable result expectations^13,18,19,40^. A key step against overfitting is separating the validation set for the hyperparameter decision from the model choice, for example, through nested CV^15^. Ideally, models should be validated on external data sets in addition to the internal validation methods in order to ensure broader generalizability^18^. Further, especially for complex features or ones with unknown or low information value, having a reasonably sized test set is indispensable^18,46^. Based on our results and previous suggestions^46^, we, therefore, propose a minimal data set size of *N* = 500 for predictions in DMHIs to mitigate overfitting.

Secondly, even though *N* = 500 started producing internally reliable results, it did not suffice to approximate many of our feature groups maximum predictive power. Performance did not converge until *N* = 750 for LR, SVM and NB, and for the more flexible models, it even required *N* = 1000–1500. Further, the predictive power of additional and mixed features increased in higher data set sizes. We, therefore, suggest *N* = 1000 as a minimal data set size when comparing simple to more complex feature groups.

Lastly, and closely related to the other points, we recommend being mindful of the interaction between the nature and number of features, data set sizes and models. While ML methods can theoretically handle many features, for small data set sizes, the noise of additional features and the models’ ability to overfit it must be considered^24,25,41,43^. Further, the hyperparameters, especially those concerning regularization, need to be chosen accordingly. To determine the adequateness of the set-up, we suggest implementing and reporting a learning curve approach leading up to the maximum available data set size. On the one hand, a downward CV trajectory suggests substantial overfitting. On the other hand, a continuously steep upward trajectory of both CV and test results suggests an underestimation of the predictive power due to a lack of data.

In summary, this paper contributes to the field of research by providing insights to aid the design and interpretation of predictions in DMHI settings. As such, it aims to combat unrealistic result expectations and the consequent disenchantment in a field where AI can be of great value but is only gradually gaining a foothold.

## Methods

### Case Study Background—everyBody Study

The everyBody dissemination study (ISRCTN13716228) provided evidence-based eating disorder (ED) prevention and health promotion programs^47–50^ in Germany^33^. Participants (*N* = 3654) were adult women without full-syndrome EDs recruited from the general population between November 2016 and May 2019. All participants gave informed consent to participate in the study, and participation was anonymous. This primary study was a stratified, nonrandomized, parallel-group interventional design where intervention content matched risk and symptom levels. From the total sample, 452 users were allocated to the Basic intervention, 397 to Original, 1386 to Plus, 80 to AN, and 1339 to Fit. The interventions comprised 4 to 12 weekly online sessions (20 to 60 min) based on cognitive-behavioral principles, including psychoeducation, exercises to promote body image and balanced eating, and—if applicable—to reduce ED symptoms. Four out of five interventions were supplemented with daily or weekly online diaries. Four interventions had access to moderated peer group discussions, and two included weekly coach feedback messages.

Questionnaires were completed at screening, baseline, mid-intervention, post-intervention, 6-month, and 12-month follow-up. Analysis of pre-post changes of weight-related concerns within the completer subset revealed notable decreases in weight-related concerns across four of the five study arms (effect sizes d = −0.45 to d = −0.94)^51^.

The screening and allocation process, individual intervention design and data generation is described in detail in Supplementary Note 1. Additional information can be found in the pre-registration protocol of the study^33^ and its primary publication^51^. The trial was approved by the ethics board of Technische Univeristät Dresden (EK 83032016) and pre-registered at ISRCTN (No. 13716228, 26/02/2016). All participants gave informed consent to participate in the study, and participation was anonymous.

### Definition of Outcome

**Fig. 5:**
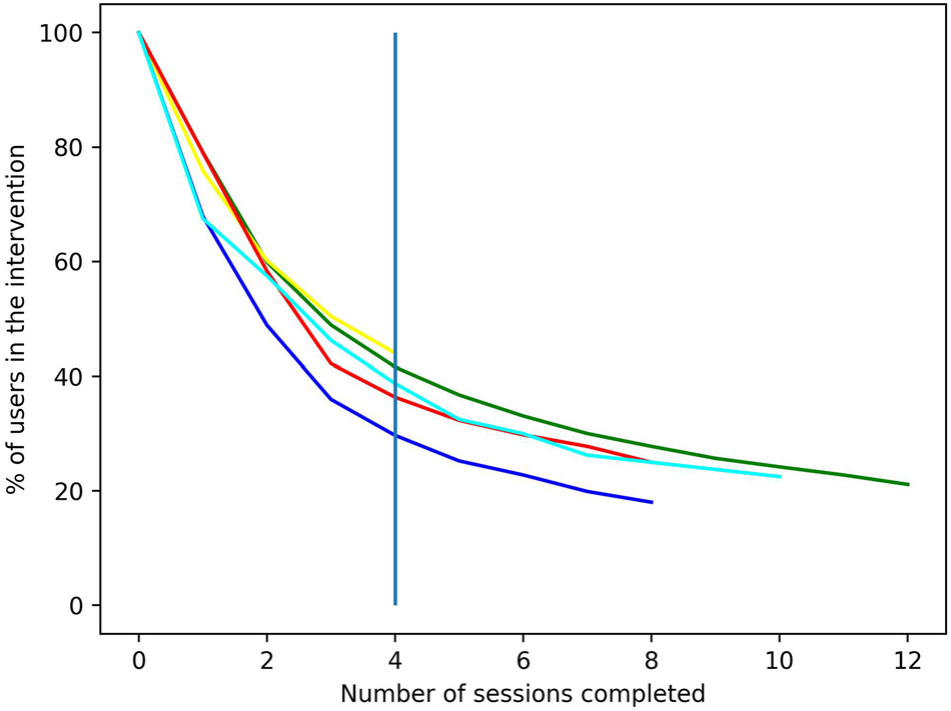
Dropout curves per intervention arm. Dropout curves defined by the share of users that finished each number of sessions across interventions, i.e., Fit (green), basic (yellow), Plus (dark blue), original (red), AN (turquoise). Vertical blue line indicates the cutoff point of four models, such that patients leaving on the left are categorize as dropouts and those on the right as completers.

Session completion was chosen to operationalize dropout, as it was found to be the most closely connected to intervention outcome^52^. While the different interventions had variable numbers of sessions (4–12), they presented similar dropout patterns, as seen in Fig. 5.

Therefore, completing less than four sessions was defined as dropout to account for the minimum length of four weeks in the shortest intervention. This definition led to 56% dropout in the Basic intervention, 64% in Original, 70% in Plus, 61% in AN, and 58% in Fit. While many other dropout definitions are possible^53^, this operationalization presents the possibility of identifying the users most at risk of leaving across interventions while ensuring sufficient time left to intervene^39^.

### Feature Groups and Pre-Processing

The most common overarching categories for dropout predictors are questionnaire data and intervention user behavior data^12,27^. For the current study, feature groups were categorized based on the number of features and their empirically proposed predictive power regarding dropout. The categories considered and their key details are shown in the overview in Table 2 and briefly described in the text below. Across all feature groups, the basic information of which intervention the user participated in, its lengths in weeks, and the starting year was also added.

**Table 2:** Overview Feature Groups

Name	Description	Key Aspects	#
Simple Questionnaire	Primary symptom scores (WCS)^58^ at screening and baseline	Assumed low predictive power^27,30,32,57^, available before intervention start	2
Extended Questionnaire	Variety of self-report questionnaires incl. WCS^58^ and further eating disorder^67,68^, depression^69^, and anxiety^70^ symptoms and behavior patterns, personality^71^, self-regulation^72^ and self-esteem scores^73^, psychiatric and weight loss history, alcohol use^74^, socio-demographic information, and user expectations.	Assumed low predictive power^27,30,32,57^, theoretically available before intervention start but with high time-invest from users	51
Simple User Online Behavior	Sum of logins per day of the first week	Assumed high predictive power^28,30^, very simple to obtain	7
Selected User Online Behavior	Single aggregation for the first week of time to complete sessions, seconds spent, number of logins, number and length of answers, diary entries and messages to coaches and group chat	Assumed high predictive power^12,13,27,31^ with effort into researching and choosing most promising options and aggregation measures	13
Extended User Online Behavior	Variables from log files aggregated per day of the first week, incl. sessions completed, seconds spent, log ins, time spent in beginning/mid/end of the week and morning/day/evening, session completion, count and number of characters of diaries, group, and coach messages, exercises, answers to the sixteen most common closed questions as mean, min and max	High predictive power but possible loss due to complexity^12,31,38^, automatically collected during first week of intervention with limited time invest	129
Mixed Features	Extended questionnaire + selected user online behavior	Mixed, with reported increase of predictive value^27^	64

The translated original questions and units can be found in Supplementary Table 1. An overview of the almost 200 features’ description including their number of missing values is provided in Supplementary Note 2. All data processing was done in Python, primarily relying on the NumPy^54^ and Pandas^55^ libraries. Missing values were imputed with a multivariate iterative imputer^56^ using the training sets questionnaire and weekly aggregated user behavior variables described below.

Regarding the questionnaire data, for the primary dissemination trial, various items were collected before intervention start, ranging from the standard primary symptom data up to less common measures such as personality scores. As pre-intervention questionnaire data has limited predictive power regarding dropout by itself^27,30,32,57^, it was used to investigate a low predictive power setting. For the simple questionnaire data, only the screening and baseline primary symptom questionnaires (Weight Concern Scale^58^) were used. For the extended questionnaire data another 49 measures on psychological symptoms and characteristics, socio-demography, and user expectations were chosen based on their availability from the primary study and assumed usefulness. As a result, missing data was minimal, with five variables with <1.5% and six variables with <15% missing entries. The six latter were voluntary, and most users either answered all or none. Therefore, an additional variable was added to indicate this choice.

For the intervention user behavior data, log files and user submissions were aggregated into a set of simple, selected, and extended features. Only data from the first week of the intervention was used to leave sufficient time to intervene against dropout.

The simple behavior data followed related work on generalizable features in DMHIs and counted the users’ number of logins per day for the first week of the intervention^28,30^. For the selected user behavior, features were selected based on the related work^12,13,27,31,45^ and aggregated per week, mitigating sparsity, multicollinearity, and complexity. For the extended user behavior, the same raw data instead was separately aggregated per day and included additional less known or theoretically less informative features as well as more aggregation forms (e.g., mean, minimum and maximum).

For the mixed features, the two types of features (selected behavior and extended questionnaire data) were added together for the last group to consider possible interaction effects^27^.

### Algorithms

Six common ML algorithms^16,26^ were included in a trade-off of investigating different models while maintaining a reasonable computational load and ability to present results. For the simple algorithms, Naïve Bayes (NB)^59^, Logistic Regression (LR), and Support Vector Machines (SVMs)^60^ with a linear and radial kernel option and classifier were trained. In terms of more sophisticated tree-based models, first, Random Forest (RF) models were used due to their high flexibility and good performance in similar settings^13,26,27^. Second, to leverage the upsides of sequentially combining several tree learners, adaBoost decision trees were included. Lastly, a Multilayer Perceptron covered the family of Neural Networks (NNs). Considering the simplicity and small data set sizes at hand, a shallow architecture with a single hidden layer was chosen. All of these model types have been extensively discussed in various sources^16,61^ and will, therefore, not be further detailed here.

### Learning Curves and Training Set up

To estimate training performance, 10-fold cross-validation (CV) with grid search was implemented. The best resulting estimator was re-trained on the entire training dataset and evaluated on the previously set aside test set of 20% of the data. A standard scaler was incorporated into the pipeline. Regarding the hyperparameter ranges, default values were expanded upon if the outermost values appeared insufficient or excessive within the training data results.

Following authors such as Giesemann et al.^21^, Balki et al.^20^, and Perlich et al.^23^, learning curves were used to provide insights into the effect of sample size on prediction performance. For the data set sizes, the space of 100, 200, 300, 400, 500, 750, 1000, 1500, 2000, 2500, 3000, and 3654 was explored to balance a comprehensive investigation with computational costs. The models were trained on 80% of the respective N to represent the data set sizes. The test set was stratified for dropout and each of the samples was stratified across the fives interventions. Further, training was repeated on samples drawn with different seeds ten times for small data set sizes ( ≤ 500), five times for the mid data set sizes ( ≤ 2000), and three times for the remaining large dataset sizes^21^. The model training was implemented with the scikit-learn^62^ library in Python, and the code is publicly available in this paper’s GitHub repository.

### Evaluation and Result Analysis

The area under the curve (AUC) score was used to compare results across all settings without depending on a threshold. In terms of evaluation, the scores were classified into no (0.50–0–0.56 AUCs), low (0.57–0.64), moderate (0.65–70), good (0.71–0.75) and very good (>0.75) predictive power^63^. Predictive power per feature group was approximated through the test score for the model type with the highest training scores at *N* = 3654. A two-tailed DeLong test^64,65^ with a significance threshold of α = 0.05 was used to compare the test AUCs between models. The DeLong test was chosen because it is non-parametric, aimed at comparing AUCs and sufficiently computationally efficient^64^. The test returns the *p*-value for the null hypothesis of equal performance, hence the assumption that no model performs better than the other. Failing to reject the null hypothesis (*p* > 0.05) leads to possible differences in the AUC being assumed to be due to random chance.

The variability of results was determined through the standard deviation of single validation results across repetitions. To determine overfitting, first, the difference between the mean training and test score was considered. Next, the percentage of CV scores at least +0.10 AUC higher than the mean test set were investigated. The threshold 0.10 was chosen as it is a step that definitively jumped one results categorization introduced above, meaning, for example, a “low” score would become “good”. Performance convergence was investigated by considering the diminishing marginal benefit of adding more data through the so-called elbow method. To this end, the kneed algorithm^66^ Python implementation was used and set to find the global convergence point.

## Supplementary information

Supplementary Material

## Data Availability

The data used in this study is not publicly available due to legal restriction caused by the limitations in the data usage agreements and participants consent. However, qualified researchers can apply for data access through contacting the authors of this paper. The primary study’s pre-registration is published^33^.

## Code availability

The code for the learning curves can be accessed through the following GitHub repository without restrictions: https://github.com/KiraZant/everybodylearningcurves.
