# Supplementary material for: Estimation of minimal data sets sizes for machine learning predictions in digital mental health interventions
Source: NPJ Digit Med. 2024 Dec 18;7:361. doi: 10.1038/s41746-024-01360-w (PMC11655521; doi:10.1038/s41746-024-01360-w)
Supplement: Supplementary file 1 — Supplementary Material [file 41746_2024_1360_MOESM1_ESM.pdf]

Supplementary Table 1 – Descriptive statistics of total sample and train-test split with feature descriptions

Category	Variables	Total Sample				Training	Test	Units	Original Question/ Categories Used
		Mean	Std	Min	Max	Mean	Mean		
**Basic Data**	Year of intervention start	2018	1	2016	2019	2018	2018	Year	
	Intervention duration	9	3	4	12	9	9	Weeks	
	AN	0.02		0.00	1.00	0.02	0.02	Binary	
	Fit	0.37		0.00	1.00	0.37	0.35	Binary	
	Plus	0.38		0.00	1.00	0.38	0.39	Binary	
	Basic	0.12		0.00	1.00	0.12	0.13	Binary	
	Original	0.11		0.00	1.00	0.11	0.11	Binary	
**Dropout Variable**	Dropout before finishing 4 modules	0.63		0.00	1.00	0.63	0.63	Binary	
**Simple and Extended Questionnaire**	Weigth Concern Scale at screening	51.67	17.62	0.00	100.00	51.76	51.31	Continous, see question	Weight Concern Scale
	Weigth Concern Scale at pre-intervention	51.31	17.92	0.00	100.00	51.35	51.12	Continous, see question	Weight Concern Scale
**Extended Questionnaire**	Age	40.97	13.73	18.00	84.00	41.02	40.77	Years	
	Occupational status 5	0.30		0.00	1.00	0.30	0.30	Binary	What is your current employment status? (multiple answers possible)<br>1 in training<br>2 job-seeking<br>3 homemaker<br>4 self-employed<br>5 part-time employment (less than 35 hours)<br>6 full-time employment (35 or more hours)<br>7 retired
	Occupational status 1	0.19		0.00	1.00	0.19	0.21	Binary	
	Occupational status 4	0.07		0.00	1.00	0.07	0.06	Binary	
	Occupational status 3	0.07		0.00	1.00	0.07	0.07	Binary	
	Occupational status 6	0.38		0.00	1.00	0.38	0.35	Binary	
	Occupational status 7	0.07		0.00	1.00	0.07	0.06	Binary	
	Occupational status 2	0.04		0.00	1.00	0.04	0.05	Binary	
	Highest completed education	3.34		0.00	5.00	3.31	3.43	Ordinal, see question	What is your highest educational qualification? (For foreign qualifications, please state the German equivalent)<br>0 Vocational training preparation, vocational preparation year ("Berufsvorbereitungsjahr"), other qualification, in training without previous qualification<br>1 Secondary school certificate, entry qualification (“Einstiegsqualifizierung”), vocational school (basic vocational training)<br>2 Secondary school leaving certificate, 2-year dual vocational education and training<br>3 Abitur, 3- or 3½-year dual vocational training program<br>4 Bachelor's degree, certified business administrator (“Fachkaufmann”, “Fachwirt”), master craftsman<br>5 Diploma (university or university of applied sciences), master's degree, doctorate
	Inhabitants of current living location	4.43		1.00	7.00	4.41	4.48	Ordinal, see question	What is the population of your place of residence?<br>1 less than 5,000<br>2 5.000-10.000<br>3 10.000-20.000<br>4 20.000-50.000<br>5 50.000-100.000<br>6 100.000-500.000<br>7 more than 500,000
	Single	0.24		0.00	1.00	0.24	0.23	Binary	What is your marital status?<br>1 Single, Divorced without a new relationship, Widowed without a new relationship<br>0 In a relationship, married, registered civil partnership or relationship with shared household, divorced with new relationship, widowed with new relationship
	Lifetime psychological disorder	0.31		0.00	1.00	0.30	0.31	Binary	Have you ever been diagnosed with a mental disorder?
	Prior psychotherapy	0.34		0.00	1.00	0.33	0.35	Binary	Have you ever received psychotherapeutic treatment for a mental disorder?
	Weightloss in past year	1.81		1.00	8.00	1.81	1.80	Ordinal, see question	What is the maximum weight you have lost in the past year?<br>1 0-2 kg<br>2 3-5 kg<br>3 6-10 kg<br>4 11- 15 kg<br>5 16-20 kg<br>6 21-25 kg<br>7 26-30 kg<br>8 more than 30 kg

Category	Variables	Total Sample Mean	Std	Min	Max	Training Mean	Test Mean	Units	Original Question/ Categories Used
**Extended Questionnaire**	Weight loss duration in regards to weightloss past y	5.02		1.00	14.00	5.01	5.07	Ordinal, see question	Over how many months did this weight loss occur?<br>1 I have not lost any weight.<br>2 <1 month<br>3 1 month<br>...<br>14 12 months
	Missing values for personality scores	0.14		0.00	1.00	0.14	0.13	Binary	1 if no answer was given to personality score questions
	BFI extraversion	6	2	2	10	6	6	Continous, see question	Big Five Inventory (BFI-10) extraversion
	BFI agreeableness	6	1	2	10	6	6	Continous, see question	Big Five Inventory (BFI-10) agreeableness
	BFI conscientiousness	7	2	2	10	7	7	Continous, see question	Big Five Inventory (BFI-10) conscientiousness
	BFI neuroticism	7	2	2	10	7	7	Continous, see question	Big Five Inventory (BFI-10) neuroticism
	BFI openness to experience	7	2	2	10	7	7	Continous, see question	Big Five Inventory (BFI-10) openness to experience
	Treatment expectation - Usefulness	6.33	1.81	1.00	9.00	6.31	6.41	Ordinal, see question	How useful does the program offered here seem to you at the current moment?<br>1 - not useful at all - 9 - very useful
	Treatment expectation - Probability of lowering sym	5.62	1.78	1.00	9.00	5.61	5.65	Ordinal, see question	How promising do you think the program is for reducing your symptoms?<br>1 - not at all - 9 - very promising
	Treatment expectation - Recommendation to friend	5.48	2.04	1.00	9.00	5.47	5.51	Ordinal, see question	How confident are you that you can recommend the program to a friend who has similar complaints?<br>1 - not confident at all - 9 - very confident
	Treatment expectation - Extened of symptom impro	49.33	22.39	0.00	100.00	49.32	49.36	Continous, see question	How much do you think your symptoms will have improved at the end of the program?<br>0 - 100%
	Treatment expectation - Treatment Helpfulness	5.29	1.96	1.00	9.00	5.29	5.26	Ordinal, see question	Based on your current feelings, how much will the program help to alleviate your symptoms?<br>1 - not at all - 9 - very much
	Treatment expectation - Feeling about symptom im	46.39	24.88	0.00	100.00	46.49	45.99	Continous, see question	Based on your current feelings, how much will your symptoms have improved at the end of the program?<br>0 - 100%
	BMI at screening	27.32	5.87	15.79	101.56	27.34	27.26	Continous, see question	Calculated Body Mass Index
	BMI at pre-intervention	27.29	5.69	15.79	67.95	27.32	27.16	Continous, see question	Calculated Body Mass Index
	Generalized Anxiety Disorder Scale-7 (GAD-7)	6.17	4.39	0.00	21.00	6.20	6.09	Continous, see question	Generalized Anxiety Disorder Scale-7 (GAD-7)
	Patient Health Questionnaire-9 (Depression)	7.66	4.90	0.00	27.00	7.68	7.60	Continous, see question	Patient Health Questionnaire-9 (Depressivität)
	Screening Loss of Control Symptoms	1.51	1.97	0.00	5.00	1.51	1.49	Continous, see question	On average, how often in the last 3 months have you had the feeling of losing control over your eating behavior?<br>0 - less than 1x per month<br>1 - once per month<br>2 - 2x per month<br>3 - 3x per month<br>4 - 4x per month<br>5 - more than 4x per month
	Alcohol Use Disorders Identification Score	2.59	1.92	0.00	12.00	2.58	2.60	Continous, see question	Alcohol Use Disorders Identification Test
	Rosenberg Self-Esteem Score	30.06	6.33	10.00	40.00	30.06	30.04	Continous, see question	Rosenberg Self-Esteem Scale
	Eating Disorder Examination-Questionnaire Score	2.50	1.13	0.00	5.95	2.51	2.49	Continous, see question	Eating Disorder Examination-Questionnaire (EDE-Q)
	Mean Intuitive Eating Scale	2.95	0.60	1.05	4.86	2.95	2.94	Continous, see question	Intuitive Eating Scale
	Missing values for Short Self-Regulation Questionna	0.14		0.00	1.00	0.14	0.13	Binary	
	Short Self-Regulation Questionnaire total score	89.92	14.06	35.00	130.00	90.02	89.53	Continous, see question	Short Self-Regulation Questionnaire (SSRQ)
	Fruit and vegetable portions last week	2.89	2.03	0.00	15.21	2.88	2.90	Ordinal, see question	How many fist-sized portions of fresh fruit have you eaten?<br>How many fist-sized portions of fresh vegetables (raw or cooked) have you eaten?<br>How many fist-sized portions of frozen vegetables have you eaten?<br>How many glasses (0.2l) of fruit juice or smoothies have you drunk?<br>0 None in the last 7 days<br>2/7 = 1-3 in the last 7 days<br>5/7 = 4-6 in the last 7 days<br>1 = about 1 a day<br>2 = about 2 a day<br>3 = about 3 a day<br>5 = 4 or more a day

Category	Variables	Total Sample Mean	Std	Min	Max	Training Mean	Test Mean	Units	Original Question/ Categories Used
**Simple and Extended Behavior Data**	Login day 0	0.89	0.31	0.00	1.00	0.89	0.91	Count logins	
	Login day 1	0.42	0.49	0.00	1.00	0.41	0.45	Count logins	
	Login day 2	0.32	0.47	0.00	1.00	0.32	0.31	Count logins	
	Login day 3	0.28	0.45	0.00	1.00	0.29	0.26	Count logins	
	Login day 4	0.26	0.44	0.00	1.00	0.27	0.26	Count logins	
	Login day 5	0.27	0.45	0.00	1.00	0.27	0.28	Count logins	
	Login day 6	0.27	0.44	0.00	1.00	0.27	0.27	Count logins	
**Selected Behavior Data**	Days for module1	2.50	3.14	0.00	8.00	2.50	2.49	Days	
	Days for module2	5.82	3.45	0.00	8.00	5.77	6.02	Days	
	Length answers week1	358	981	0	12112	363	336	Characters	
	Number of answers week1	6.56	8.00	0.00	39.00	6.55	6.63	Answers	
	Seconds spent logged in week1	8249	9020	0	110349	8239	8286	Seconds	
	Days logged in week1	2.72	1.94	0.00	7.00	2.72	2.73	Days	
	Length messages to coach week1	70	465	0	9641	71	65	Characters	
	Length messages to group week1	116	606	0	14573	111	135	Characters	
	Number messages to coach week1	0.07	0.35	0.00	10.00	0.07	0.06	Messages	
	Number messages to group week1	0.22	1.00	0.00	22.00	0.22	0.23	Messages	
	Number of closed question diary entries week1	1.81	2.58	0.00	20.00	1.80	1.85	Diary entries	
	Number open-text diary entries week1	1.81	2.58	0.00	20.00	1.80	1.85	Diary entries	
	Length open-text diary entries week1	1	25	0	1331	1	0	Characters	
**Extended Behavior Data**	Sessions completed day 1	0.21		0.00	1.00	0.20	0.25	Binary	
	Sessions completed day 2	0.12		0.00	1.00	0.12	0.11	Binary	
	Sessions completed day 3	0.07		0.00	1.00	0.07	0.06	Binary	
	Sessions completed day 4	0.04		0.00	1.00	0.04	0.03	Binary	
	Sessions completed day 5	0.04		0.00	1.00	0.04	0.04	Binary	
	Sessions completed day 6	0.03		0.00	1.00	0.03	0.03	Binary	
	Sessions completed day 7	0.04		0.00	1.00	0.04	0.03	Binary	
	Length of answers day 1	115	572	0	11086	106	153	Characters	
	Length of answers day 2	86	513	0	11385	94	51	Characters	
	Length of answers day 3	53	425	0	7558	55	48	Characters	
	Length of answers day 4	37	381	0	12112	41	22	Characters	
	Length of answers day 5	24	236	0	4846	24	24	Characters	
	Length of answers day 6	16	169	0	3964	16	14	Characters	
	Length of answers day 7	27	253	0	6179	27	24	Characters	
	Number of answers day 1	2.41	5.62	0.00	37.00	2.29	2.90	Answers	
	Number of answers day 2	1.49	4.84	0.00	36.00	1.55	1.26	Answers	
	Number of answers day 3	0.84	3.65	0.00	37.00	0.84	0.83	Answers	
	Number of answers day 4	0.52	2.89	0.00	29.00	0.54	0.41	Answers	
	Number of answers day 5	0.46	2.72	0.00	27.00	0.45	0.51	Answers	
	Number of answers day 6	0.36	2.29	0.00	24.00	0.36	0.33	Answers	
	Number of answers day 7	0.48	2.86	0.00	39.00	0.50	0.39	Answers	
	Seconds spent logged in day 1	3873	4318	0	37867	3815	4105	Seconds	
	Seconds spent logged in day 2	1438	3252	0	33735	1407	1563	Seconds	
	Seconds spent logged in day 3	812	2422	0	30354	844	683	Seconds	
	Seconds spent logged in day 4	636	2187	0	38268	630	657	Seconds	
	Seconds spent logged in day 5	476	1826	0	29083	482	452	Seconds	
	Seconds spent logged in day 6	496	1898	0	35100	516	418	Seconds	
	Seconds spent logged in day 7	518	2024	0	32735	546	408	Seconds	
	Seconds spent logged in FrSaSun	3014	5082	0	40812	2921	3383	Seconds	
	Seconds spent logged in MoTu	2815	4797	0	61380	2848	2681	Seconds	
	Seconds spent logged in WedThur	2420	4440	0	52811	2470	2222	Seconds	
	Seconds spent logged in during day	3021	4573	0	47229	3000	3106	Seconds	
	Seconds spent logged in during evening	3066	5090	0	57441	3105	2908	Seconds	
	Seconds spent logged in during morning	2162	3665	0	32582	2134	2273	Seconds	
	Pages day1	63	65	0	626	62	67	# of pages requested to server	

Category	Variables	Total Sample Mean	Std	Min	Max	Training Mean	Test Mean	Units	Original Question/ Categories Used
**Extended Behavior Data**	Pages day1	63	65	0	626	62	67	# of pages requested to server	
	Pages day2	29	54	0	586	28	31	# of pages requested to server	
	Pages day3	20	44	0	607	20	19	# of pages requested to server	
	Pages day4	16	38	0	474	16	15	# of pages requested to server	
	Pages day5	14	36	0	567	14	15	# of pages requested to server	
	Pages day6	14	36	0	675	14	13	# of pages requested to server	
	Pages day7	14	34	0	448	15	13	# of pages requested to server	
	Length messages to coach day0	2	102	0	5655	3	0	Characters	
	Length messages to coach day1	4	101	0	4614	5	0	Characters	
	Length messages to coach day2	6	111	0	4172	6	7	Characters	
	Length messages to coach day3	9	159	0	5888	10	3	Characters	
	Length messages to coach day4	9	132	0	4632	7	15	Characters	
	Length messages to coach day5	19	288	0	9641	19	18	Characters	
	Length messages to coach day6	21	229	0	4862	21	22	Characters	
	Length messages to group day0	15	159	0	5925	13	24	Characters	
	Length messages to group day1	21	206	0	5691	18	30	Characters	
	Length messages to group day2	21	197	0	4716	19	27	Characters	
	Length messages to group day3	17	213	0	7729	18	13	Characters	
	Length messages to group day4	12	124	0	3579	10	19	Characters	
	Length messages to group day5	13	137	0	3813	14	10	Characters	
	Length messages to group day6	17	164	0	3250	18	13	Characters	
	Number messages to coach day0	0.00	0.05	0.00	2.00	0.00	0.00	Messages	
	Number messages to coach day1	0.01	0.08	0.00	2.00	0.01	0.00	Messages	
	Number messages to coach day2	0.01	0.10	0.00	2.00	0.01	0.01	Messages	
	Number messages to coach day3	0.01	0.18	0.00	7.00	0.01	0.01	Messages	
	Number messages to coach day4	0.01	0.11	0.00	3.00	0.01	0.01	Messages	
	Number messages to coach day5	0.01	0.13	0.00	2.00	0.02	0.01	Messages	
	Number messages to coach day6	0.02	0.14	0.00	3.00	0.02	0.02	Messages	
	Number messages to group day0	0.03	0.22	0.00	4.00	0.02	0.03	Messages	
	Number messages to group day1	0.04	0.30	0.00	5.00	0.04	0.05	Messages	
	Number messages to group day2	0.04	0.30	0.00	5.00	0.04	0.05	Messages	
	Number messages to group day3	0.03	0.24	0.00	7.00	0.03	0.03	Messages	
	Number messages to group day4	0.02	0.19	0.00	4.00	0.02	0.03	Messages	
	Number messages to group day5	0.03	0.25	0.00	5.00	0.03	0.02	Messages	
	Number messages to group day6	0.03	0.27	0.00	6.00	0.03	0.02	Messages	
	Number of diaries submitted day1	0.23	0.50	0.00	4.00	0.23	0.23	Diary entries	
	Number of diaries submitted day2	0.27	0.58	0.00	13.00	0.26	0.30	Diary entries	
	Number of diaries submitted day3	0.27	0.49	0.00	3.00	0.27	0.27	Diary entries	
	Number of diaries submitted day4	0.25	0.47	0.00	4.00	0.24	0.26	Diary entries	
	Number of diaries submitted day5	0.26	0.48	0.00	3.00	0.26	0.26	Diary entries	
	Number of diaries submitted day6	0.26	0.47	0.00	3.00	0.26	0.27	Diary entries	
	Number of diaries submitted day7	0.27	0.49	0.00	3.00	0.28	0.25	Diary entries	
	Length of diaries submitted week 1	1	25	0	1331	1	0	Characters	
	Mean question 1	0.00	0.06	0.00	1.00	0.01	0.00	Continous	
	Min question 1	0.00	0.05	0.00	1.00	0.00	0.00	Continous	
	Max question 1	0.01	0.09	0.00	1.00	0.01	0.01	Continous	
	Mean question 2	0.00	0.05	0.00	1.00	0.00	0.00	Continous	
	Min question 2	0.00	0.04	0.00	1.00	0.00	0.00	Continous	
	Max question 2	0.01	0.08	0.00	1.00	0.01	0.01	Continous	

Supplementary Table 2 – Result metrics

Average across runs

	Test Performance							CV Performance		Diff.
	f1 score	accuracy	recall	precision	BACC	AUC	AUC SD	AUC	SD AUC	Train-Test
**('wcs_only', 100, 'lr')**	0.4754	0.4717	0.3821	0.6290	0.5020	0.5048	0.0186	0.5768	0.2115	0.0720
**('wcs_only', 100, 'svm')**	0.5913	0.5254	0.5478	0.6422	0.5179	0.5073	0.0312	0.5990	0.2357	0.0917
**('wcs_only', 100, 'nb')**	0.5619	0.5090	0.5026	0.6371	0.5112	0.5188	0.0217	0.5618	0.2459	0.0430
**('wcs_only', 100, 'rf')**	0.5833	0.5144	0.5426	0.6307	0.5048	0.5130	0.0220	0.6343	0.2305	0.1213
**('wcs_only', 100, 'adaboost')**	0.6968	0.5740	0.7812	0.6288	0.5038	0.5065	0.0193	0.6070	0.2224	0.1005
**('wcs_only', 100, 'nn')**	0.6711	0.5572	0.7212	0.6276	0.5016	0.5008	0.0235	0.6050	0.2362	0.1042
**('wcs_only', 200, 'lr')**	0.5293	0.4947	0.4535	0.6356	0.5086	0.5092	0.0210	0.5743	0.1631	0.0651
**('wcs_only', 200, 'svm')**	0.5546	0.5055	0.4915	0.6364	0.5102	0.5110	0.0196	0.5864	0.1561	0.0753
**('wcs_only', 200, 'nb')**	0.5584	0.5074	0.4972	0.6369	0.5109	0.5106	0.0235	0.5425	0.1624	0.0319
**('wcs_only', 200, 'rf')**	0.6051	0.5279	0.5773	0.6357	0.5112	0.5175	0.0166	0.6010	0.1586	0.0835
**('wcs_only', 200, 'adaboost')**	0.7065	0.5828	0.8015	0.6316	0.5086	0.5112	0.0171	0.5704	0.1457	0.0593
**('wcs_only', 200, 'nn')**	0.7058	0.5855	0.7934	0.6355	0.5150	0.5104	0.0217	0.5711	0.1468	0.0607
**('wcs_only', 300, 'lr')**	0.5841	0.5196	0.5384	0.6382	0.5132	0.5104	0.0247	0.5524	0.1347	0.0420
**('wcs_only', 300, 'svm')**	0.6100	0.5320	0.5841	0.6383	0.5144	0.5064	0.0317	0.5702	0.1325	0.0638
**('wcs_only', 300, 'nb')**	0.6372	0.5421	0.6417	0.6327	0.5084	0.5144	0.0276	0.5385	0.1547	0.0242
**('wcs_only', 300, 'rf')**	0.6205	0.5337	0.6085	0.6330	0.5083	0.5055	0.0218	0.5676	0.1366	0.0620
**('wcs_only', 300, 'adaboost')**	0.7458	0.6096	0.9140	0.6299	0.5064	0.5123	0.0213	0.5461	0.1249	0.0338
**('wcs_only', 300, 'nn')**	0.7347	0.6040	0.8753	0.6330	0.5120	0.5148	0.0246	0.5624	0.1403	0.0476
**('wcs_only', 400, 'lr')**	0.5525	0.5109	0.4819	0.6474	0.5208	0.5200	0.0278	0.5708	0.1169	0.0508
**('wcs_only', 400, 'svm')**	0.5586	0.5153	0.4895	0.6504	0.5241	0.5304	0.0161	0.5771	0.1069	0.0466
**('wcs_only', 400, 'nb')**	0.6602	0.5599	0.6823	0.6395	0.5184	0.5210	0.0246	0.5589	0.1269	0.0379
**('wcs_only', 400, 'rf')**	0.6011	0.5248	0.5714	0.6340	0.5090	0.5171	0.0255	0.5839	0.1024	0.0668
**('wcs_only', 400, 'adaboost')**	0.7543	0.6145	0.9445	0.6279	0.5027	0.5193	0.0261	0.5627	0.1121	0.0434

	Test Performance							CV Performance		Diff.
	f1 score	accuracy	recall	precision	BACC	AUC	AUC SD	AUC	SD AUC	Train-Test
**('wcs_only', 400, 'nn')**	0.7366	0.6063	0.8788	0.6341	0.5140	0.5190	0.0195	0.5648	0.1048	0.0458
**('wcs_only', 500, 'lr')**	0.5731	0.5224	0.5116	0.6514	0.5261	0.5244	0.0238	0.5686	0.1044	0.0442
**('wcs_only', 500, 'svm')**	0.5689	0.5226	0.5028	0.6550	0.5293	0.5212	0.0371	0.5747	0.0976	0.0534
**('wcs_only', 500, 'nb')**	0.6459	0.5528	0.6511	0.6409	0.5195	0.5246	0.0243	0.5663	0.0976	0.0417
**('wcs_only', 500, 'rf')**	0.5987	0.5279	0.5620	0.6405	0.5164	0.5200	0.0225	0.5759	0.1042	0.0558
**('wcs_only', 500, 'adaboost')**	0.7647	0.6239	0.9753	0.6289	0.5049	0.5238	0.0197	0.5644	0.0944	0.0406
**('wcs_only', 500, 'nn')**	0.7513	0.6133	0.9321	0.6292	0.5052	0.5241	0.0261	0.5648	0.1006	0.0407
**('wcs_only', 750, 'lr')**	0.5798	0.5324	0.5148	0.6635	0.5384	0.5347	0.0171	0.5762	0.0757	0.0416
**('wcs_only', 750, 'svm')**	0.5849	0.5335	0.5245	0.6610	0.5366	0.5362	0.0124	0.5769	0.0757	0.0407
**('wcs_only', 750, 'nb')**	0.6785	0.5732	0.7188	0.6425	0.5239	0.5317	0.0178	0.5634	0.0782	0.0317
**('wcs_only', 750, 'rf')**	0.6144	0.5354	0.5908	0.6400	0.5167	0.5238	0.0270	0.5783	0.0808	0.0545
**('wcs_only', 750, 'adaboost')**	0.7554	0.6156	0.9476	0.6281	0.5031	0.5254	0.0074	0.5711	0.0775	0.0457
**('wcs_only', 750, 'nn')**	0.7527	0.6161	0.9323	0.6311	0.5090	0.5294	0.0169	0.5631	0.0711	0.0337
**('wcs_only', 1000, 'lr')**	0.5733	0.5207	0.5140	0.6481	0.5229	0.5246	0.0162	0.5737	0.0690	0.0492
**('wcs_only', 1000, 'svm')**	0.5847	0.5297	0.5284	0.6544	0.5301	0.5318	0.0108	0.5873	0.0564	0.0555
**('wcs_only', 1000, 'nb')**	0.6774	0.5729	0.7157	0.6430	0.5245	0.5281	0.0154	0.5705	0.0646	0.0424
**('wcs_only', 1000, 'rf')**	0.6093	0.5280	0.5873	0.6329	0.5080	0.5182	0.0226	0.5731	0.0620	0.0548
**('wcs_only', 1000, 'adaboost')**	0.7544	0.6161	0.9410	0.6296	0.5061	0.5341	0.0125	0.5667	0.0624	0.0326
**('wcs_only', 1000, 'nn')**	0.7453	0.6082	0.9148	0.6288	0.5043	0.5241	0.0210	0.5752	0.0537	0.0511
**('wcs_only', 1500, 'lr')**	0.5821	0.5239	0.5293	0.6467	0.5221	0.5250	0.0099	0.5716	0.0502	0.0466
**('wcs_only', 1500, 'svm')**	0.6035	0.5365	0.5629	0.6504	0.5276	0.5287	0.0103	0.5859	0.0507	0.0573
**('wcs_only', 1500, 'nb')**	0.6757	0.5694	0.7162	0.6396	0.5196	0.5302	0.0118	0.5613	0.0491	0.0311
**('wcs_only', 1500, 'rf')**	0.6244	0.5393	0.6114	0.6381	0.5148	0.5181	0.0079	0.5738	0.0478	0.0557
**('wcs_only', 1500, 'adaboost')**	0.7704	0.6265	1.0000	0.6265	0.5000	0.5262	0.0071	0.5668	0.0518	0.0406
**('wcs_only', 1500, 'nn')**	0.7488	0.6107	0.9262	0.6284	0.5038	0.5215	0.0116	0.5765	0.0485	0.0551
**('wcs_only', 2000, 'lr')**	0.5882	0.5283	0.5376	0.6493	0.5252	0.5241	0.0094	0.5746	0.0452	0.0505
**('wcs_only', 2000, 'svm')**	0.6008	0.5412	0.5511	0.6604	0.5378	0.5288	0.0085	0.5839	0.0390	0.0551

	Test Performance							CV Performance		Diff.
	f1 score	accuracy	recall	precision	BACC	AUC	AUC SD	AUC	SD AUC	Train-Test
**('wcs_only', 2000, 'nb')**	0.6665	0.5699	0.6860	0.6481	0.5306	0.5289	0.0126	0.5660	0.0439	0.0372
**('wcs_only', 2000, 'adaboost')**	0.7696	0.6254	0.9983	0.6261	0.4991	0.5237	0.0107	0.5737	0.0455	0.0500
**('wcs_only', 2000, 'rf')**	0.6219	0.5395	0.6044	0.6404	0.5176	0.5181	0.0238	0.5728	0.0427	0.0547
**('wcs_only', 2000, 'nn')**	0.7604	0.6208	0.9603	0.6294	0.5058	0.5258	0.0081	0.5756	0.0432	0.0497
**('wcs_only', 2500, 'lr')**	0.5877	0.5271	0.5378	0.6477	0.5235	0.5272	0.0038	0.5754	0.0462	0.0482
**('wcs_only', 2500, 'svm')**	0.5985	0.5399	0.5473	0.6602	0.5374	0.5252	0.0102	0.5837	0.0465	0.0586
**('wcs_only', 2500, 'nb')**	0.6655	0.5663	0.6885	0.6440	0.5250	0.5265	0.0052	0.5674	0.0435	0.0409
**('wcs_only', 2500, 'rf')**	0.6229	0.5367	0.6106	0.6356	0.5117	0.5212	0.0106	0.5772	0.0483	0.0560
**('wcs_only', 2500, 'adaboost')**	0.7690	0.6261	0.9934	0.6273	0.5016	0.5288	0.0064	0.5699	0.0418	0.0411
**('wcs_only', 2500, 'nn')**	0.7578	0.6179	0.9541	0.6285	0.5039	0.5276	0.0173	0.5803	0.0466	0.0527
**('wcs_only', 3000, 'lr')**	0.5787	0.5212	0.5247	0.6449	0.5200	0.5308	0.0036	0.5763	0.0445	0.0455
**('wcs_only', 3000, 'svm')**	0.6032	0.5422	0.5553	0.6600	0.5377	0.5290	0.0067	0.5843	0.0444	0.0553
**('wcs_only', 3000, 'nb')**	0.6648	0.5673	0.6849	0.6458	0.5274	0.5298	0.0081	0.5699	0.0461	0.0401
**('wcs_only', 3000, 'rf')**	0.6169	0.5344	0.5983	0.6367	0.5128	0.5172	0.0038	0.5774	0.0443	0.0602
**('wcs_only', 3000, 'adaboost')**	0.7701	0.6261	0.9993	0.6264	0.4996	0.5239	0.0038	0.5755	0.0403	0.0515
**('wcs_only', 3000, 'nn')**	0.7593	0.6179	0.9622	0.6271	0.5012	0.5248	0.0131	0.5847	0.0433	0.0599
**('wcs_only', 3651, 'lr')**	0.5780	0.5226	0.5218	0.6477	0.5228	0.5303	0.0002	0.5755	0.0327	0.0452
**('wcs_only', 3651, 'svm')**	0.5984	0.5367	0.5509	0.6548	0.5319	0.5311	0.0010	0.5862	0.0328	0.0552
**('wcs_only', 3651, 'nb')**	0.6624	0.5705	0.6725	0.6525	0.5359	0.5292	0.0000	0.5691	0.0333	0.0400
**('wcs_only', 3651, 'rf')**	0.5801	0.5089	0.5415	0.6247	0.4978	0.5098	0.0079	0.5809	0.0361	0.0711
**('wcs_only', 3651, 'adaboost')**	0.7694	0.6252	0.9978	0.6260	0.4989	0.5373	0.0040	0.5733	0.0367	0.0360
**('wcs_only', 3651, 'nn')**	0.7602	0.6188	0.9643	0.6274	0.5017	0.5320	0.0031	0.5831	0.0282	0.0511
**('baseline_extended', 100, 'lr')**	0.6271	0.5546	0.5978	0.6594	0.5399	0.5648	0.0192	0.6257	0.2376	0.0609
**('baseline_extended', 100, 'svm')**	0.5863	0.5313	0.5301	0.6559	0.5317	0.5326	0.0600	0.6420	0.2145	0.1094
**('baseline_extended', 100, 'nb')**	0.5253	0.5100	0.4328	0.6682	0.5362	0.5783	0.0342	0.5940	0.2307	0.0157
**('baseline_extended', 100, 'rf')**	0.6786	0.5758	0.7146	0.6459	0.5287	0.5541	0.0344	0.6720	0.2065	0.1179
**('baseline_extended', 100, 'adaboost')**	0.6681	0.5714	0.6884	0.6489	0.5318	0.5506	0.0265	0.6492	0.2262	0.0986

	Test Performance							CV Performance		Diff.
	f1 score	accuracy	recall	precision	BACC	AUC	AUC SD	AUC	SD AUC	Train-Test
**('baseline_extended', 100, 'nn')**	0.6951	0.5851	0.7548	0.6441	0.5276	0.5622	0.0368	0.6446	0.2118	0.0824
**('baseline_extended', 200, 'lr')**	0.6276	0.5732	0.5740	0.6922	0.5729	0.5982	0.0260	0.6134	0.1509	0.0152
**('baseline_extended', 200, 'svm')**	0.6329	0.5659	0.5972	0.6731	0.5554	0.5461	0.0750	0.6256	0.1602	0.0795
**('baseline_extended', 200, 'nb')**	0.5670	0.5357	0.4852	0.6820	0.5528	0.5981	0.0280	0.6119	0.1516	0.0138
**('baseline_extended', 200, 'rf')**	0.6375	0.5694	0.6044	0.6745	0.5575	0.5862	0.0313	0.6531	0.1480	0.0669
**('baseline_extended', 200, 'adaboost')**	0.6782	0.5815	0.7037	0.6544	0.5401	0.5723	0.0222	0.6471	0.1464	0.0748
**('baseline_extended', 200, 'nn')**	0.6825	0.5856	0.7107	0.6564	0.5433	0.5755	0.0349	0.6157	0.1327	0.0403
**('baseline_extended', 300, 'lr')**	0.6189	0.5744	0.5515	0.7050	0.5822	0.6178	0.0203	0.6239	0.1284	0.0061
**('baseline_extended', 300, 'svm')**	0.6493	0.5815	0.6183	0.6836	0.5691	0.5979	0.0289	0.6261	0.1261	0.0282
**('baseline_extended', 300, 'nb')**	0.5846	0.5569	0.4976	0.7084	0.5770	0.6094	0.0202	0.6050	0.1357	-0.0043
**('baseline_extended', 300, 'rf')**	0.6683	0.5886	0.6614	0.6754	0.5640	0.6034	0.0289	0.6381	0.1276	0.0347
**('baseline_extended', 300, 'adaboost')**	0.6746	0.5796	0.6954	0.6549	0.5404	0.5800	0.0282	0.6244	0.1169	0.0443
**('baseline_extended', 300, 'nn')**	0.6821	0.5878	0.7059	0.6599	0.5478	0.5885	0.0221	0.6255	0.1279	0.0370
**('baseline_extended', 400, 'lr')**	0.6223	0.5793	0.5531	0.7113	0.5882	0.6260	0.0178	0.6211	0.1136	-0.0049
**('baseline_extended', 400, 'svm')**	0.6390	0.5789	0.5948	0.6903	0.5736	0.6066	0.0232	0.6315	0.1057	0.0249
**('baseline_extended', 400, 'nb')**	0.6275	0.5772	0.5686	0.7002	0.5801	0.6140	0.0251	0.6185	0.1101	0.0045
**('baseline_extended', 400, 'rf')**	0.6747	0.5960	0.6688	0.6808	0.5714	0.6169	0.0173	0.6379	0.1051	0.0210
**('baseline_extended', 400, 'adaboost')**	0.7338	0.6141	0.8489	0.6462	0.5345	0.6014	0.0200	0.6237	0.0953	0.0223
**('baseline_extended', 400, 'nn')**	0.7005	0.6082	0.7314	0.6722	0.5665	0.6026	0.0189	0.6191	0.0974	0.0165
**('baseline_extended', 500, 'lr')**	0.6254	0.5810	0.5583	0.7109	0.5887	0.6311	0.0183	0.6332	0.0917	0.0021
**('baseline_extended', 500, 'svm')**	0.6454	0.5876	0.5991	0.6995	0.5836	0.6113	0.0215	0.6429	0.0879	0.0316
**('baseline_extended', 500, 'nb')**	0.6245	0.5778	0.5603	0.7053	0.5838	0.6182	0.0197	0.6318	0.0918	0.0137
**('baseline_extended', 500, 'rf')**	0.6519	0.5854	0.6197	0.6877	0.5737	0.6176	0.0171	0.6514	0.0920	0.0338
**('baseline_extended', 500, 'adaboost')**	0.7359	0.6176	0.8502	0.6487	0.5388	0.6080	0.0232	0.6266	0.0936	0.0186
**('baseline_extended', 500, 'nn')**	0.6722	0.5847	0.6797	0.6649	0.5525	0.6081	0.0250	0.6283	0.0948	0.0202
**('baseline_extended', 750, 'lr')**	0.6240	0.5882	0.5454	0.7291	0.6027	0.6469	0.0201	0.6600	0.0775	0.0131
**('baseline_extended', 750, 'svm')**	0.6321	0.5830	0.5716	0.7068	0.5869	0.6246	0.0162	0.6560	0.0731	0.0314

	Test Performance							CV Performance		Diff.
	f1 score	accuracy	recall	precision	BACC	AUC	AUC SD	AUC	SD AUC	Train-Test
**('baseline_extended', 750, 'nb')**	0.6443	0.5915	0.5904	0.7090	0.5919	0.6299	0.0107	0.6406	0.0817	0.0108
**('baseline_extended', 750, 'rf')**	0.6593	0.5929	0.6288	0.6930	0.5807	0.6346	0.0158	0.6689	0.0772	0.0343
**('baseline_extended', 750, 'adaboost')**	0.7310	0.6153	0.8341	0.6505	0.5412	0.6130	0.0191	0.6540	0.0638	0.0410
**('baseline_extended', 750, 'nn')**	0.7123	0.6175	0.7559	0.6735	0.5706	0.6263	0.0153	0.6428	0.0781	0.0165
**('baseline_extended', 1000, 'lr')**	0.6259	0.5899	0.5476	0.7303	0.6042	0.6493	0.0164	0.6660	0.0612	0.0167
**('baseline_extended', 1000, 'svm')**	0.6270	0.5847	0.5572	0.7169	0.5940	0.6371	0.0153	0.6627	0.0603	0.0256
**('baseline_extended', 1000, 'nb')**	0.6474	0.5918	0.5983	0.7055	0.5896	0.6317	0.0126	0.6464	0.0664	0.0148
**('baseline_extended', 1000, 'rf')**	0.6528	0.5948	0.6079	0.7048	0.5904	0.6387	0.0158	0.6689	0.0675	0.0302
**('baseline_extended', 1000, 'adaboost')**	0.7415	0.6227	0.8638	0.6496	0.5410	0.6305	0.0163	0.6584	0.0596	0.0280
**('baseline_extended', 1000, 'nn')**	0.6332	0.5625	0.6026	0.6670	0.5489	0.5906	0.0326	0.6480	0.0610	0.0574
**('baseline_extended', 1500, 'lr')**	0.6307	0.5970	0.5493	0.7404	0.6131	0.6511	0.0072	0.6676	0.0537	0.0165
**('baseline_extended', 1500, 'svm')**	0.6140	0.5814	0.5314	0.7270	0.5983	0.6417	0.0112	0.6656	0.0515	0.0239
**('baseline_extended', 1500, 'nb')**	0.6426	0.5882	0.5908	0.7043	0.5874	0.6351	0.0082	0.6470	0.0517	0.0119
**('baseline_extended', 1500, 'rf')**	0.6637	0.6008	0.6288	0.7028	0.5913	0.6419	0.0095	0.6698	0.0566	0.0278
**('baseline_extended', 1500, 'adaboost')**	0.7470	0.6238	0.8865	0.6455	0.5348	0.6416	0.0172	0.6594	0.0467	0.0178
**('baseline_extended', 1500, 'nn')**	0.6332	0.5696	0.5930	0.6793	0.5617	0.6334	0.0291	0.6435	0.0545	0.0102
**('baseline_extended', 2000, 'lr')**	0.6284	0.5940	0.5480	0.7365	0.6095	0.6528	0.0061	0.6769	0.0426	0.0242
**('baseline_extended', 2000, 'svm')**	0.6146	0.5811	0.5332	0.7255	0.5974	0.6434	0.0093	0.6747	0.0425	0.0313
**('baseline_extended', 2000, 'nb')**	0.6454	0.5902	0.5952	0.7048	0.5884	0.6340	0.0048	0.6492	0.0431	0.0152
**('baseline_extended', 2000, 'rf')**	0.6688	0.6033	0.6393	0.7011	0.5911	0.6411	0.0103	0.6716	0.0439	0.0304
**('baseline_extended', 2000, 'adaboost')**	0.7485	0.6356	0.8655	0.6593	0.5577	0.6546	0.0084	0.6675	0.0452	0.0128
**('baseline_extended', 2000, 'nn')**	0.7244	0.6202	0.7965	0.6642	0.5605	0.6344	0.0142	0.6537	0.0490	0.0193
**('baseline_extended', 2500, 'lr')**	0.6297	0.5978	0.5459	0.7440	0.6154	0.6569	0.0028	0.6796	0.0411	0.0227
**('baseline_extended', 2500, 'svm')**	0.6209	0.5873	0.5393	0.7315	0.6036	0.6485	0.0029	0.6741	0.0426	0.0256
**('baseline_extended', 2500, 'nb')**	0.6511	0.5919	0.6077	0.7011	0.5865	0.6354	0.0032	0.6546	0.0441	0.0192
**('baseline_extended', 2500, 'rf')**	0.6592	0.5969	0.6223	0.7008	0.5883	0.6435	0.0046	0.6780	0.0450	0.0345
**('baseline_extended', 2500, 'adaboost')**	0.7487	0.6352	0.8675	0.6586	0.5565	0.6560	0.0044	0.6729	0.0356	0.0169

	Test Performance							CV Performance		Diff.
	f1 score	accuracy	recall	precision	BACC	AUC	AUC SD	AUC	SD AUC	Train-Test
**('baseline_extended', 2500, 'nn')**	0.6999	0.6133	0.7198	0.6811	0.5772	0.6337	0.0157	0.6613	0.0429	0.0277
**('baseline_extended', 3000, 'lr')**	0.6407	0.6083	0.5575	0.7532	0.6255	0.6566	0.0014	0.6801	0.0354	0.0234
**('baseline_extended', 3000, 'svm')**	0.6308	0.5964	0.5502	0.7390	0.6121	0.6500	0.0026	0.6777	0.0356	0.0277
**('baseline_extended', 3000, 'nb')**	0.6445	0.5860	0.5990	0.6975	0.5815	0.6355	0.0031	0.6558	0.0370	0.0203
**('baseline_extended', 3000, 'rf')**	0.6615	0.5996	0.6245	0.7033	0.5912	0.6445	0.0030	0.6747	0.0396	0.0302
**('baseline_extended', 3000, 'adaboost')**	0.7476	0.6338	0.8654	0.6580	0.5554	0.6567	0.0075	0.6754	0.0325	0.0186
**('baseline_extended', 3000, 'nn')**	0.7241	0.6265	0.7824	0.6740	0.5737	0.6433	0.0122	0.6651	0.0309	0.0218
**('baseline_extended', 3651, 'lr')**	0.6288	0.5974	0.5444	0.7443	0.6153	0.6571	0.0007	0.6783	0.0339	0.0212
**('baseline_extended', 3651, 'svm')**	0.6358	0.6019	0.5546	0.7449	0.6180	0.6506	0.0002	0.6774	0.0350	0.0268
**('baseline_extended', 3651, 'nb')**	0.6464	0.5869	0.6026	0.6970	0.5815	0.6355	0.0001	0.6517	0.0336	0.0162
**('baseline_extended', 3651, 'rf')**	0.6562	0.6005	0.6084	0.7121	0.5979	0.6474	0.0033	0.6737	0.0321	0.0263
**('baseline_extended', 3651, 'adaboost')**	0.7437	0.6347	0.8457	0.6636	0.5633	0.6641	0.0017	0.6773	0.0331	0.0132
**('baseline_extended', 3651, 'nn')**	0.7242	0.6224	0.7911	0.6677	0.5653	0.6341	0.0132	0.6599	0.0301	0.0257
**('behavior_simple', 100, 'lr')**	0.6553	0.6029	0.6024	0.7183	0.6030	0.6375	0.0562	0.6865	0.2018	0.0490
**('behavior_simple', 100, 'svm')**	0.6934	0.6241	0.6786	0.7089	0.6056	0.6279	0.0875	0.7252	0.2117	0.0973
**('behavior_simple', 100, 'nb')**	0.6048	0.5610	0.5360	0.6937	0.5695	0.6360	0.0269	0.6663	0.2334	0.0303
**('behavior_simple', 100, 'rf')**	0.6855	0.6187	0.6631	0.7094	0.6037	0.6436	0.0381	0.7391	0.1864	0.0954
**('behavior_simple', 100, 'adaboost')**	0.7399	0.6472	0.8011	0.6875	0.5951	0.6335	0.0402	0.6928	0.2062	0.0593
**('behavior_simple', 100, 'nn')**	0.7101	0.6237	0.7358	0.6862	0.5857	0.6259	0.0561	0.6845	0.2314	0.0586
**('behavior_simple', 200, 'lr')**	0.7063	0.6416	0.6878	0.7258	0.6259	0.6755	0.0215	0.7023	0.1451	0.0267
**('behavior_simple', 200, 'svm')**	0.7240	0.6521	0.7284	0.7197	0.6263	0.6813	0.0151	0.7148	0.1428	0.0335
**('behavior_simple', 200, 'nb')**	0.6476	0.5951	0.5939	0.7120	0.5955	0.6416	0.0235	0.6689	0.1527	0.0273
**('behavior_simple', 200, 'rf')**	0.6868	0.6249	0.6563	0.7202	0.6142	0.6669	0.0244	0.7355	0.1368	0.0686
**('behavior_simple', 200, 'adaboost')**	0.7580	0.6661	0.8345	0.6943	0.6090	0.6737	0.0242	0.7020	0.1457	0.0283
**('behavior_simple', 200, 'nn')**	0.7341	0.6458	0.7803	0.6930	0.6002	0.6539	0.0248	0.6981	0.1449	0.0442
**('behavior_simple', 300, 'lr')**	0.7197	0.6516	0.7140	0.7255	0.6304	0.6779	0.0246	0.7196	0.1364	0.0417
**('behavior_simple', 300, 'svm')**	0.7205	0.6517	0.7164	0.7246	0.6298	0.6722	0.0274	0.7162	0.1330	0.0440

	Test Performance							CV Performance		Diff.
	f1 score	accuracy	recall	precision	BACC	AUC	AUC SD	AUC	SD AUC	Train-Test
**('behavior_simple', 300, 'nb')**	0.6907	0.6259	0.6668	0.7164	0.6120	0.6434	0.0306	0.6879	0.1386	0.0445
**('behavior_simple', 300, 'rf')**	0.6991	0.6349	0.6769	0.7228	0.6207	0.6735	0.0192	0.7339	0.1305	0.0604
**('behavior_simple', 300, 'adaboost')**	0.7654	0.6721	0.8539	0.6936	0.6105	0.6800	0.0239	0.7207	0.1357	0.0407
**('behavior_simple', 300, 'nn')**	0.7479	0.6585	0.8085	0.6958	0.6077	0.6677	0.0330	0.7120	0.1258	0.0443
**('behavior_simple', 400, 'lr')**	0.7131	0.6486	0.6972	0.7298	0.6321	0.6878	0.0200	0.7207	0.0996	0.0329
**('behavior_simple', 400, 'svm')**	0.7140	0.6480	0.7011	0.7273	0.6300	0.6795	0.0293	0.7197	0.0928	0.0401
**('behavior_simple', 400, 'nb')**	0.7305	0.6587	0.7382	0.7229	0.6317	0.6509	0.0261	0.6983	0.1059	0.0473
**('behavior_simple', 400, 'rf')**	0.6930	0.6335	0.6600	0.7293	0.6245	0.6812	0.0174	0.7317	0.0950	0.0505
**('behavior_simple', 400, 'adaboost')**	0.7671	0.6737	0.8574	0.6939	0.6115	0.6925	0.0180	0.7276	0.0985	0.0351
**('behavior_simple', 400, 'nn')**	0.7394	0.6503	0.7917	0.6936	0.6024	0.6735	0.0200	0.7110	0.0976	0.0376
**('behavior_simple', 500, 'lr')**	0.7151	0.6509	0.6993	0.7316	0.6345	0.6910	0.0172	0.7151	0.0862	0.0242
**('behavior_simple', 500, 'svm')**	0.7147	0.6483	0.7031	0.7267	0.6297	0.6827	0.0198	0.7072	0.0855	0.0245
**('behavior_simple', 500, 'nb')**	0.7283	0.6551	0.7378	0.7191	0.6271	0.6553	0.0276	0.6926	0.0882	0.0373
**('behavior_simple', 500, 'rf')**	0.6940	0.6358	0.6592	0.7328	0.6279	0.6886	0.0128	0.7225	0.0891	0.0339
**('behavior_simple', 500, 'adaboost')**	0.7688	0.6743	0.8642	0.6923	0.6099	0.6965	0.0169	0.7231	0.0874	0.0267
**('behavior_simple', 500, 'nn')**	0.7577	0.6677	0.8290	0.6976	0.6131	0.6834	0.0147	0.6967	0.0946	0.0133
**('behavior_simple', 750, 'lr')**	0.7170	0.6542	0.6991	0.7358	0.6389	0.7095	0.0107	0.7245	0.0709	0.0151
**('behavior_simple', 750, 'svm')**	0.7224	0.6569	0.7127	0.7325	0.6380	0.6992	0.0150	0.7148	0.0786	0.0157
**('behavior_simple', 750, 'nb')**	0.7354	0.6624	0.7489	0.7224	0.6331	0.6638	0.0208	0.7009	0.0699	0.0372
**('behavior_simple', 750, 'rf')**	0.6862	0.6337	0.6393	0.7405	0.6317	0.6922	0.0108	0.7273	0.0733	0.0351
**('behavior_simple', 750, 'adaboost')**	0.7721	0.6788	0.8686	0.6950	0.6145	0.7080	0.0044	0.7262	0.0734	0.0182
**('behavior_simple', 750, 'nn')**	0.7599	0.6725	0.8271	0.7028	0.6201	0.6948	0.0049	0.7130	0.0799	0.0182
**('behavior_simple', 1000, 'lr')**	0.7197	0.6591	0.6987	0.7421	0.6457	0.7097	0.0075	0.7301	0.0571	0.0203
**('behavior_simple', 1000, 'svm')**	0.7098	0.6525	0.6782	0.7445	0.6438	0.7015	0.0078	0.7232	0.0603	0.0216
**('behavior_simple', 1000, 'nb')**	0.7408	0.6643	0.7655	0.7176	0.6300	0.6630	0.0183	0.7059	0.0569	0.0429
**('behavior_simple', 1000, 'rf')**	0.6950	0.6430	0.6493	0.7476	0.6408	0.7006	0.0058	0.7294	0.0568	0.0289
**('behavior_simple', 1000, 'adaboost')**	0.7700	0.6763	0.8646	0.6940	0.6125	0.7115	0.0055	0.7298	0.0568	0.0183

	Test Performance							CV Performance		Diff.
	f1 score	accuracy	recall	precision	BACC	AUC	AUC SD	AUC	SD AUC	Train-Test
**('behavior_simple', 1000, 'nn')**	0.7635	0.6782	0.8288	0.7077	0.6272	0.7021	0.0102	0.7174	0.0638	0.0153
**('behavior_simple', 1500, 'lr')**	0.7221	0.6616	0.7017	0.7436	0.6479	0.7139	0.0044	0.7235	0.0458	0.0097
**('behavior_simple', 1500, 'svm')**	0.7311	0.6654	0.7262	0.7362	0.6448	0.7011	0.0115	0.7150	0.0511	0.0138
**('behavior_simple', 1500, 'nb')**	0.7432	0.6640	0.7760	0.7131	0.6261	0.6657	0.0130	0.7005	0.0504	0.0347
**('behavior_simple', 1500, 'rf')**	0.6862	0.6358	0.6354	0.7458	0.6360	0.6963	0.0055	0.7185	0.0455	0.0223
**('behavior_simple', 1500, 'adaboost')**	0.7750	0.6824	0.8734	0.6966	0.6176	0.7171	0.0025	0.7257	0.0458	0.0085
**('behavior_simple', 1500, 'nn')**	0.7588	0.6695	0.8297	0.6990	0.6152	0.7073	0.0062	0.7188	0.0479	0.0115
**('behavior_simple', 2000, 'lr')**	0.7298	0.6635	0.7253	0.7343	0.6425	0.7165	0.0021	0.7279	0.0413	0.0113
**('behavior_simple', 2000, 'svm')**	0.6998	0.6449	0.6607	0.7439	0.6395	0.7022	0.0065	0.7192	0.0445	0.0170
**('behavior_simple', 2000, 'nb')**	0.7451	0.6668	0.7773	0.7154	0.6293	0.6656	0.0102	0.7041	0.0440	0.0385
**('behavior_simple', 2000, 'rf')**	0.6878	0.6337	0.6441	0.7379	0.6301	0.7002	0.0081	0.7218	0.0406	0.0216
**('behavior_simple', 2000, 'adaboost')**	0.7739	0.6796	0.8751	0.6937	0.6134	0.7175	0.0052	0.7287	0.0417	0.0112
**('behavior_simple', 2000, 'nn')**	0.7538	0.6689	0.8087	0.7058	0.6216	0.7021	0.0108	0.7222	0.0422	0.0201
**('behavior_simple', 2500, 'lr')**	0.7249	0.6598	0.7154	0.7347	0.6410	0.7156	0.0005	0.7362	0.0360	0.0206
**('behavior_simple', 2500, 'svm')**	0.7251	0.6566	0.7227	0.7275	0.6342	0.7041	0.0017	0.7260	0.0377	0.0219
**('behavior_simple', 2500, 'nb')**	0.7418	0.6635	0.7715	0.7143	0.6269	0.6686	0.0026	0.7091	0.0458	0.0404
**('behavior_simple', 2500, 'rf')**	0.6962	0.6434	0.6521	0.7467	0.6405	0.7050	0.0015	0.7252	0.0347	0.0202
**('behavior_simple', 2500, 'adaboost')**	0.7729	0.6803	0.8683	0.6964	0.6167	0.7210	0.0025	0.7371	0.0362	0.0162
**('behavior_simple', 2500, 'nn')**	0.7693	0.6817	0.8472	0.7046	0.6257	0.7124	0.0067	0.7306	0.0378	0.0182
**('behavior_simple', 3000, 'lr')**	0.7233	0.6585	0.7125	0.7344	0.6401	0.7173	0.0005	0.7383	0.0301	0.0210
**('behavior_simple', 3000, 'svm')**	0.7277	0.6662	0.7118	0.7443	0.6508	0.7052	0.0085	0.7295	0.0305	0.0243
**('behavior_simple', 3000, 'nb')**	0.7443	0.6658	0.7766	0.7147	0.6282	0.6718	0.0030	0.7125	0.0407	0.0407
**('behavior_simple', 3000, 'rf')**	0.6838	0.6361	0.6281	0.7504	0.6388	0.6969	0.0030	0.7284	0.0350	0.0315
**('behavior_simple', 3000, 'adaboost')**	0.7810	0.6886	0.8865	0.6980	0.6215	0.7187	0.0016	0.7393	0.0300	0.0206
**('behavior_simple', 3000, 'nn')**	0.7652	0.6776	0.8384	0.7037	0.6231	0.7117	0.0044	0.7334	0.0335	0.0217
**('behavior_simple', 3651, 'lr')**	0.7229	0.6557	0.7169	0.7291	0.6350	0.7165	0.0003	0.7355	0.0243	0.0190
**('behavior_simple', 3651, 'svm')**	0.7231	0.6644	0.6994	0.7484	0.6525	0.7059	0.0006	0.7277	0.0246	0.0218

	Test Performance							CV Performance		Diff.
	f1 score	accuracy	recall	precision	BACC	AUC	AUC SD	AUC	SD AUC	Train-Test
**('behavior_simple', 3651, 'nb')**	0.7469	0.6689	0.7795	0.7169	0.6315	0.6688	0.0006	0.7109	0.0251	0.0421
**('behavior_simple', 3651, 'rf')**	0.6978	0.6489	0.6470	0.7572	0.6495	0.7054	0.0030	0.7280	0.0227	0.0226
**('behavior_simple', 3651, 'adaboost')**	0.7784	0.6858	0.8806	0.6974	0.6198	0.7219	0.0004	0.7366	0.0249	0.0147
**('behavior_simple', 3651, 'nn')**	0.7648	0.6794	0.8319	0.7077	0.6278	0.7101	0.0042	0.7320	0.0262	0.0219
**('behavior_extended', 100, 'lr')**	0.7261	0.6509	0.7384	0.7141	0.6212	0.6557	0.0261	0.7239	0.1991	0.0682
**('behavior_extended', 100, 'svm')**	0.6994	0.6376	0.6727	0.7282	0.6257	0.6519	0.1160	0.7300	0.2120	0.0781
**('behavior_extended', 100, 'nb')**	0.6483	0.5765	0.6231	0.6757	0.5607	0.5957	0.0261	0.5744	0.2034	-0.0213
**('behavior_extended', 100, 'rf')**	0.7463	0.6650	0.7867	0.7100	0.6237	0.6800	0.0219	0.7378	0.2029	0.0578
**('behavior_extended', 100, 'adaboost')**	0.6824	0.6005	0.6849	0.6799	0.5720	0.5951	0.0492	0.6828	0.2070	0.0877
**('behavior_extended', 100, 'nn')**	0.6401	0.5915	0.5797	0.7145	0.5955	0.6541	0.0324	0.6473	0.2216	-0.0068
**('behavior_extended', 200, 'lr')**	0.7334	0.6648	0.7358	0.7310	0.6408	0.6885	0.0252	0.7303	0.1318	0.0418
**('behavior_extended', 200, 'svm')**	0.7004	0.6390	0.6736	0.7295	0.6273	0.6905	0.0114	0.7438	0.1345	0.0533
**('behavior_extended', 200, 'nb')**	0.7358	0.6399	0.8004	0.6809	0.5856	0.6045	0.0186	0.6300	0.1297	0.0255
**('behavior_extended', 200, 'rf')**	0.7264	0.6590	0.7227	0.7302	0.6374	0.6991	0.0195	0.7591	0.1352	0.0600
**('behavior_extended', 200, 'adaboost')**	0.7651	0.6627	0.8769	0.6786	0.5901	0.6710	0.0245	0.7388	0.1309	0.0678
**('behavior_extended', 200, 'nn')**	0.6796	0.6176	0.6472	0.7154	0.6076	0.6642	0.0294	0.6729	0.1499	0.0088
**('behavior_extended', 300, 'lr')**	0.7322	0.6684	0.7234	0.7412	0.6498	0.7157	0.0166	0.7440	0.1201	0.0284
**('behavior_extended', 300, 'svm')**	0.7019	0.6438	0.6694	0.7377	0.6351	0.6985	0.0137	0.7435	0.1186	0.0450
**('behavior_extended', 300, 'nb')**	0.7271	0.6334	0.7795	0.6813	0.5839	0.6173	0.0259	0.6242	0.1151	0.0069
**('behavior_extended', 300, 'rf')**	0.7247	0.6633	0.7072	0.7431	0.6485	0.7104	0.0220	0.7563	0.1285	0.0458
**('behavior_extended', 300, 'adaboost')**	0.7739	0.6774	0.8812	0.6899	0.6084	0.6888	0.0275	0.7414	0.1275	0.0527
**('behavior_extended', 300, 'nn')**	0.7055	0.6367	0.6945	0.7168	0.6171	0.6706	0.0333	0.7002	0.1263	0.0296
**('behavior_extended', 400, 'lr')**	0.7390	0.6793	0.7245	0.7541	0.6641	0.7308	0.0189	0.7575	0.1005	0.0267
**('behavior_extended', 400, 'svm')**	0.6982	0.6445	0.6563	0.7457	0.6404	0.7078	0.0157	0.7506	0.0943	0.0428
**('behavior_extended', 400, 'nb')**	0.6932	0.6111	0.7013	0.6853	0.5805	0.6292	0.0313	0.6419	0.0998	0.0127
**('behavior_extended', 400, 'rf')**	0.7244	0.6642	0.7044	0.7456	0.6505	0.7160	0.0184	0.7659	0.0956	0.0499
**('behavior_extended', 400, 'adaboost')**	0.7667	0.6733	0.8566	0.6938	0.6112	0.6903	0.0123	0.7454	0.0965	0.0551

	Test Performance							CV Performance		Diff.
	f1 score	accuracy	recall	precision	BACC	AUC	AUC SD	AUC	SD AUC	Train-Test
**('behavior_extended', 400, 'nn')**	0.7504	0.6661	0.8011	0.7057	0.6203	0.6948	0.0272	0.7123	0.1033	0.0175
**('behavior_extended', 500, 'lr')**	0.7423	0.6815	0.7321	0.7528	0.6644	0.7298	0.0223	0.7499	0.0858	0.0201
**('behavior_extended', 500, 'svm')**	0.7028	0.6488	0.6627	0.7481	0.6442	0.7122	0.0133	0.7421	0.0775	0.0299
**('behavior_extended', 500, 'nb')**	0.7016	0.6189	0.7151	0.6886	0.5863	0.6345	0.0356	0.6514	0.0905	0.0169
**('behavior_extended', 500, 'rf')**	0.7281	0.6665	0.7127	0.7442	0.6508	0.7198	0.0136	0.7605	0.0863	0.0407
**('behavior_extended', 500, 'adaboost')**	0.7752	0.6817	0.8760	0.6952	0.6158	0.7010	0.0152	0.7449	0.0836	0.0439
**('behavior_extended', 500, 'nn')**	0.7481	0.6581	0.8103	0.6948	0.6066	0.6844	0.0377	0.7140	0.0962	0.0296
**('behavior_extended', 750, 'lr')**	0.7438	0.6884	0.7218	0.7671	0.6770	0.7452	0.0090	0.7609	0.0594	0.0157
**('behavior_extended', 750, 'svm')**	0.7114	0.6596	0.6694	0.7589	0.6563	0.7256	0.0103	0.7558	0.0648	0.0302
**('behavior_extended', 750, 'nb')**	0.7396	0.6646	0.7603	0.7200	0.6321	0.6649	0.0164	0.6811	0.0691	0.0163
**('behavior_extended', 750, 'rf')**	0.7264	0.6711	0.6969	0.7586	0.6624	0.7331	0.0158	0.7651	0.0618	0.0320
**('behavior_extended', 750, 'adaboost')**	0.7786	0.6914	0.8664	0.7071	0.6321	0.7175	0.0126	0.7620	0.0614	0.0444
**('behavior_extended', 750, 'nn')**	0.7595	0.6897	0.7821	0.7383	0.6584	0.7277	0.0109	0.7271	0.0729	-0.0006
**('behavior_extended', 1000, 'lr')**	0.7426	0.6876	0.7192	0.7675	0.6768	0.7445	0.0079	0.7676	0.0506	0.0231
**('behavior_extended', 1000, 'svm')**	0.7080	0.6588	0.6603	0.7633	0.6583	0.7331	0.0112	0.7620	0.0488	0.0288
**('behavior_extended', 1000, 'nb')**	0.7566	0.6739	0.8092	0.7105	0.6280	0.6704	0.0278	0.6916	0.0593	0.0212
**('behavior_extended', 1000, 'rf')**	0.7295	0.6717	0.7066	0.7540	0.6599	0.7389	0.0111	0.7688	0.0415	0.0299
**('behavior_extended', 1000, 'adaboost')**	0.7778	0.6955	0.8507	0.7164	0.6429	0.7226	0.0095	0.7656	0.0532	0.0430
**('behavior_extended', 1000, 'nn')**	0.7755	0.6936	0.8445	0.7168	0.6424	0.7309	0.0072	0.7255	0.0574	-0.0054
**('behavior_extended', 1500, 'lr')**	0.7541	0.7031	0.7266	0.7838	0.6952	0.7594	0.0039	0.7678	0.0427	0.0084
**('behavior_extended', 1500, 'svm')**	0.7038	0.6594	0.6459	0.7731	0.6640	0.7425	0.0099	0.7599	0.0420	0.0174
**('behavior_extended', 1500, 'nb')**	0.7589	0.6744	0.8179	0.7079	0.6258	0.6601	0.0080	0.6853	0.0501	0.0252
**('behavior_extended', 1500, 'rf')**	0.7331	0.6807	0.7000	0.7696	0.6742	0.7488	0.0046	0.7671	0.0410	0.0183
**('behavior_extended', 1500, 'adaboost')**	0.7816	0.6955	0.8694	0.7098	0.6365	0.7380	0.0098	0.7646	0.0434	0.0266
**('behavior_extended', 1500, 'nn')**	0.7705	0.6941	0.8197	0.7270	0.6516	0.7248	0.0069	0.7290	0.0439	0.0043
**('behavior_extended', 2000, 'lr')**	0.7635	0.7133	0.7389	0.7899	0.7046	0.7670	0.0036	0.7745	0.0371	0.0075
**('behavior_extended', 2000, 'svm')**	0.7138	0.6700	0.6568	0.7817	0.6745	0.7470	0.0094	0.7646	0.0381	0.0176

	Test Performance							CV Performance		Diff.
	f1 score	accuracy	recall	precision	BACC	AUC	AUC SD	AUC	SD AUC	Train-Test
**('behavior_extended', 2000, 'nb')**	0.7666	0.6777	0.8450	0.7016	0.6210	0.6535	0.0078	0.6802	0.0399	0.0267
**('behavior_extended', 2000, 'rf')**	0.7410	0.6900	0.7079	0.7775	0.6840	0.7500	0.0055	0.7700	0.0368	0.0200
**('behavior_extended', 2000, 'adaboost')**	0.7901	0.7048	0.8869	0.7124	0.6431	0.7497	0.0043	0.7678	0.0404	0.0181
**('behavior_extended', 2000, 'nn')**	0.7717	0.7023	0.8031	0.7427	0.6682	0.7490	0.0147	0.7460	0.0395	-0.0030
**('behavior_extended', 2500, 'lr')**	0.7621	0.7127	0.7344	0.7920	0.7054	0.7693	0.0031	0.7815	0.0357	0.0123
**('behavior_extended', 2500, 'svm')**	0.7147	0.6767	0.6463	0.7993	0.6870	0.7568	0.0072	0.7721	0.0360	0.0154
**('behavior_extended', 2500, 'nb')**	0.7651	0.6785	0.8355	0.7056	0.6253	0.6577	0.0063	0.6933	0.0448	0.0355
**('behavior_extended', 2500, 'rf')**	0.7467	0.6949	0.7176	0.7782	0.6873	0.7552	0.0036	0.7769	0.0349	0.0218
**('behavior_extended', 2500, 'adaboost')**	0.7957	0.7127	0.8930	0.7175	0.6516	0.7542	0.0039	0.7702	0.0360	0.0160
**('behavior_extended', 2500, 'nn')**	0.7820	0.7155	0.8144	0.7520	0.6819	0.7619	0.0130	0.7532	0.0287	-0.0086
**('behavior_extended', 3000, 'lr')**	0.7649	0.7177	0.7329	0.7998	0.7126	0.7730	0.0026	0.7857	0.0266	0.0127
**('behavior_extended', 3000, 'svm')**	0.7194	0.6845	0.6456	0.8123	0.6976	0.7638	0.0024	0.7759	0.0270	0.0121
**('behavior_extended', 3000, 'nb')**	0.7610	0.6781	0.8180	0.7114	0.6306	0.6682	0.0114	0.7047	0.0393	0.0365
**('behavior_extended', 3000, 'rf')**	0.7549	0.7054	0.7242	0.7884	0.6991	0.7608	0.0018	0.7831	0.0279	0.0224
**('behavior_extended', 3000, 'adaboost')**	0.7943	0.7114	0.8894	0.7176	0.6510	0.7608	0.0044	0.7777	0.0261	0.0169
**('behavior_extended', 3000, 'nn')**	0.7758	0.7145	0.7882	0.7638	0.6896	0.7541	0.0134	0.7578	0.0315	0.0036
**('behavior_extended', 3651, 'lr')**	0.7643	0.7173	0.7314	0.8002	0.7125	0.7749	0.0001	0.7846	0.0269	0.0097
**('behavior_extended', 3651, 'svm')**	0.7264	0.6881	0.6608	0.8064	0.6973	0.7667	0.0000	0.7752	0.0269	0.0085
**('behavior_extended', 3651, 'nb')**	0.7672	0.6813	0.8384	0.7072	0.6280	0.6654	0.0000	0.6979	0.0318	0.0326
**('behavior_extended', 3651, 'rf')**	0.7574	0.7091	0.7249	0.7930	0.7037	0.7630	0.0013	0.7818	0.0239	0.0188
**('behavior_extended', 3651, 'adaboost')**	0.7911	0.7095	0.8777	0.7200	0.6525	0.7603	0.0017	0.7768	0.0254	0.0164
**('behavior_extended', 3651, 'nn')**	0.7755	0.7027	0.8195	0.7359	0.6631	0.7532	0.0121	0.7619	0.0248	0.0088
**('behavior_selected', 100, 'lr')**	0.6462	0.6390	0.5262	0.8371	0.6772	0.7334	0.0235	0.7575	0.1800	0.0241
**('behavior_selected', 100, 'svm')**	0.6586	0.6395	0.5550	0.8098	0.6682	0.7269	0.0253	0.7623	0.1823	0.0354
**('behavior_selected', 100, 'nb')**	0.7267	0.6540	0.7341	0.7195	0.6269	0.7399	0.0203	0.7045	0.1937	-0.0354
**('behavior_selected', 100, 'rf')**	0.7102	0.6677	0.6500	0.7828	0.6737	0.7609	0.0113	0.7953	0.1780	0.0345
**('behavior_selected', 100, 'adaboost')**	0.7413	0.6575	0.7834	0.7035	0.6148	0.7303	0.0280	0.7889	0.1454	0.0586

	Test Performance							CV Performance		Diff.
	f1 score	accuracy	recall	precision	BACC	AUC	AUC SD	AUC	SD AUC	Train-Test
**('behavior_selected', 100, 'nn')**	0.7299	0.6549	0.7443	0.7160	0.6245	0.7082	0.0398	0.7602	0.1706	0.0520
**('behavior_selected', 200, 'lr')**	0.6697	0.6514	0.5640	0.8242	0.6811	0.7515	0.0155	0.7897	0.1023	0.0382
**('behavior_selected', 200, 'svm')**	0.6517	0.6401	0.5373	0.8278	0.6749	0.7367	0.0222	0.7807	0.1097	0.0441
**('behavior_selected', 200, 'nb')**	0.7699	0.6741	0.8703	0.6903	0.6077	0.7475	0.0173	0.7724	0.1242	0.0249
**('behavior_selected', 200, 'rf')**	0.7171	0.6784	0.6504	0.7989	0.6879	0.7666	0.0131	0.8169	0.1142	0.0503
**('behavior_selected', 200, 'adaboost')**	0.7511	0.6721	0.7897	0.7161	0.6322	0.7440	0.0200	0.8044	0.1043	0.0604
**('behavior_selected', 200, 'nn')**	0.7390	0.6695	0.7467	0.7314	0.6433	0.7347	0.0250	0.7712	0.1210	0.0365
**('behavior_selected', 300, 'lr')**	0.6928	0.6661	0.6011	0.8176	0.6881	0.7598	0.0153	0.7923	0.1016	0.0325
**('behavior_selected', 300, 'svm')**	0.6780	0.6558	0.5784	0.8191	0.6820	0.7507	0.0155	0.7809	0.1114	0.0302
**('behavior_selected', 300, 'nb')**	0.7736	0.6732	0.8910	0.6835	0.5994	0.7481	0.0173	0.7816	0.1066	0.0335
**('behavior_selected', 300, 'rf')**	0.7043	0.6737	0.6201	0.8149	0.6919	0.7718	0.0121	0.8111	0.1004	0.0393
**('behavior_selected', 300, 'adaboost')**	0.7628	0.6874	0.8022	0.7271	0.6485	0.7574	0.0144	0.8107	0.0974	0.0533
**('behavior_selected', 300, 'nn')**	0.7572	0.6847	0.7847	0.7315	0.6508	0.7607	0.0113	0.7950	0.0994	0.0343
**('behavior_selected', 400, 'lr')**	0.7008	0.6741	0.6090	0.8251	0.6962	0.7654	0.0142	0.7949	0.0755	0.0296
**('behavior_selected', 400, 'svm')**	0.6845	0.6599	0.5889	0.8173	0.6840	0.7539	0.0207	0.7924	0.0833	0.0385
**('behavior_selected', 400, 'nb')**	0.7785	0.6684	0.9301	0.6694	0.5797	0.7512	0.0166	0.7816	0.0808	0.0304
**('behavior_selected', 400, 'rf')**	0.7114	0.6803	0.6290	0.8187	0.6977	0.7810	0.0133	0.8182	0.0829	0.0372
**('behavior_selected', 400, 'adaboost')**	0.7560	0.6882	0.7707	0.7418	0.6603	0.7678	0.0165	0.8053	0.0795	0.0374
**('behavior_selected', 400, 'nn')**	0.7566	0.6860	0.7788	0.7356	0.6546	0.7604	0.0180	0.7950	0.0786	0.0346
**('behavior_selected', 500, 'lr')**	0.6931	0.6710	0.5930	0.8339	0.6974	0.7651	0.0101	0.7886	0.0655	0.0235
**('behavior_selected', 500, 'svm')**	0.6726	0.6601	0.5574	0.8479	0.6948	0.7497	0.0154	0.7840	0.0694	0.0344
**('behavior_selected', 500, 'nb')**	0.7771	0.6670	0.9266	0.6692	0.5791	0.7495	0.0160	0.7785	0.0753	0.0289
**('behavior_selected', 500, 'rf')**	0.7089	0.6817	0.6186	0.8301	0.7031	0.7837	0.0099	0.8145	0.0663	0.0308
**('behavior_selected', 500, 'adaboost')**	0.7578	0.6911	0.7714	0.7447	0.6639	0.7724	0.0114	0.8101	0.0652	0.0377
**('behavior_selected', 500, 'nn')**	0.7573	0.6859	0.7821	0.7340	0.6533	0.7661	0.0112	0.7885	0.0671	0.0224
**('behavior_selected', 750, 'lr')**	0.6927	0.6744	0.5856	0.8477	0.7045	0.7716	0.0091	0.7979	0.0520	0.0263
**('behavior_selected', 750, 'svm')**	0.6962	0.6777	0.5895	0.8501	0.7076	0.7629	0.0110	0.7980	0.0527	0.0351

	Test Performance							CV Performance		Diff.
	f1 score	accuracy	recall	precision	BACC	AUC	AUC SD	AUC	SD AUC	Train-Test
**('behavior_selected', 750, 'nb')**	0.7733	0.6679	0.9044	0.6755	0.5877	0.7511	0.0126	0.7893	0.0568	0.0381
**('behavior_selected', 750, 'rf')**	0.7064	0.6821	0.6105	0.8381	0.7063	0.7885	0.0103	0.8175	0.0505	0.0290
**('behavior_selected', 750, 'adaboost')**	0.7693	0.7010	0.7956	0.7446	0.6689	0.7811	0.0066	0.8196	0.0555	0.0385
**('behavior_selected', 750, 'nn')**	0.7605	0.6930	0.7777	0.7439	0.6643	0.7693	0.0063	0.7968	0.0494	0.0274
**('behavior_selected', 1000, 'lr')**	0.6933	0.6741	0.5878	0.8449	0.7034	0.7728	0.0084	0.8030	0.0469	0.0302
**('behavior_selected', 1000, 'svm')**	0.6903	0.6755	0.5773	0.8584	0.7088	0.7686	0.0101	0.8022	0.0478	0.0337
**('behavior_selected', 1000, 'nb')**	0.7757	0.6684	0.9153	0.6731	0.5847	0.7498	0.0100	0.7917	0.0462	0.0419
**('behavior_selected', 1000, 'rf')**	0.7190	0.6936	0.6258	0.8449	0.7165	0.7952	0.0107	0.8208	0.0431	0.0256
**('behavior_selected', 1000, 'adaboost')**	0.7697	0.7045	0.7882	0.7521	0.6762	0.7873	0.0097	0.8204	0.0450	0.0331
**('behavior_selected', 1000, 'nn')**	0.7691	0.7018	0.7926	0.7469	0.6710	0.7767	0.0109	0.8049	0.0434	0.0282
**('behavior_selected', 1500, 'lr')**	0.6979	0.6807	0.5886	0.8570	0.7119	0.7756	0.0049	0.8032	0.0367	0.0276
**('behavior_selected', 1500, 'svm')**	0.6900	0.6774	0.5729	0.8672	0.7128	0.7731	0.0083	0.7996	0.0366	0.0265
**('behavior_selected', 1500, 'nb')**	0.7765	0.6668	0.9240	0.6696	0.5796	0.7490	0.0061	0.7873	0.0383	0.0383
**('behavior_selected', 1500, 'rf')**	0.7173	0.6886	0.6306	0.8318	0.7083	0.7979	0.0092	0.8214	0.0356	0.0235
**('behavior_selected', 1500, 'adaboost')**	0.7731	0.7086	0.7921	0.7549	0.6803	0.7922	0.0076	0.8190	0.0326	0.0268
**('behavior_selected', 1500, 'nn')**	0.7758	0.7119	0.7956	0.7570	0.6835	0.7822	0.0140	0.8038	0.0342	0.0216
**('behavior_selected', 2000, 'lr')**	0.6959	0.6793	0.5856	0.8574	0.7111	0.7753	0.0030	0.8029	0.0350	0.0276
**('behavior_selected', 2000, 'svm')**	0.6708	0.6681	0.5397	0.8860	0.7116	0.7796	0.0028	0.8020	0.0329	0.0224
**('behavior_selected', 2000, 'nb')**	0.7762	0.6648	0.9275	0.6673	0.5758	0.7488	0.0054	0.7853	0.0354	0.0366
**('behavior_selected', 2000, 'rf')**	0.7131	0.6892	0.6166	0.8455	0.7138	0.7998	0.0045	0.8199	0.0290	0.0201
**('behavior_selected', 2000, 'adaboost')**	0.7724	0.7067	0.7943	0.7517	0.6770	0.7916	0.0063	0.8183	0.0309	0.0267
**('behavior_selected', 2000, 'nn')**	0.7742	0.7059	0.8048	0.7459	0.6724	0.7833	0.0092	0.8082	0.0338	0.0248
**('behavior_selected', 2500, 'lr')**	0.7003	0.6799	0.5968	0.8471	0.7080	0.7770	0.0026	0.8087	0.0372	0.0317
**('behavior_selected', 2500, 'svm')**	0.6810	0.6762	0.5517	0.8897	0.7184	0.7827	0.0029	0.8088	0.0338	0.0261
**('behavior_selected', 2500, 'nb')**	0.7784	0.6671	0.9330	0.6677	0.5770	0.7492	0.0009	0.7915	0.0393	0.0423
**('behavior_selected', 2500, 'rf')**	0.7251	0.6995	0.6325	0.8495	0.7222	0.8019	0.0051	0.8242	0.0314	0.0223
**('behavior_selected', 2500, 'adaboost')**	0.7754	0.7132	0.7904	0.7610	0.6870	0.7977	0.0023	0.8217	0.0290	0.0241

	Test Performance							CV Performance		Diff.
	f1 score	accuracy	recall	precision	BACC	AUC	AUC SD	AUC	SD AUC	Train-Test
**('behavior_selected', 2500, 'nn')**	0.7772	0.7059	0.8188	0.7396	0.6676	0.7746	0.0135	0.8110	0.0374	0.0365
**('behavior_selected', 3000, 'lr')**	0.7017	0.6813	0.5983	0.8483	0.7094	0.7784	0.0025	0.8092	0.0293	0.0308
**('behavior_selected', 3000, 'svm')**	0.6652	0.6644	0.5320	0.8871	0.7092	0.7836	0.0033	0.8096	0.0258	0.0260
**('behavior_selected', 3000, 'nb')**	0.7796	0.6726	0.9243	0.6741	0.5873	0.7507	0.0020	0.7939	0.0319	0.0432
**('behavior_selected', 3000, 'rf')**	0.7240	0.6968	0.6346	0.8425	0.7178	0.8034	0.0028	0.8270	0.0266	0.0236
**('behavior_selected', 3000, 'adaboost')**	0.7796	0.7177	0.7969	0.7631	0.6909	0.8029	0.0020	0.8278	0.0254	0.0249
**('behavior_selected', 3000, 'nn')**	0.7815	0.7177	0.8057	0.7587	0.6879	0.7918	0.0018	0.8146	0.0291	0.0228
**('behavior_selected', 3651, 'lr')**	0.7018	0.6826	0.5961	0.8531	0.7120	0.7766	0.0001	0.8081	0.0214	0.0315
**('behavior_selected', 3651, 'svm')**	0.6510	0.6544	0.5146	0.8860	0.7017	0.7845	0.0002	0.8077	0.0193	0.0232
**('behavior_selected', 3651, 'nb')**	0.7809	0.6799	0.9105	0.6836	0.6018	0.7496	0.0002	0.7924	0.0264	0.0428
**('behavior_selected', 3651, 'rf')**	0.7297	0.7000	0.6463	0.8377	0.7181	0.8018	0.0007	0.8266	0.0227	0.0247
**('behavior_selected', 3651, 'adaboost')**	0.7795	0.7159	0.8013	0.7588	0.6870	0.8017	0.0008	0.8262	0.0234	0.0245
**('behavior_selected', 3651, 'nn')**	0.7719	0.7036	0.8006	0.7453	0.6707	0.7929	0.0019	0.8155	0.0211	0.0226
**('all_features', 100, 'lr')**	0.6771	0.6402	0.6022	0.7734	0.6531	0.7281	0.0243	0.7878	0.1608	0.0597
**('all_features', 100, 'svm')**	0.7091	0.6416	0.6974	0.7213	0.6227	0.6549	0.1108	0.7497	0.1989	0.0948
**('all_features', 100, 'nb')**	0.6888	0.6423	0.6319	0.7570	0.6458	0.7234	0.0293	0.7253	0.1829	0.0018
**('all_features', 100, 'rf')**	0.7414	0.6756	0.7421	0.7407	0.6531	0.7429	0.0236	0.8098	0.1770	0.0669
**('all_features', 100, 'adaboost')**	0.7289	0.6580	0.7338	0.7240	0.6323	0.7175	0.0191	0.7839	0.1628	0.0664
**('all_features', 100, 'nn')**	0.7307	0.6555	0.7459	0.7161	0.6249	0.6887	0.0233	0.7425	0.1859	0.0538
**('all_features', 200, 'lr')**	0.6836	0.6546	0.5956	0.8021	0.6746	0.7454	0.0186	0.7800	0.1175	0.0346
**('all_features', 200, 'svm')**	0.7003	0.6546	0.6441	0.7672	0.6581	0.7284	0.0218	0.7551	0.1185	0.0268
**('all_features', 200, 'nb')**	0.7366	0.6617	0.7548	0.7192	0.6301	0.7512	0.0173	0.7741	0.1259	0.0229
**('all_features', 200, 'rf')**	0.7304	0.6825	0.6865	0.7803	0.6811	0.7665	0.0151	0.8150	0.1081	0.0485
**('all_features', 200, 'adaboost')**	0.7337	0.6620	0.7432	0.7244	0.6344	0.7366	0.0252	0.7935	0.1145	0.0569
**('all_features', 200, 'nn')**	0.7231	0.6568	0.7153	0.7311	0.6369	0.7056	0.0351	0.7452	0.1224	0.0396
**('all_features', 300, 'lr')**	0.6964	0.6684	0.6070	0.8167	0.6892	0.7657	0.0138	0.7826	0.0987	0.0169
**('all_features', 300, 'svm')**	0.7158	0.6710	0.6611	0.7802	0.6743	0.7421	0.0164	0.7698	0.1098	0.0276

	Test Performance							CV Performance		Diff.
	f1 score	accuracy	recall	precision	BACC	AUC	AUC SD	AUC	SD AUC	Train-Test
**('all_features', 300, 'nb')**	0.7523	0.6683	0.8039	0.7069	0.6223	0.7585	0.0133	0.7814	0.0978	0.0229
**('all_features', 300, 'rf')**	0.7341	0.6900	0.6830	0.7935	0.6924	0.7767	0.0131	0.8172	0.0942	0.0405
**('all_features', 300, 'adaboost')**	0.7444	0.6780	0.7483	0.7405	0.6542	0.7545	0.0159	0.7970	0.0894	0.0424
**('all_features', 300, 'nn')**	0.7560	0.6884	0.7705	0.7420	0.6605	0.7495	0.0144	0.7685	0.1074	0.0190
**('all_features', 400, 'lr')**	0.7022	0.6717	0.6179	0.8132	0.6899	0.7700	0.0169	0.7879	0.0794	0.0179
**('all_features', 400, 'svm')**	0.7114	0.6748	0.6397	0.8012	0.6867	0.7553	0.0169	0.7798	0.0888	0.0244
**('all_features', 400, 'nb')**	0.7770	0.6787	0.8934	0.6874	0.6059	0.7640	0.0152	0.7901	0.0700	0.0261
**('all_features', 400, 'rf')**	0.7341	0.6934	0.6755	0.8038	0.6995	0.7834	0.0152	0.8229	0.0798	0.0395
**('all_features', 400, 'adaboost')**	0.7512	0.6856	0.7576	0.7450	0.6612	0.7613	0.0190	0.7970	0.0741	0.0356
**('all_features', 400, 'nn')**	0.7531	0.6777	0.7847	0.7240	0.6414	0.7452	0.0244	0.7750	0.0876	0.0298
**('all_features', 500, 'lr')**	0.7046	0.6766	0.6157	0.8236	0.6972	0.7735	0.0141	0.7887	0.0636	0.0152
**('all_features', 500, 'svm')**	0.7138	0.6785	0.6397	0.8072	0.6917	0.7627	0.0165	0.7885	0.0707	0.0258
**('all_features', 500, 'nb')**	0.7762	0.6762	0.8963	0.6845	0.6016	0.7682	0.0137	0.7925	0.0684	0.0243
**('all_features', 500, 'rf')**	0.7281	0.6911	0.6603	0.8116	0.7016	0.7866	0.0122	0.8171	0.0638	0.0304
**('all_features', 500, 'adaboost')**	0.7553	0.6944	0.7526	0.7579	0.6747	0.7689	0.0134	0.7999	0.0670	0.0310
**('all_features', 500, 'nn')**	0.7547	0.6715	0.8063	0.7092	0.6259	0.7437	0.0192	0.7806	0.0730	0.0368
**('all_features', 750, 'lr')**	0.7109	0.6837	0.6205	0.8320	0.7051	0.7840	0.0130	0.8046	0.0572	0.0206
**('all_features', 750, 'svm')**	0.7137	0.6834	0.6297	0.8235	0.7017	0.7763	0.0149	0.8085	0.0560	0.0321
**('all_features', 750, 'nb')**	0.7687	0.6755	0.8607	0.6945	0.6128	0.7743	0.0034	0.8020	0.0601	0.0277
**('all_features', 750, 'rf')**	0.7227	0.6911	0.6424	0.8259	0.7076	0.7890	0.0149	0.8269	0.0475	0.0379
**('all_features', 750, 'adaboost')**	0.7554	0.6933	0.7559	0.7549	0.6721	0.7777	0.0101	0.8116	0.0569	0.0339
**('all_features', 750, 'nn')**	0.7486	0.6810	0.7581	0.7394	0.6549	0.7589	0.0207	0.7971	0.0586	0.0382
**('all_features', 1000, 'lr')**	0.7082	0.6821	0.6157	0.8333	0.7046	0.7892	0.0089	0.8101	0.0474	0.0209
**('all_features', 1000, 'svm')**	0.7182	0.6908	0.6288	0.8372	0.7118	0.7786	0.0107	0.8122	0.0481	0.0336
**('all_features', 1000, 'nb')**	0.7726	0.6722	0.8886	0.6833	0.5989	0.7765	0.0068	0.8057	0.0468	0.0291
**('all_features', 1000, 'rf')**	0.7431	0.7094	0.6707	0.8330	0.7226	0.8023	0.0138	0.8288	0.0442	0.0266
**('all_features', 1000, 'adaboost')**	0.7627	0.7026	0.7629	0.7625	0.6822	0.7823	0.0186	0.8157	0.0472	0.0334

	Test Performance							CV Performance		Diff.
	f1 score	accuracy	recall	precision	BACC	AUC	AUC SD	AUC	SD AUC	Train-Test
**('all_features', 1000, 'nn')**	0.6637	0.6134	0.6087	0.7295	0.6150	0.7445	0.0284	0.8028	0.0423	0.0583
**('all_features', 1500, 'lr')**	0.7087	0.6824	0.6166	0.8330	0.7046	0.7920	0.0053	0.8116	0.0350	0.0196
**('all_features', 1500, 'svm')**	0.7067	0.6832	0.6092	0.8414	0.7082	0.7836	0.0093	0.8108	0.0371	0.0272
**('all_features', 1500, 'nb')**	0.7758	0.6736	0.9013	0.6810	0.5964	0.7783	0.0061	0.8069	0.0393	0.0286
**('all_features', 1500, 'rf')**	0.7416	0.7108	0.6624	0.8423	0.7272	0.8048	0.0062	0.8280	0.0332	0.0232
**('all_features', 1500, 'adaboost')**	0.7804	0.7193	0.7961	0.7653	0.6933	0.8025	0.0067	0.8242	0.0331	0.0217
**('all_features', 1500, 'nn')**	0.7585	0.6999	0.7524	0.7648	0.6821	0.7763	0.0103	0.7967	0.0350	0.0204
**('all_features', 2000, 'lr')**	0.7043	0.6788	0.6105	0.8321	0.7019	0.7936	0.0039	0.8135	0.0316	0.0199
**('all_features', 2000, 'svm')**	0.7054	0.6824	0.6070	0.8419	0.7079	0.7858	0.0101	0.8127	0.0356	0.0269
**('all_features', 2000, 'nb')**	0.7769	0.6736	0.9070	0.6794	0.5945	0.7788	0.0053	0.8080	0.0327	0.0291
**('all_features', 2000, 'rf')**	0.7444	0.7122	0.6690	0.8390	0.7268	0.8075	0.0060	0.8258	0.0337	0.0183
**('all_features', 2000, 'adaboost')**	0.7800	0.7187	0.7956	0.7649	0.6927	0.8058	0.0049	0.8196	0.0341	0.0139
**('all_features', 2000, 'nn')**	0.7547	0.6952	0.7485	0.7611	0.6772	0.7757	0.0137	0.7974	0.0362	0.0218
**('all_features', 2500, 'lr')**	0.7079	0.6831	0.6128	0.8378	0.7069	0.7971	0.0030	0.8192	0.0316	0.0221
**('all_features', 2500, 'svm')**	0.7064	0.6835	0.6077	0.8434	0.7092	0.7950	0.0082	0.8171	0.0363	0.0221
**('all_features', 2500, 'nb')**	0.7800	0.6762	0.9163	0.6791	0.5949	0.7806	0.0012	0.8138	0.0367	0.0332
**('all_features', 2500, 'rf')**	0.7451	0.7145	0.6659	0.8457	0.7310	0.8066	0.0027	0.8298	0.0329	0.0232
**('all_features', 2500, 'adaboost')**	0.7921	0.7351	0.8057	0.7790	0.7111	0.8133	0.0041	0.8259	0.0268	0.0126
**('all_features', 2500, 'nn')**	0.7648	0.7073	0.7598	0.7699	0.6894	0.7852	0.0139	0.8066	0.0251	0.0214
**('all_features', 3000, 'lr')**	0.7085	0.6826	0.6157	0.8343	0.7053	0.7999	0.0021	0.8203	0.0266	0.0204
**('all_features', 3000, 'svm')**	0.7114	0.6899	0.6099	0.8534	0.7170	0.7944	0.0035	0.8192	0.0284	0.0248
**('all_features', 3000, 'nb')**	0.7801	0.6799	0.9061	0.6848	0.6032	0.7806	0.0039	0.8154	0.0321	0.0347
**('all_features', 3000, 'rf')**	0.7405	0.7114	0.6572	0.8479	0.7297	0.8112	0.0011	0.8308	0.0255	0.0196
**('all_features', 3000, 'adaboost')**	0.7926	0.7337	0.8122	0.7739	0.7071	0.8141	0.0008	0.8321	0.0245	0.0181
**('all_features', 3000, 'nn')**	0.7722	0.7159	0.7686	0.7759	0.6981	0.7865	0.0064	0.8083	0.0297	0.0217
**('all_features', 3651, 'lr')**	0.7045	0.6817	0.6055	0.8421	0.7075	0.7986	0.0001	0.8170	0.0205	0.0185
**('all_features', 3651, 'svm')**	0.7034	0.6858	0.5946	0.8609	0.7167	0.7966	0.0020	0.8158	0.0212	0.0192

	Test Performance							CV Performance		Diff.
	f1 score	accuracy	recall	precision	BACC	AUC	AUC SD	AUC	SD AUC	Train-Test
**('all_features', 3651, 'nb')**	0.7790	0.6831	0.8916	0.6917	0.6124	0.7836	0.0000	0.8148	0.0275	0.0311
**('all_features', 3651, 'rf')**	0.7408	0.7109	0.6594	0.8451	0.7284	0.8078	0.0036	0.8278	0.0237	0.0200
**('all_features', 3651, 'adaboost')**	0.7878	0.7264	0.8108	0.7662	0.6978	0.8146	0.0020	0.8304	0.0201	0.0158
**('all_features', 3651, 'nn')**	0.7812	0.7246	0.7846	0.7778	0.7043	0.7974	0.0029	0.8087	0.0192	0.0113

Supplementary Table 3 – DeLong Test p-values

lr = Logistic Regression, rf = Random Forest, SVM = Support Vector Machines, NB = Naive Bayes, NN = Multilayer Percpetron Neural Network, and adaboost = adaBoost models, random = random chance

Simple Questionnaire

N	model	lr	rf	svm	nb	nn	adaboos	random
100	lr		0.48	0.45	0.93	0.60	0.81	0.34
100	rf	0.48		0.88	0.64	0.19	0.60	0.18
100	svm	0.45	0.88		0.53	0.24	0.60	0.06
100	nb	0.93	0.64	0.53		0.62	0.91	0.17
100	nn	0.60	0.19	0.24	0.62		0.46	0.98
100	adaboost	0.81	0.60	0.60	0.91	0.46		0.76
200	lr		0.38	0.29	0.64	0.63	0.73	0.19
200	rf	0.38		0.74	0.25	0.21	0.65	0.09
200	svm	0.29	0.74		0.19	0.18	0.52	**0.02**
200	nb	0.64	0.25	0.19		1.00	0.43	0.10
200	nn	0.63	0.21	0.18	1.00		0.49	0.12
200	adaboost	0.73	0.65	0.52	0.43	0.49		0.12
300	lr		0.27	0.72	0.39	0.73	0.81	**0.03**
300	rf	0.27		0.47	0.74	0.16	0.21	0.90
300	svm	0.72	0.47		0.69	0.54	0.59	**0.04**
300	nb	0.39	0.74	0.69		0.30	0.37	**0.03**
300	nn	0.73	0.16	0.54	0.30		1.00	0.18
300	adaboost	0.81	0.21	0.59	0.37	1.00		0.95
400	lr		0.32	**0.05**	0.62	0.74	0.26	**0.00**
400	rf	0.32		**0.02**	0.47	0.40	0.92	0.41
400	svm	**0.05**	**0.02**		**0.01**	**0.04**	**0.01**	**0.01**
400	nb	0.62	0.47	**0.01**		1.00	0.36	0.08
400	nn	0.74	0.40	**0.04**	1.00		0.43	0.14
400	adaboost	0.26	0.92	**0.01**	0.36	0.43		0.12
500	lr		0.53	0.23	0.57	0.71	0.40	**0.00**
500	rf	0.53		0.13	0.77	0.69	0.90	**0.00**
500	svm	0.23	0.13		0.10	0.16	0.10	**0.00**
500	nb	0.57	0.77	0.10		0.91	0.62	**0.00**
500	nn	0.71	0.69	0.16	0.91		0.60	**0.01**
500	adaboost	0.40	0.90	0.10	0.62	0.60		**0.05**
750	lr		0.28	0.12	0.70	0.74	0.13	0.20
750	rf	0.28		**0.03**	0.39	0.35	0.73	0.24
750	svm	0.12	**0.03**		0.08	0.07	**0.02**	**0.00**
750	nb	0.70	0.39	0.08		0.97	0.26	**0.02**
750	nn	0.74	0.35	0.07	0.97		0.24	**0.04**
750	adaboost	0.13	0.73	**0.02**	0.26	0.24		**0.00**
1000	lr		0.54	0.90	0.40	0.97	0.73	**0.02**
1000	rf	0.54		0.47	0.32	0.47	0.81	0.09
1000	svm	0.90	0.47		0.68	0.92	0.67	0.07
1000	nb	0.40	0.32	0.68		0.62	0.49	**0.03**
1000	nn	0.97	0.47	0.92	0.62		0.73	**0.00**
1000	adaboost	0.73	0.81	0.67	0.49	0.73		**0.02**
1500	lr		0.43	0.20	0.21	0.68	0.65	**0.04**
1500	rf	0.43		0.07	0.19	0.56	0.63	**0.01**
1500	svm	0.20	0.07		0.70	**0.05**	0.12	**0.01**
1500	nb	0.21	0.19	0.70		0.30	0.24	0.13
1500	nn	0.68	0.56	**0.05**	0.30		0.97	0.28
1500	adaboost	0.65	0.63	0.12	0.24	0.97		0.29
2000	lr		0.53	0.07	0.40	0.89	0.41	**0.00**

Extended Questionnaire

N	model	lr	rf	svm	nb	nn	adaboos	random
100	lr		0.16	**0.01**	0.09	0.69	0.09	**0.00**
100	rf	0.16		0.41	0.66	0.08	0.62	**0.00**
100	svm	**0.01**	0.41		0.77	**0.00**	0.72	**0.00**
100	nb	0.09	0.66	0.77		**0.05**	0.95	**0.00**
100	nn	0.69	0.08	**0.00**	**0.05**		**0.05**	**0.00**
100	adaboost	0.09	0.62	0.72	0.95	**0.05**		**0.00**
200	lr		**0.00**	**0.00**	**0.00**	**0.00**	**0.00**	**0.00**
200	rf	**0.00**		**0.00**	0.85	0.73	**0.00**	**0.00**
200	svm	**0.00**	**0.00**		**0.01**	**0.00**	0.98	**0.00**
200	nb	**0.00**	0.85	**0.01**		0.90	**0.00**	**0.00**
200	nn	**0.00**	0.73	**0.00**	0.90		**0.01**	**0.00**
200	adaboost	**0.00**	**0.00**	0.98	**0.00**	**0.01**		**0.00**
300	lr		**0.01**	**0.00**	**0.01**	**0.00**	**0.00**	**0.00**
300	rf	**0.01**		**0.05**	0.61	**0.01**	**0.00**	**0.00**
300	svm	**0.00**	**0.05**		0.19	0.52	**0.00**	**0.00**
300	nb	**0.01**	0.61	0.19		0.07	**0.00**	**0.00**
300	nn	**0.00**	**0.01**	0.52	0.07		**0.00**	**0.00**
300	adaboost	**0.00**	**0.00**	**0.00**	**0.00**	**0.00**		**0.00**
400	lr		**0.04**	**0.00**	**0.01**	**0.00**	**0.00**	**0.00**
400	rf	**0.04**		**0.02**	0.49	**0.00**	**0.00**	**0.00**
400	svm	**0.00**	**0.02**		0.08	0.14	**0.00**	**0.00**
400	nb	**0.01**	0.49	0.08		**0.01**	**0.00**	**0.00**
400	nn	**0.00**	**0.00**	0.14	**0.01**		**0.01**	**0.00**
400	adaboost	**0.00**	**0.00**	**0.00**	**0.00**	**0.01**		**0.00**
500	lr		**0.01**	**0.00**	**0.00**	**0.00**	**0.00**	**0.00**
500	rf	**0.01**		**0.01**	0.62	**0.00**	**0.00**	**0.00**
500	svm	**0.00**	**0.01**		**0.05**	**0.01**	**0.00**	**0.00**
500	nb	**0.00**	0.62	**0.05**		**0.00**	**0.00**	**0.00**
500	nn	**0.00**	**0.00**	**0.01**	**0.00**		0.58	**0.00**
500	adaboost	**0.00**	**0.00**	**0.00**	**0.00**	0.58		**0.00**
750	lr		0.06	**0.00**	**0.01**	**0.00**	**0.00**	**0.00**
750	rf	0.06		0.11	0.41	0.23	**0.00**	**0.00**
750	svm	**0.00**	0.11		0.39	0.84	**0.00**	**0.00**
750	nb	**0.01**	0.41	0.39		0.60	**0.00**	**0.00**
750	nn	**0.00**	0.23	0.84	0.60		**0.00**	**0.00**
750	adaboost	**0.00**	**0.00**	**0.00**	**0.00**	**0.00**		**0.00**
1000	lr		0.09	**0.00**	**0.01**	**0.00**	**0.00**	**0.00**
1000	rf	0.09		0.90	0.15	**0.00**	0.09	**0.00**
1000	svm	**0.00**	0.90		0.31	**0.00**	0.18	**0.00**
1000	nb	**0.01**	0.15	0.31		**0.00**	0.74	**0.00**
1000	nn	**0.00**	**0.00**	**0.00**	**0.00**		**0.00**	**0.00**
1000	adaboost	**0.00**	0.09	0.18	0.74	**0.00**		**0.00**
1500	lr		0.12	**0.00**	**0.01**	**0.00**	**0.00**	**0.00**
1500	rf	0.12		0.91	0.19	**0.00**	**0.02**	**0.00**
1500	svm	**0.00**	0.91		0.23	**0.00**	**0.02**	**0.00**
1500	nb	**0.01**	0.19	0.23		**0.02**	0.26	**0.00**
1500	nn	**0.00**	**0.00**	**0.00**	**0.02**		0.19	**0.00**
1500	adaboost	**0.00**	**0.02**	**0.02**	0.26	0.19		**0.00**
2000	lr		0.06	**0.00**	**0.00**	**0.00**	0.77	**0.00**

Simple Behavior

N	model	lr	rf	svm	nb	nn	adaboos	random
100	lr		0.36	**0.00**	**0.00**	**0.00**	**0.00**	**0.00**
100	rf	0.36		**0.03**	**0.00**	**0.00**	**0.00**	**0.00**
100	svm	**0.00**	**0.03**		**0.00**	0.42	0.59	**0.00**
100	nb	**0.00**	**0.00**	**0.00**		**0.00**	**0.00**	**0.00**
100	nn	**0.00**	**0.00**	0.42	**0.00**		0.80	**0.00**
100	adaboost	**0.00**	**0.00**	0.59	**0.00**	0.80		**0.00**
200	lr		0.14	0.90	**0.00**	**0.00**	0.12	**0.00**
200	rf	0.14		0.18	**0.00**	**0.00**	0.89	**0.00**
200	svm	0.90	0.18		**0.00**	**0.00**	0.29	**0.00**
200	nb	**0.00**	**0.00**	**0.00**		**0.00**	**0.00**	**0.00**
200	nn	**0.00**	**0.00**	**0.00**	**0.00**		**0.00**	**0.00**
200	adaboost	0.12	0.89	0.29	**0.00**	**0.00**		**0.00**
300	lr		0.43	**0.01**	**0.00**	**0.00**	**0.02**	**0.00**
300	rf	0.43		0.06	**0.00**	**0.02**	0.11	**0.00**
300	svm	**0.01**	0.06		**0.00**	0.57	0.98	**0.00**
300	nb	**0.00**	**0.00**	**0.00**		**0.00**	**0.00**	**0.00**
300	nn	**0.00**	**0.02**	0.57	**0.00**		0.68	**0.00**
300	adaboost	**0.02**	0.11	0.98	**0.00**	0.68		**0.00**
400	lr		0.19	**0.01**	**0.00**	**0.00**	0.19	**0.00**
400	rf	0.19		0.15	**0.00**	**0.02**	0.95	**0.00**
400	svm	**0.01**	0.15		**0.00**	0.44	0.24	**0.00**
400	nb	**0.00**	**0.00**	**0.00**		**0.00**	**0.00**	**0.00**
400	nn	**0.00**	**0.02**	0.44	**0.00**		**0.05**	**0.00**
400	adaboost	0.19	0.95	0.24	**0.00**	**0.05**		**0.00**
500	lr		0.43	**0.00**	**0.00**	**0.01**	0.13	**0.00**
500	rf	0.43		**0.00**	**0.00**	**0.04**	0.49	**0.00**
500	svm	**0.00**	**0.00**		**0.00**	**0.01**	**0.00**	**0.00**
500	nb	**0.00**	**0.00**	**0.00**		**0.00**	**0.00**	**0.00**
500	nn	**0.01**	**0.04**	**0.01**	**0.00**		0.26	**0.00**
500	adaboost	0.13	0.49	**0.00**	**0.00**	0.26		**0.00**
750	lr		**0.00**	**0.00**	**0.00**	**0.00**	**0.00**	**0.00**
750	rf	**0.00**		0.20	**0.00**	0.24	**0.05**	**0.00**
750	svm	**0.00**	0.20		**0.00**	**0.05**	**0.01**	**0.00**
750	nb	**0.00**	**0.00**	**0.00**		**0.00**	**0.00**	**0.00**
750	nn	**0.00**	0.24	**0.05**	**0.00**		0.44	**0.00**
750	adaboost	**0.00**	**0.05**	**0.01**	**0.00**	0.44		**0.00**
1000	lr		**0.03**	**0.00**	**0.00**	0.06	0.95	**0.00**
1000	rf	**0.03**		**0.00**	**0.00**	0.93	**0.03**	**0.00**
1000	svm	**0.00**	**0.00**		**0.00**	**0.00**	**0.00**	**0.00**
1000	nb	**0.00**	**0.00**	**0.00**		**0.00**	**0.00**	**0.00**
1000	nn	0.06	0.93	**0.00**	**0.00**		0.06	**0.00**
1000	adaboost	0.95	**0.03**	**0.00**	**0.00**	0.06		**0.00**
1500	lr		**0.00**	**0.00**	**0.00**	0.07	0.10	**0.00**
1500	rf	**0.00**		0.09	**0.00**	**0.00**	**0.01**	**0.00**
1500	svm	**0.00**	0.09		**0.00**	**0.00**	**0.00**	**0.00**
1500	nb	**0.00**	**0.00**	**0.00**		**0.00**	**0.00**	**0.00**
1500	nn	0.07	**0.00**	**0.00**	**0.00**		0.87	**0.00**
1500	adaboost	0.10	**0.01**	**0.00**	**0.00**	0.87		**0.00**
2000	lr		**0.00**	**0.00**	**0.00**	**0.00**	0.27	**0.00**

Simple Questionnaire

N	model	lr	rf	svm	nb	nn	adaboos	random
2000	rf	0.53		0.06	0.31	0.51	0.90	0.19
2000	svm	0.07	0.06		0.35	0.06	0.05	0.08
2000	nb	0.40	0.31	0.35		0.53	0.21	0.10
2000	nn	0.89	0.51	0.06	0.53		0.50	0.09
2000	adaboost	0.41	0.90	0.05	0.21	0.50		0.11
2500	lr		0.51	0.75	0.79	0.76	0.34	0.16
2500	rf	0.51		0.32	0.61	0.60	0.76	0.05
2500	svm	0.75	0.32		0.63	0.47	0.25	0.01
2500	nb	0.79	0.61	0.63		0.90	0.42	0.07
2500	nn	0.76	0.60	0.47	0.90		0.48	0.10
2500	adaboost	0.34	0.76	0.25	0.42	0.48		0.87
3000	lr		0.21	0.94	0.78	0.36	0.13	0.01
3000	rf	0.21		0.12	0.29	0.42	0.89	0.17
3000	svm	0.94	0.12		0.79	0.28	0.14	0.02
3000	nb	0.78	0.29	0.79		0.59	0.17	0.13
3000	nn	0.36	0.42	0.28	0.59		0.37	0.05
3000	adaboost	0.13	0.89	0.14	0.17	0.37		0.30
3651	lr		0.11	0.99	0.86	0.86	0.92	0.05
3651	rf	0.11		0.06	0.14	0.05	0.14	0.43
3651	svm	0.99	0.06		0.89	0.85	0.90	0.08
3651	nb	0.86	0.14	0.89		0.80	0.99	0.02
3651	nn	0.86	0.05	0.85	0.80		0.82	0.08
3651	adaboost	0.92	0.14	0.90	0.99	0.82		0.18

Extended Questionnaire

N	model	lr	rf	svm	nb	nn	adaboos	random
2000	rf	0.06		0.67	0.18	0.41	0.11	0.00
2000	svm	0.00	0.67		0.13	0.14	0.25	0.00
2000	nb	0.00	0.18	0.13		0.83	0.02	0.00
2000	nn	0.00	0.41	0.14	0.83		0.04	0.00
2000	adaboost	0.77	0.11	0.25	0.02	0.04		0.00
2500	lr		0.07	0.00	0.01	0.00	0.51	0.00
2500	rf	0.07		0.53	0.22	0.22	0.23	0.00
2500	svm	0.00	0.53		0.13	0.03	0.61	0.00
2500	nb	0.01	0.22	0.13		0.75	0.06	0.00
2500	nn	0.00	0.22	0.03	0.75		0.04	0.00
2500	adaboost	0.51	0.23	0.61	0.06	0.04		0.00
3000	lr		0.06	0.08	0.02	0.06	0.74	0.00
3000	rf	0.06		0.33	0.21	1.00	0.16	0.00
3000	svm	0.08	0.33		0.08	0.35	0.61	0.00
3000	nb	0.02	0.21	0.08		0.41	0.04	0.00
3000	nn	0.06	1.00	0.35	0.41		0.27	0.00
3000	adaboost	0.74	0.16	0.61	0.04	0.27		0.00
3651	lr		0.17	0.00	0.01	0.00	0.93	0.00
3651	rf	0.17		0.63	0.07	0.14	0.16	0.00
3651	svm	0.00	0.63		0.08	0.02	0.35	0.00
3651	nb	0.01	0.07	0.08		0.90	0.02	0.00
3651	nn	0.00	0.14	0.02	0.90		0.01	0.00
3651	adaboost	0.93	0.16	0.35	0.02	0.01		0.00

Simple Behavior

N	model	lr	rf	svm	nb	nn	adaboos	random
2000	rf	0.00		0.00	0.00	0.54	0.00	0.00
2000	svm	0.00	0.00		0.06	0.00	0.00	0.00
2000	nb	0.00	0.00	0.06		0.00	0.00	0.00
2000	nn	0.00	0.54	0.00	0.00		0.00	0.00
2000	adaboost	0.27	0.00	0.00	0.00	0.00		0.00
2500	lr		0.01	0.00	0.00	0.50	0.38	0.00
2500	rf	0.01		0.00	0.00	0.05	0.01	0.00
2500	svm	0.00	0.00		0.32	0.00	0.00	0.00
2500	nb	0.00	0.00	0.32		0.00	0.00	0.00
2500	nn	0.50	0.05	0.00	0.00		0.26	0.00
2500	adaboost	0.38	0.01	0.00	0.00	0.26		0.00
3000	lr		0.00	0.00	0.00	0.10	0.38	0.00
3000	rf	0.00		0.50	0.00	0.00	0.00	0.00
3000	svm	0.00	0.50		0.01	0.01	0.00	0.00
3000	nb	0.00	0.00	0.01		0.00	0.00	0.00
3000	nn	0.10	0.00	0.01	0.00		0.06	0.00
3000	adaboost	0.38	0.00	0.00	0.00	0.06		0.00
3651	lr		0.01	0.00	0.00	0.12	0.13	0.00
3651	rf	0.01		0.84	0.00	0.23	0.00	0.00
3651	svm	0.00	0.84		0.00	0.29	0.00	0.00
3651	nb	0.00	0.00	0.00		0.00	0.00	0.00
3651	nn	0.12	0.23	0.29	0.00		0.03	0.00
3651	adaboost	0.13	0.00	0.00	0.00	0.03		0.00

Extended Behavior

N	model	lr	rf	svm	nb	nn	adaboos	random
100	lr		0.00	0.62	0.00	0.13	0.00	0.00
100	rf	0.00		0.00	0.00	0.00	0.00	0.00
100	svm	0.62	0.00		0.00	0.32	0.00	0.00
100	nb	0.00	0.00	0.00		0.00	0.29	0.00
100	nn	0.13	0.00	0.32	0.00		0.00	0.00
100	adaboost	0.00	0.00	0.00	0.29	0.00		0.00
200	lr		0.02	0.98	0.00	0.00	0.00	0.00
200	rf	0.02		0.01	0.00	0.00	0.00	0.00
200	svm	0.98	0.01		0.00	0.00	0.00	0.00
200	nb	0.00	0.00	0.00		0.00	0.00	0.00
200	nn	0.00	0.00	0.00	0.00		0.16	0.00
200	adaboost	0.00	0.00	0.00	0.00	0.16		0.00
300	lr		0.54	0.00	0.00	0.00	0.00	0.00
300	rf	0.54		0.00	0.00	0.00	0.00	0.00
300	svm	0.00	0.00		0.00	0.00	0.00	0.00
300	nb	0.00	0.00	0.00		0.00	0.00	0.00
300	nn	0.00	0.00	0.00	0.00		0.85	0.00
300	adaboost	0.00	0.00	0.00	0.00	0.85		0.00
400	lr		0.00	0.00	0.00	0.00	0.00	0.00
400	rf	0.00		0.00	0.00	0.00	0.00	0.00
400	svm	0.00	0.00		0.00	0.01	0.00	0.00
400	nb	0.00	0.00	0.00		0.00	0.00	0.00
400	nn	0.00	0.00	0.01	0.00		0.00	0.00
400	adaboost	0.00	0.00	0.00	0.00	0.00		0.00
500	lr		0.01	0.00	0.00	0.00	0.00	0.00
500	rf	0.01		0.00	0.00	0.00	0.00	0.00

Selected Behavior

N	model	lr	rf	svm	nb	nn	adaboos	random
100	lr		0.00	0.00	0.00	0.00	0.00	0.00
100	rf	0.00		0.00	0.00	0.00	0.00	0.00
100	svm	0.00	0.00		0.14	0.28	0.99	0.00
100	nb	0.00	0.00	0.14		0.03	0.13	0.00
100	nn	0.00	0.00	0.28	0.03		0.38	0.00
100	adaboost	0.00	0.00	0.99	0.13	0.38		0.00
200	lr		0.06	0.00	0.00	0.00	0.00	0.00
200	rf	0.06		0.00	0.00	0.00	0.00	0.00
200	svm	0.00	0.00		0.07	0.09	0.49	0.00
200	nb	0.00	0.00	0.07		0.55	0.22	0.00
200	nn	0.00	0.00	0.09	0.55		0.52	0.00
200	adaboost	0.00	0.00	0.49	0.22	0.52		0.00
300	lr		0.00	0.00	0.00	0.99	0.05	0.00
300	rf	0.00		0.00	0.00	0.01	0.00	0.00
300	svm	0.00	0.00		0.00	0.01	0.85	0.00
300	nb	0.00	0.00	0.00		0.00	0.00	0.00
300	nn	0.99	0.01	0.01	0.00		0.09	0.00
300	adaboost	0.05	0.00	0.85	0.00	0.09		0.00
400	lr		0.00	0.00	0.00	0.05	0.59	0.00
400	rf	0.00		0.00	0.00	0.00	0.00	0.00
400	svm	0.00	0.00		0.00	0.02	0.00	0.00
400	nb	0.00	0.00	0.00		0.00	0.00	0.00
400	nn	0.05	0.00	0.02	0.00		0.07	0.00
400	adaboost	0.59	0.00	0.00	0.00	0.07		0.00
500	lr		0.00	0.00	0.00	0.84	0.20	0.00
500	rf	0.00		0.00	0.00	0.00	0.00	0.00

Mixed Features

N	model	lr	rf	svm	nb	nn	adaboos	random
100	lr		0.53	0.00	0.00	0.00	0.00	0.00
100	rf	0.53		0.00	0.00	0.00	0.00	0.00
100	svm	0.00	0.00		0.00	0.01	0.00	0.00
100	nb	0.00	0.00	0.00		0.19	0.11	0.00
100	nn	0.00	0.00	0.01	0.19		0.00	0.00
100	adaboost	0.00	0.00	0.00	0.11	0.00		0.00
200	lr		0.00	0.00	0.01	0.00	0.26	0.00
200	rf	0.00		0.00	0.00	0.00	0.00	0.00
200	svm	0.00	0.00		0.95	0.00	0.06	0.00
200	nb	0.01	0.00	0.95		0.00	0.08	0.00
200	nn	0.00	0.00	0.00	0.00		0.00	0.00
200	adaboost	0.26	0.00	0.06	0.08	0.00		0.00
300	lr		0.01	0.00	0.00	0.00	0.01	0.00
300	rf	0.01		0.00	0.00	0.00	0.00	0.00
300	svm	0.00	0.00		0.36	0.04	0.00	0.00
300	nb	0.00	0.00	0.36		0.02	0.00	0.00
300	nn	0.00	0.00	0.04	0.02		0.19	0.00
300	adaboost	0.01	0.00	0.00	0.00	0.19		0.00
400	lr		0.00	0.00	0.00	0.00	0.01	0.00
400	rf	0.00		0.00	0.00	0.00	0.00	0.00
400	svm	0.00	0.00		0.10	0.00	0.58	0.00
400	nb	0.00	0.00	0.10		0.10	0.08	0.00
400	nn	0.00	0.00	0.00	0.10		0.00	0.00
400	adaboost	0.01	0.00	0.58	0.08	0.00		0.00
500	lr		0.00	0.00	0.00	0.00	0.06	0.00
500	rf	0.00		0.00	0.00	0.00	0.00	0.00

Extended Behavior

N	model	lr	rf	svm	nb	nn	adaboos	random
500	svm	0.00	0.00		0.00	0.00	0.00	0.00
500	nb	0.00	0.00	0.00		0.00	0.00	0.00
500	nn	0.00	0.00	0.00	0.00		0.61	0.00
500	adaboost	0.00	0.00	0.00	0.00	0.61		0.00
750	lr		0.01	0.00	0.00	0.00	0.00	0.00
750	rf	0.01		0.02	0.00	0.33	0.00	0.00
750	svm	0.00	0.02		0.00	0.59	0.00	0.00
750	nb	0.00	0.00	0.00		0.00	0.00	0.00
750	nn	0.00	0.33	0.59	0.00		0.00	0.00
750	adaboost	0.00	0.00	0.00	0.00	0.00		0.00
1000	lr		0.20	0.00	0.00	0.03	0.00	0.00
1000	rf	0.20		0.03	0.00	0.30	0.00	0.00
1000	svm	0.00	0.03		0.00	0.84	0.00	0.00
1000	nb	0.00	0.00	0.00		0.00	0.00	0.00
1000	nn	0.03	0.30	0.84	0.00		0.00	0.00
1000	adaboost	0.00	0.00	0.00	0.00	0.00		0.00
1500	lr		0.03	0.00	0.00	0.00	0.00	0.00
1500	rf	0.03		0.01	0.00	0.00	0.00	0.00
1500	svm	0.00	0.01		0.00	0.01	0.00	0.00
1500	nb	0.00	0.00	0.00		0.00	0.00	0.00
1500	nn	0.00	0.00	0.01	0.00		0.37	0.00
1500	adaboost	0.00	0.00	0.00	0.00	0.37		0.00
2000	lr		0.00	0.00	0.00	0.00	0.00	0.00
2000	rf	0.00		0.34	0.00	0.87	0.00	0.00
2000	svm	0.00	0.34		0.00	0.59	0.00	0.00
2000	nb	0.00	0.00	0.00		0.00	0.00	0.00
2000	nn	0.00	0.87	0.59	0.00		0.00	0.00
2000	adaboost	0.00	0.00	0.00	0.00	0.00		0.00
2500	lr		0.00	0.00	0.00	0.24	0.00	0.00
2500	rf	0.00		0.89	0.00	0.36	0.84	0.00
2500	svm	0.00	0.89		0.00	0.38	0.79	0.00
2500	nb	0.00	0.00	0.00		0.00	0.00	0.00
2500	nn	0.24	0.36	0.38	0.00		0.32	0.00
2500	adaboost	0.00	0.84	0.79	0.00	0.32		0.00
3000	lr		0.03	0.06	0.00	0.00	0.01	0.00
3000	rf	0.03		0.56	0.00	0.35	0.88	0.00
3000	svm	0.06	0.56		0.00	0.15	0.54	0.00
3000	nb	0.00	0.00	0.00		0.00	0.00	0.00
3000	nn	0.00	0.35	0.15	0.00		0.41	0.00
3000	adaboost	0.01	0.88	0.54	0.00	0.41		0.00
3651	lr		0.01	0.08	0.00	0.00	0.00	0.00
3651	rf	0.01		0.41	0.00	0.16	0.06	0.00
3651	svm	0.08	0.41		0.00	0.03	0.03	0.00
3651	nb	0.00	0.00	0.00		0.00	0.00	0.00
3651	nn	0.00	0.16	0.03	0.00		0.83	0.00
3651	adaboost	0.00	0.06	0.03	0.00	0.83		0.00

Selected Behavior

N	model	lr	rf	svm	nb	nn	adaboos	random
500	svm	0.00	0.00		0.02	0.00	0.00	0.00
500	nb	0.00	0.00	0.02		0.00	0.00	0.00
500	nn	0.84	0.00	0.00	0.00		0.32	0.00
500	adaboost	0.20	0.00	0.00	0.00	0.32		0.00
750	lr		0.00	0.01	0.00	0.24	0.10	0.00
750	rf	0.00		0.00	0.00	0.00	0.05	0.00
750	svm	0.01	0.00		0.00	0.34	0.00	0.00
750	nb	0.00	0.00	0.00		0.00	0.00	0.00
750	nn	0.24	0.00	0.34	0.00		0.02	0.00
750	adaboost	0.10	0.05	0.00	0.00	0.02		0.00
1000	lr		0.00	0.15	0.00	0.31	0.00	0.00
1000	rf	0.00		0.00	0.00	0.00	0.06	0.00
1000	svm	0.15	0.00		0.00	0.04	0.00	0.00
1000	nb	0.00	0.00	0.00		0.00	0.00	0.00
1000	nn	0.31	0.00	0.04	0.00		0.03	0.00
1000	adaboost	0.00	0.06	0.00	0.00	0.03		0.00
1500	lr		0.00	0.34	0.00	0.06	0.00	0.00
1500	rf	0.00		0.00	0.00	0.00	0.17	0.00
1500	svm	0.34	0.00		0.00	0.01	0.00	0.00
1500	nb	0.00	0.00	0.00		0.00	0.00	0.00
1500	nn	0.06	0.00	0.01	0.00		0.06	0.00
1500	adaboost	0.00	0.17	0.00	0.00	0.06		0.00
2000	lr		0.00	0.15	0.00	0.04	0.00	0.00
2000	rf	0.00		0.00	0.00	0.00	0.01	0.00
2000	svm	0.15	0.00		0.00	0.44	0.02	0.00
2000	nb	0.00	0.00	0.00		0.00	0.00	0.00
2000	nn	0.04	0.00	0.44	0.00		0.08	0.00
2000	adaboost	0.00	0.01	0.02	0.00	0.08		0.00
2500	lr		0.00	0.11	0.00	0.01	0.00	0.00
2500	rf	0.00		0.00	0.00	0.00	0.41	0.00
2500	svm	0.11	0.00		0.00	0.00	0.01	0.00
2500	nb	0.00	0.00	0.00		0.01	0.00	0.00
2500	nn	0.01	0.00	0.00	0.01		0.00	0.00
2500	adaboost	0.00	0.41	0.01	0.00	0.00		0.00
3000	lr		0.00	0.14	0.00	0.01	0.00	0.00
3000	rf	0.00		0.00	0.00	0.02	0.94	0.00
3000	svm	0.14	0.00		0.00	0.12	0.00	0.00
3000	nb	0.00	0.00	0.00		0.00	0.00	0.00
3000	nn	0.01	0.02	0.12	0.00		0.05	0.00
3000	adaboost	0.00	0.94	0.00	0.00	0.05		0.00
3651	lr		0.00	0.03	0.00	0.01	0.00	0.00
3651	rf	0.00		0.00	0.00	0.05	0.85	0.00
3651	svm	0.03	0.00		0.00	0.17	0.00	0.00
3651	nb	0.00	0.00	0.00		0.00	0.00	0.00
3651	nn	0.01	0.05	0.17	0.00		0.16	0.00
3651	adaboost	0.00	0.85	0.00	0.00	0.16		0.00

Mixed Features

N	model	lr	rf	svm	nb	nn	adaboos	random
500	svm	0.00	0.00		0.00	0.00	0.59	0.00
500	nb	0.00	0.00	0.00		0.00	0.00	0.00
500	nn	0.00	0.00	0.00	0.00		0.00	0.00
500	adaboost	0.06	0.00	0.59	0.00	0.00		0.00
750	lr		0.23	0.04	0.00	0.00	0.24	0.00
750	rf	0.23		0.00	0.00	0.00	0.02	0.00
750	svm	0.04	0.00		0.00	0.00	0.83	0.00
750	nb	0.00	0.00	0.00		0.12	0.01	0.00
750	nn	0.00	0.00	0.00	0.12		0.00	0.00
750	adaboost	0.24	0.02	0.83	0.01	0.00		0.00
1000	lr		0.01	0.00	0.00	0.00	0.34	0.00
1000	rf	0.01		0.00	0.00	0.00	0.00	0.00
1000	svm	0.00	0.00		0.00	0.00	0.32	0.00
1000	nb	0.00	0.00	0.00		0.00	0.00	0.00
1000	nn	0.00	0.00	0.00	0.00		0.00	0.00
1000	adaboost	0.34	0.00	0.32	0.00	0.00		0.00
1500	lr		0.00	0.01	0.00	0.00	0.04	0.00
1500	rf	0.00		0.00	0.00	0.00	0.58	0.00
1500	svm	0.01	0.00		0.00	0.07	0.00	0.00
1500	nb	0.00	0.00	0.00		0.06	0.00	0.00
1500	nn	0.00	0.00	0.07	0.06		0.00	0.00
1500	adaboost	0.04	0.58	0.00	0.00	0.00		0.00
2000	lr		0.00	0.01	0.00	0.00	0.01	0.00
2000	rf	0.00		0.00	0.00	0.00	0.82	0.00
2000	svm	0.01	0.00		0.00	0.01	0.00	0.00
2000	nb	0.00	0.00	0.00		0.05	0.00	0.00
2000	nn	0.00	0.00	0.01	0.05		0.00	0.00
2000	adaboost	0.01	0.82	0.00	0.00	0.00		0.00
2500	lr		0.06	0.59	0.00	0.03	0.00	0.00
2500	rf	0.06		0.02	0.00	0.00	0.13	0.00
2500	svm	0.59	0.02		0.00	0.06	0.00	0.00
2500	nb	0.00	0.00	0.00		0.04	0.00	0.00
2500	nn	0.03	0.00	0.06	0.04		0.00	0.00
2500	adaboost	0.00	0.13	0.00	0.00	0.00		0.00
3000	lr		0.02	0.19	0.00	0.00	0.02	0.00
3000	rf	0.02		0.00	0.00	0.00	0.57	0.00
3000	svm	0.19	0.00		0.00	0.00	0.00	0.00
3000	nb	0.00	0.00	0.00		0.24	0.00	0.00
3000	nn	0.00	0.00	0.00	0.24		0.00	0.00
3000	adaboost	0.02	0.57	0.00	0.00	0.00		0.00
3651	lr		0.08	0.58	0.00	0.70	0.01	0.00
3651	rf	0.08		0.03	0.00	0.06	0.15	0.00
3651	svm	0.58	0.03		0.00	0.99	0.00	0.00
3651	nb	0.00	0.00	0.00		0.00	0.00	0.00
3651	nn	0.70	0.06	0.99	0.00		0.01	0.00
3651	adaboost	0.01	0.15	0.00	0.00	0.01		0.00

Supplementary Table 4 – Overfitting Distribution

Mean test score minus training CV score

N=100		wcs_only	baseline_extended	behavior_simple	behavior_extended	behavior_selected	all_features
**Model**	**Measure**						
lr	**mean (SD)**	0.58 (0.07)	0.63 (0.11)	0.69 (0.11)	0.72 (0.08)	0.76 (0.08)	0.79 (0.04)
	**min/max**	0.5/0.69	0.46/0.76	0.47/0.86	0.58/0.85	0.62/0.85	0.73/0.86
	**% +0.05 test**	0.50	0.50	0.40	0.70	0.60	0.60
	**% +0.10 test**	0.40	0.50	0.40	0.30	0.20	0.20
	**% -0.05 test**	0.00	0.20	0.10	0.10	0.20	0.00
	**% -0.10 test**	0.00	0.00	0.10	0.00	0.10	0.00
svm	**mean (SD)**	0.6 (0.06)	0.64 (0.09)	0.73 (0.1)	0.73 (0.06)	0.76 (0.07)	0.75 (0.06)
	**min/max**	0.51/0.68	0.49/0.78	0.58/0.88	0.65/0.82	0.66/0.87	0.66/0.86
	**% +0.05 test**	0.70	0.70	0.60	0.60	0.60	0.70
	**% +0.10 test**	0.40	0.60	0.40	0.30	0.10	0.50
	**% -0.05 test**	0.00	0.00	0.10	0.00	0.20	0.00
	**% -0.10 test**	0.00	0.00	0.00	0.00	0.00	0.00
nb	**mean (SD)**	0.56 (0.08)	0.59 (0.07)	0.67 (0.12)	0.57 (0.05)	0.7 (0.08)	0.73 (0.05)
	**min/max**	0.43/0.71	0.49/0.73	0.46/0.84	0.51/0.65	0.56/0.81	0.65/0.83
	**% +0.05 test**	0.60	0.30	0.40	0.20	0.20	0.20
	**% +0.10 test**	0.30	0.10	0.30	0.00	0.00	0.10
	**% -0.05 test**	0.10	0.10	0.20	0.40	0.40	0.20
	**% -0.10 test**	0.00	0.00	0.20	0.00	0.20	0.00
rf	**mean (SD)**	0.63 (0.07)	0.67 (0.05)	0.74 (0.08)	0.74 (0.07)	0.8 (0.07)	0.81 (0.05)
	**min/max**	0.52/0.75	0.59/0.74	0.59/0.87	0.62/0.83	0.68/0.87	0.7/0.87
	**% +0.05 test**	0.70	0.90	0.70	0.60	0.60	0.70
	**% +0.10 test**	0.70	0.60	0.50	0.50	0.10	0.30
	**% -0.05 test**	0.00	0.00	0.10	0.10	0.10	0.00
	**% -0.10 test**	0.00	0.00	0.00	0.00	0.00	0.00
adaboost	**mean (SD)**	0.61 (0.09)	0.65 (0.07)	0.69 (0.11)	0.68 (0.07)	0.79 (0.07)	0.78 (0.05)
	**min/max**	0.4/0.72	0.54/0.74	0.51/0.87	0.58/0.77	0.66/0.88	0.68/0.88
	**% +0.05 test**	0.70	0.80	0.40	0.60	0.60	0.70
	**% +0.10 test**	0.60	0.50	0.40	0.50	0.30	0.20
	**% -0.05 test**	0.10	0.00	0.10	0.00	0.10	0.00
	**% -0.10 test**	0.10	0.00	0.10	0.00	0.00	0.00
nn	**mean (SD)**	0.6 (0.07)	0.64 (0.08)	0.68 (0.11)	0.65 (0.05)	0.76 (0.04)	0.74 (0.07)
	**min/max**	0.47/0.74	0.52/0.76	0.49/0.89	0.58/0.76	0.69/0.82	0.62/0.86
	**% +0.05 test**	0.80	0.60	0.60	0.10	0.60	0.60
	**% +0.10 test**	0.40	0.50	0.20	0.10	0.10	0.20
	**% -0.05 test**	0.00	0.00	0.20	0.20	0.00	0.20
	**% -0.10 test**	0.00	0.00	0.10	0.00	0.00	0.00

N=200		wcs_only	baseline_extended	behavior_simple	behavior_extended	behavior_selected	all_features
**Model**	**Measure**						
lr	**mean (SD)**	0.57 (0.07)	0.61 (0.07)	0.7 (0.06)	0.73 (0.04)	0.79 (0.03)	0.78 (0.03)
	**min/max**	0.47/0.69	0.51/0.72	0.59/0.8	0.65/0.8	0.74/0.83	0.75/0.86
	**% +0.05 test**	0.60	0.40	0.50	0.40	0.40	0.20
	**% +0.10 test**	0.30	0.10	0.10	0.10	0.00	0.10
	**% -0.05 test**	0.00	0.20	0.10	0.00	0.00	0.00
	**% -0.10 test**	0.00	0.00	0.00	0.00	0.00	0.00
svm	**mean (SD)**	0.59 (0.06)	0.63 (0.05)	0.71 (0.05)	0.74 (0.03)	0.78 (0.02)	0.76 (0.03)
	**min/max**	0.51/0.7	0.55/0.72	0.6/0.78	0.69/0.79	0.74/0.83	0.69/0.8
	**% +0.05 test**	0.60	0.60	0.60	0.60	0.30	0.30
	**% +0.10 test**	0.30	0.30	0.10	0.00	0.00	0.00
	**% -0.05 test**	0.00	0.00	0.10	0.00	0.00	0.00
	**% -0.10 test**	0.00	0.00	0.00	0.00	0.00	0.00
nb	**mean (SD)**	0.54 (0.08)	0.61 (0.06)	0.67 (0.05)	0.63 (0.03)	0.77 (0.03)	0.77 (0.03)
	**min/max**	0.38/0.66	0.55/0.72	0.58/0.76	0.57/0.67	0.71/0.81	0.72/0.82
	**% +0.05 test**	0.50	0.30	0.40	0.30	0.20	0.30
	**% +0.10 test**	0.20	0.10	0.10	0.00	0.00	0.00
	**% -0.05 test**	0.10	0.10	0.10	0.00	0.00	0.00
	**% -0.10 test**	0.10	0.00	0.00	0.00	0.00	0.00
rf	**mean (SD)**	0.6 (0.05)	0.65 (0.05)	0.74 (0.05)	0.76 (0.04)	0.82 (0.03)	0.82 (0.02)
	**min/max**	0.54/0.69	0.55/0.72	0.65/0.85	0.71/0.83	0.77/0.86	0.79/0.84
	**% +0.05 test**	0.60	0.60	0.70	0.50	0.50	0.50
	**% +0.10 test**	0.40	0.20	0.20	0.20	0.00	0.00
	**% -0.05 test**	0.00	0.00	0.00	0.00	0.00	0.00
	**% -0.10 test**	0.00	0.00	0.00	0.00	0.00	0.00
adaboost	**mean (SD)**	0.57 (0.06)	0.65 (0.05)	0.7 (0.06)	0.74 (0.05)	0.8 (0.03)	0.79 (0.02)
	**min/max**	0.49/0.68	0.55/0.73	0.58/0.79	0.68/0.87	0.75/0.84	0.74/0.83
	**% +0.05 test**	0.60	0.70	0.60	0.50	0.70	0.50
	**% +0.10 test**	0.30	0.30	0.10	0.30	0.10	0.00
	**% -0.05 test**	0.00	0.00	0.10	0.00	0.00	0.00
	**% -0.10 test**	0.00	0.00	0.00	0.00	0.00	0.00
nn	**mean (SD)**	0.57 (0.04)	0.62 (0.05)	0.7 (0.06)	0.67 (0.05)	0.77 (0.05)	0.75 (0.04)
	**min/max**	0.52/0.67	0.55/0.71	0.59/0.78	0.57/0.74	0.7/0.83	0.69/0.8
	**% +0.05 test**	0.50	0.40	0.50	0.30	0.60	0.30
	**% +0.10 test**	0.20	0.10	0.30	0.00	0.10	0.00
	**% -0.05 test**	0.00	0.00	0.10	0.10	0.00	0.00
	**% -0.10 test**	0.00	0.00	0.00	0.00	0.00	0.00

N=300		wcs_only	baseline_extended	behavior_simple	behavior_extended	behavior_selected	all_features
**Model**	**Measure**						
**lr**	**mean (SD)**	0.55 (0.07)	0.62 (0.05)	0.72 (0.05)	0.74 (0.04)	0.79 (0.02)	0.78 (0.03)
	**min/max**	0.45/0.67	0.57/0.73	0.63/0.81	0.69/0.84	0.77/0.83	0.73/0.83
	**% +0.05 test**	0.40	0.20	0.40	0.30	0.20	0.10
	**% +0.10 test**	0.20	0.10	0.10	0.10	0.00	0.00
	**% -0.05 test**	0.10	0.10	0.00	0.00	0.00	0.00
	**% -0.10 test**	0.00	0.00	0.00	0.00	0.00	0.00
**svm**	**mean (SD)**	0.57 (0.05)	0.63 (0.05)	0.72 (0.04)	0.74 (0.03)	0.78 (0.02)	0.77 (0.03)
	**min/max**	0.51/0.65	0.56/0.72	0.64/0.79	0.69/0.79	0.76/0.83	0.72/0.83
	**% +0.05 test**	0.60	0.40	0.50	0.50	0.10	0.20
	**% +0.10 test**	0.20	0.10	0.10	0.00	0.00	0.00
	**% -0.05 test**	0.00	0.00	0.00	0.00	0.00	0.00
	**% -0.10 test**	0.00	0.00	0.00	0.00	0.00	0.00
**nb**	**mean (SD)**	0.54 (0.07)	0.61 (0.06)	0.69 (0.05)	0.62 (0.05)	0.78 (0.03)	0.78 (0.03)
	**min/max**	0.42/0.66	0.52/0.7	0.62/0.77	0.55/0.71	0.74/0.81	0.73/0.82
	**% +0.05 test**	0.30	0.20	0.50	0.20	0.50	0.20
	**% +0.10 test**	0.20	0.00	0.20	0.00	0.00	0.00
	**% -0.05 test**	0.20	0.20	0.00	0.10	0.00	0.00
	**% -0.10 test**	0.00	0.00	0.00	0.00	0.00	0.00
**rf**	**mean (SD)**	0.57 (0.05)	0.64 (0.05)	0.73 (0.05)	0.76 (0.04)	0.81 (0.02)	0.82 (0.02)
	**min/max**	0.51/0.67	0.56/0.7	0.64/0.82	0.69/0.82	0.77/0.84	0.75/0.84
	**% +0.05 test**	0.40	0.40	0.60	0.50	0.30	0.40
	**% +0.10 test**	0.20	0.10	0.20	0.10	0.00	0.00
	**% -0.05 test**	0.00	0.00	0.00	0.00	0.00	0.00
	**% -0.10 test**	0.00	0.00	0.00	0.00	0.00	0.00
**adaboost**	**mean (SD)**	0.55 (0.05)	0.62 (0.04)	0.72 (0.05)	0.74 (0.04)	0.81 (0.01)	0.8 (0.03)
	**min/max**	0.49/0.67	0.54/0.69	0.64/0.8	0.68/0.84	0.78/0.83	0.71/0.82
	**% +0.05 test**	0.40	0.40	0.50	0.40	0.60	0.60
	**% +0.10 test**	0.10	0.10	0.10	0.10	0.00	0.00
	**% -0.05 test**	0.00	0.00	0.00	0.00	0.00	0.00
	**% -0.10 test**	0.00	0.00	0.00	0.00	0.00	0.00
**nn**	**mean (SD)**	0.56 (0.05)	0.63 (0.05)	0.71 (0.05)	0.7 (0.04)	0.79 (0.02)	0.77 (0.04)
	**min/max**	0.48/0.66	0.57/0.73	0.61/0.81	0.62/0.76	0.76/0.83	0.69/0.83
	**% +0.05 test**	0.50	0.30	0.40	0.40	0.20	0.20
	**% +0.10 test**	0.20	0.20	0.10	0.00	0.00	0.00
	**% -0.05 test**	0.00	0.00	0.10	0.10	0.00	0.10
	**% -0.10 test**	0.00	0.00	0.00	0.00	0.00	0.00

N=400		wcs_only	baseline_extended	behavior_simple	behavior_extended	behavior_selected	all_features
**Model**	**Measure**						
lr	**mean (SD)**	0.57 (0.05)	0.62 (0.04)	0.72 (0.03)	0.76 (0.03)	0.79 (0.02)	0.79 (0.02)
	**min/max**	0.47/0.66	0.57/0.7	0.68/0.76	0.72/0.8	0.76/0.81	0.76/0.81
	**% +0.05 test**	0.60	0.10	0.40	0.30	0.00	0.00
	**% +0.10 test**	0.20	0.00	0.00	0.00	0.00	0.00
	**% -0.05 test**	0.00	0.20	0.00	0.00	0.00	0.00
	**% -0.10 test**	0.00	0.00	0.00	0.00	0.00	0.00
svm	**mean (SD)**	0.58 (0.04)	0.63 (0.03)	0.72 (0.02)	0.75 (0.02)	0.79 (0.01)	0.78 (0.02)
	**min/max**	0.51/0.63	0.59/0.71	0.67/0.75	0.72/0.78	0.76/0.81	0.76/0.81
	**% +0.05 test**	0.50	0.20	0.30	0.30	0.30	0.10
	**% +0.10 test**	0.10	0.00	0.00	0.00	0.00	0.00
	**% -0.05 test**	0.00	0.00	0.00	0.00	0.00	0.00
	**% -0.10 test**	0.00	0.00	0.00	0.00	0.00	0.00
nb	**mean (SD)**	0.56 (0.07)	0.62 (0.04)	0.7 (0.03)	0.64 (0.04)	0.78 (0.02)	0.79 (0.01)
	**min/max**	0.38/0.64	0.54/0.68	0.65/0.74	0.59/0.71	0.75/0.81	0.77/0.82
	**% +0.05 test**	0.60	0.20	0.50	0.30	0.10	0.10
	**% +0.10 test**	0.10	0.00	0.00	0.00	0.00	0.00
	**% -0.05 test**	0.10	0.10	0.00	0.00	0.00	0.00
	**% -0.10 test**	0.10	0.00	0.00	0.00	0.00	0.00
rf	**mean (SD)**	0.58 (0.03)	0.64 (0.04)	0.73 (0.03)	0.77 (0.03)	0.82 (0.02)	0.82 (0.02)
	**min/max**	0.54/0.64	0.56/0.69	0.68/0.77	0.72/0.81	0.79/0.84	0.8/0.85
	**% +0.05 test**	0.70	0.30	0.50	0.40	0.20	0.30
	**% +0.10 test**	0.20	0.00	0.00	0.00	0.00	0.00
	**% -0.05 test**	0.00	0.10	0.00	0.00	0.00	0.00
	**% -0.10 test**	0.00	0.00	0.00	0.00	0.00	0.00
adaboost	**mean (SD)**	0.56 (0.06)	0.62 (0.04)	0.73 (0.03)	0.75 (0.03)	0.81 (0.02)	0.8 (0.02)
	**min/max**	0.43/0.65	0.54/0.66	0.67/0.76	0.68/0.8	0.78/0.84	0.75/0.82
	**% +0.05 test**	0.50	0.40	0.40	0.50	0.10	0.30
	**% +0.10 test**	0.20	0.00	0.00	0.10	0.00	0.00
	**% -0.05 test**	0.10	0.10	0.00	0.00	0.00	0.00
	**% -0.10 test**	0.00	0.00	0.00	0.00	0.00	0.00
nn	**mean (SD)**	0.56 (0.04)	0.62 (0.04)	0.71 (0.02)	0.71 (0.02)	0.8 (0.01)	0.78 (0.02)
	**min/max**	0.5/0.65	0.56/0.68	0.66/0.74	0.69/0.74	0.76/0.81	0.74/0.81
	**% +0.05 test**	0.40	0.20	0.40	0.10	0.10	0.20
	**% +0.10 test**	0.10	0.00	0.00	0.00	0.00	0.00
	**% -0.05 test**	0.00	0.00	0.00	0.00	0.00	0.00
	**% -0.10 test**	0.00	0.00	0.00	0.00	0.00	0.00

N=500		wcs_only	baseline_extended	behavior_simple	behavior_extended	behavior_selected	all_features
**Model**	**Measure**						
lr	mean (SD)	0.57 (0.04)	0.63 (0.02)	0.72 (0.02)	0.75 (0.01)	0.79 (0.02)	0.79 (0.01)
	min/max	0.49/0.66	0.61/0.66	0.69/0.74	0.73/0.77	0.76/0.81	0.77/0.81
	% +0.05 test	0.50	0.00	0.10	0.00	0.00	0.00
	% +0.10 test	0.10	0.00	0.00	0.00	0.00	0.00
	% -0.05 test	0.00	0.00	0.00	0.00	0.00	0.00
	% -0.10 test	0.00	0.00	0.00	0.00	0.00	0.00
svm	mean (SD)	0.57 (0.03)	0.64 (0.02)	0.71 (0.02)	0.74 (0.02)	0.78 (0.02)	0.79 (0.02)
	min/max	0.52/0.65	0.6/0.68	0.67/0.73	0.71/0.77	0.75/0.81	0.76/0.82
	% +0.05 test	0.60	0.20	0.00	0.20	0.20	0.20
	% +0.10 test	0.10	0.00	0.00	0.00	0.00	0.00
	% -0.05 test	0.00	0.00	0.00	0.00	0.00	0.00
	% -0.10 test	0.00	0.00	0.00	0.00	0.00	0.00
nb	mean (SD)	0.57 (0.05)	0.63 (0.02)	0.69 (0.02)	0.65 (0.04)	0.78 (0.02)	0.79 (0.01)
	min/max	0.47/0.65	0.59/0.68	0.65/0.73	0.59/0.74	0.76/0.8	0.76/0.81
	% +0.05 test	0.50	0.10	0.20	0.20	0.20	0.00
	% +0.10 test	0.10	0.00	0.00	0.10	0.00	0.00
	% -0.05 test	0.00	0.00	0.00	0.00	0.00	0.00
	% -0.10 test	0.00	0.00	0.00	0.00	0.00	0.00
rf	mean (SD)	0.58 (0.04)	0.65 (0.02)	0.72 (0.02)	0.76 (0.02)	0.81 (0.01)	0.82 (0.01)
	min/max	0.52/0.65	0.63/0.68	0.69/0.76	0.74/0.8	0.79/0.83	0.79/0.83
	% +0.05 test	0.50	0.30	0.20	0.30	0.10	0.00
	% +0.10 test	0.10	0.00	0.00	0.00	0.00	0.00
	% -0.05 test	0.00	0.00	0.00	0.00	0.00	0.00
	% -0.10 test	0.00	0.00	0.00	0.00	0.00	0.00
adaboost	mean (SD)	0.56 (0.04)	0.63 (0.03)	0.72 (0.02)	0.74 (0.02)	0.81 (0.02)	0.8 (0.02)
	min/max	0.49/0.64	0.58/0.67	0.69/0.76	0.72/0.78	0.78/0.83	0.77/0.83
	% +0.05 test	0.60	0.10	0.10	0.20	0.20	0.20
	% +0.10 test	0.10	0.00	0.00	0.00	0.00	0.00
	% -0.05 test	0.00	0.00	0.00	0.00	0.00	0.00
	% -0.10 test	0.00	0.00	0.00	0.00	0.00	0.00
nn	mean (SD)	0.56 (0.04)	0.63 (0.03)	0.7 (0.02)	0.71 (0.01)	0.79 (0.02)	0.78 (0.02)
	min/max	0.53/0.66	0.59/0.67	0.67/0.72	0.68/0.73	0.76/0.81	0.75/0.81
	% +0.05 test	0.40	0.20	0.00	0.10	0.00	0.20
	% +0.10 test	0.10	0.00	0.00	0.00	0.00	0.00
	% -0.05 test	0.00	0.00	0.00	0.00	0.00	0.00
	% -0.10 test	0.00	0.00	0.00	0.00	0.00	0.00

Supplementary Note 1 – Study design and intervention description

As part of the EU-funded ICare project aiming at integrating technology into mental health care delivery in Europe, the everyBody dissemination study combined evidence-based eating disorder (ED) prevention and health promotion programs[1–6]. Participants (N=3,654) were women recruited from the general population without fulfilling the criteria for a full-syndrome binge eating disorder, bulimia nervosa, anorexia nervosa or underweight. The study was a nonrandomized, parallel-group interventional design where intervention content was matched with risk and/or symptom levels. Each intervention arm featured a distinct version of the program, customized to varying risk profiles in the target population.

Prospective study participants were screened for eligibility in an online assessment. They were excluded if the screening showed a high risk for a full-syndrome ED (AN, BN, or BED), if they were currently or had been in treatment for an ED in the past six months, if they reported a Body Mass Index (BMI) of less than 18.5, or if they were younger than 18 years. All eligible participants were then allocated to one of the five study arms corresponding to the screening information (presence of subthreshold binge eating/purging, BMI, extent of weight and shape concerns). The full allocation rationale is shown in Supplementary Figure 1.

Time between screening and baseline assessment varied between 0-2 weeks. Following inclusion, participants completed baseline assessments and received access to the intervention they had been assigned to. Further assessments took place at mid-intervention, post-intervention, 6-month follow-up, and at 12-month follow-up. Mid-intervention assessment was administered 4 weeks after baseline (except Basic). Post-intervention assessments were administered 4, 8, 10 or 12 weeks after baseline, depending on duration of the intervention in each study arm (see Supplementary Table 5).

In total, 4,886 women were screened, 3,787 provided data for the baseline assessment and 3,654 were allocated to one of the five study arms. Of those, 452 were allocated to the study arm Basic, 397 to Original, 1,386 to Plus, 80 to AN, and 1,339 to study arm Fit.

Interventions

The interventions consisted of weekly sessions spanning 4 to 12 weeks, but all rooted in cognitive-behavioral principles. They encompassed psychoeducational components, activities aimed at fostering positive body image and healthy eating habits, and, where applicable, strategies to mitigate eating disorder symptoms.

Study arm Basic (4 weeks) was provided as universal prevention for women who showed no elevated risk for an ED in the screening. In study arm Original (8 weeks), women with elevated weight and shape concerns were offered content to improve body image and balanced eating and exercise habits, as well as access to moderated discussion boards with other study participants from the same study arm for support. Study arm Fit (12 weeks) was tailored for women with an elevated BMI (>25) and was supplemented with discussion boards as well. Study arm Plus (8 weeks) was aimed at women with elevated weight and shape concerns, occasional binge eating and/or compensatory behaviors. Study arm AN included women with elevated weight and shape concerns and a lower BMI (<21).

Study arms Original, Plus, AN and Fit were supplemented with online diaries to track eating habits, exercise behavior, as well as thoughts and feelings related to body image and disordered eating on a daily or weekly basis.

Both the Plus and AN arm were guided, i.e., in addition to moderated discussion groups for peer support, these interventions also offered weekly, personalized feedback messages by coaches to elaborate the intervention content. Feedback was given based on participants' free text answers to questions and exercises in the sessions, the entries in diaries, messages in group discussions, and their replies to previous messages from coaches.

Participation was anonymous and participants were encouraged to complete one session (20 to 60 minutes) per week. To prevent program completion ahead of the intended schedule, there was a one-week break between releasing sessions, however, there was no upper time limit for completing the intervention. If a session was not completed within a week from its release, a reminder email was sent.

Supplementary References 1

1. Nacke, B. *et al.* everyBody–Tailored online health promotion and eating disorder prevention for women: Study protocol of a dissemination trial. *Internet Interv.* **16**, 20–25 (2019).
2. Jacobi, C. *et al.* Indicated Web-Based Prevention for Women With Anorexia Nervosa Symptoms: Randomized Controlled Efficacy Trial. *J. Med. Internet Res.* **24**, e35947 (2022).
3. Jacobi, C., Völker, U., Trockel, M. T. & Taylor, C. B. Effects of an Internet-based intervention for subthreshold eating disorders: A randomized controlled trial. *Behav. Res. Ther.* **50**, 93–99 (2012).
4. Jacobi, C. *et al.* Maintenance of internet-based prevention: A randomized controlled trial. *Int. J. Eat. Disord.* **40**, 114–119 (2007).
5. Beintner, I., Emmerich, O. L. M., Vollert, B., Taylor, C. B. & Jacobi, C. Promoting positive body image and intuitive eating in women with overweight and obesity via an online intervention: Results from a pilot feasibility study. *Eat. Behav.* **34**, 101307 (2019).
6. Heier, H. everyBody Basic – Ein Online-Kurzprogramm zur Gesundheitsförderung bei Frauen ohne Essstörungsrisiko - Ergebnisse einer Pilotstudie.

Supplementary Figure 1: Allocation process based on symptom intensity

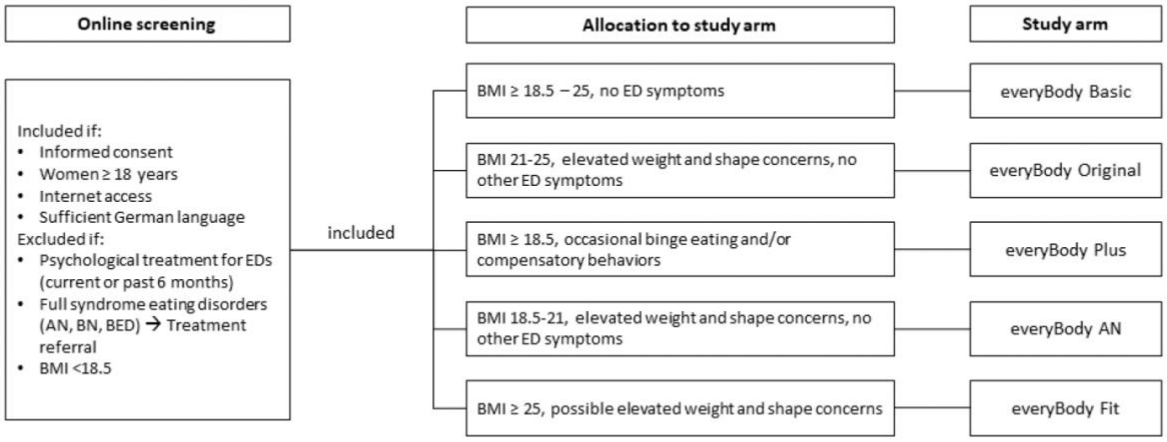

Flow chart of participants from online screening via the inclusion and study arm allocation measures to the respective intervention.

Supplementary Table 5: Characteristics of five study arms

	everyBody study arm				
	Basic	Original	Plus	AN	Fit
ED symptoms	None	Elevated weight and shape concerns, no other ED symptoms	Elevated weight and shape concerns, occasional binge eating and/or compensatory behaviors	Elevated weight and shape concerns, no other ED symptoms	Possible elevated weight and shape concerns, no other ED symptoms
BMI	18.5-25	21-25	≥ 18.5	18.5-21	> 25
Duration (weeks)	4	8	8	10	12
Aims	Promoting balanced eating and exercise habits, body image	Improving body image, promoting balanced eating and exercise habits	Improving body image, establishing balanced eating and exercise habits, improving self-esteem, reducing compensatory behaviors	Improving body image, establishing balanced eating and exercise habits, improving self-esteem, reducing dietary restraint	Healthy weight regulation, promoting balanced eating and exercise habits, improving self-esteem and body satisfaction
Moderated discussion groups	No	Yes	Yes	Yes	Yes
Individualized weekly feedback	No	No	Yes	Yes	No
Accompanying diaries	No	Yes	Yes	Yes	Yes

Supplementary Note 2 – Feature Engineering

Baseline Questionnaire Data

Using only questionnaire data provided before the intervention (baseline data) has proven to have no or very limited prediction potential on its own[1–4]. Therefore, it will be used to investigate the performance with assumed low information-to-noise ratio dependent on data set sizes.

It has widely been argued that questionnaires should be kept to a minimum due to data minimalism requirements, time needed and adherence. Further, features are commonly observed to have high multicollinearity[5–7], resulting in decreasing marginal benefits per features. To consider this aspect, the investigation of questionnaire data will be split into two parts: Firstly, the *simple questionnaire data* only comprises the intervention information (i.e., intervention arm, duration in weeks, content information, start year) and the symptom data in form of the Weight Concerns Scale (WCS)[8] at screening and before intervention start. Secondly, *extended questionnaire data* further includes a battery of questions covering different aspects beyond the WCS. Concerning ED pathology, the sum of Eating Disorder Examination-Questionnaire (EDE-Q) global score covers restrained eating behaviors, eating concerns, and weight and shape concerns[9,10]. The times users experienced loss of control eating in the last three months before screening is also recorded. Via the Intuitive Eating Scale (IES)[11,12], intuitive eating is assessed. Further, body mass index (one missing value) is reported at both screening and right before the intervention starts. To cover users weight loss history, they are asked how much weight, if any, they have lost at most in the past year and in what time frame they have lost it. In addition, the summed portions of vegetables and fruits consumed in the last week is entered. Beyond ED questionnaires, the sum of depression score (PHQ9[13]) and general anxiety score (GAD7[14]) are recorded as well as whether the user has ever been diagnosed with a psychiatry disorder and received therapy for it. Further, the score of the Alcohol Use Disorders Identification Test (AUDIT)[15] (1% missing values) and Rosenberg Self-Esteem Scale (RSE)[16] are included. The Short Self-Regulation Questionnaire (SSRQ)[17] and the big-5 personality score (BFI-10[18]) were optional for most users, which is why they account for most missing values at around 14% each.

To retain the information of missing optional questionnaires, additional variables indicating missing values are added before imputation. In terms of socio-demographic data, age in years at screening, level of education across six categories, binary relationship status, city size across seven ordinal variables and occupational status across seven categories are included. In addition, elements of the intervention and users' expectation regarding the intervention are added.

User Intervention Behavior Data

A wide variety of intervention user behavioral data can be extracted from the user's automatically recorded interaction with the DMHI platform. Previous research has shown to have high predictive power regarding dropout predictions[1,2,19–21]. However, this ease of automatic collection can quickly cause a dataset to grow into hundreds of variables and previous research suggests that fewer, hand-crafted and selected features are most promising[19]. One paper even argues that a single variable type suffices to accurately predict

dropout[1]. Therefore, a group of simple, extended, and hand-crafted features are investigated in this paper. To leave sufficient time to intervene against dropout, the predictions are made after the first week of the intervention. Therefore, all data generated after the first seven days of intervention start of the respective user is disregarded before feature engineering.

For the *simple behavior data,* we follow related work for generalizable features in DMHIs and count the users' number of logins per day for the first week of the intervention[1,22].

For the extended and hand-crafted user behavior*,* the log files, and patient submissions from the first week of the intervention are turned into more features[19]. This includes if and on what day a session was completed, seconds spent on the platform, and the count of logins. Text meta data from asynchronous communication within the intervention has previously been shown to be highly predictive for dropout[2]. Therefore, for the interventions that had access to the group or coach message function, the total sum of characters is counted as well as the number of messages sent. From the diaries, the number of submissions and length of text in the diaries are calculated. Similarly, for the exercises and questions within the intervention, the number of closed-question answers are used as well as the number of open-text questions. For the latter, further the number of characters is recorded. If no entry is found for any of the users, it is assumed that no such activity occurred.

For the *selected user behavior,* these features are aggregated per week to lower sparsity, multicollinearity, and the number of features, resulting in seven features. The features selected are based on the related research discussed above and the authors' previous work[2,21,23]. Further, beyond the availability of different open-text forms, these features types can all reasonably be expected to be available across any digital intervention[2,19].

For the *extended user behavior,* the data is separately aggregated per day for the first week and include additional less known or theoretically less informative features. These include the sum of seconds spent on the intervention during the beginning (Mo-Tu), middle (Wed-Thurs) or end (Fr-Sun) of the week, and morning (4am-12pm), daytime (12-6pm) or evening (6pm-4am). Further, as intervention specific features, the mean, minimum and maximum numbers of answers given to the sixteen most commonly answered closed-answer questions are added. These primarily record eating related habits such as number of healthy or fast-food meals. This totals to the largest group at features at 129. To consider possible interaction effects between the two types of features that have been proposed to add predictive value[2], the simple and selected behavior and extended questionnaire data are added together in the *mixed run* with 71 features.

Variables not used

The original study gathered further questionnaire data, however, many of them had no assumed information value for dropout and many missing values (e.g., frequency of vomiting if user had already said "no" in previous question), low variance (e.g., pregnancy), high cardinality (e.g., dummies for the 16 German states) or were highly correlated to other included values (e.g., single score symptom questions). The goal of this study, however, was not to investigate which of the baseline questions had the most predictive power but rather to investigate one feature group with few (D=2) and one with a larger but not unobtainable amount (D=51). Therefore, only the above described were chosen to be included in discussion between the authors KZ, BN and DG. Selection criteria was primarily previous evidence for their predictive power regarding dropout, having an information value beyond most answers being missing or the same and limited overlap with other, already included features. As such,

a variety of symptom questions regarding exercise, taking laxatives, vomiting as well as socio-demographic data regarding state, year of birth and minimum BMI in previous year were not included.

Supplementary References 2

1. Bricker, J., Miao, Z., Mull, K., Santiago-Torres, M. & Vock, D. M. Can a Single Variable Predict Early Dropout From Digital Health Interventions? Comparison of Predictive Models From Two Large Randomized Trials. *J. Med. Internet Res.* **25**, e43629 (2023).
2. Zantvoort, K., Scharfenberger, J., Boß, L., Lehr, D. & Funk, B. Finding the Best Match — a Case Study on the (Text-)Feature and Model Choice in Digital Mental Health Interventions. *J. Healthc. Inform. Res.* **7**, 447–479 (2023).
3. Linardon, J., Fuller-Tyszkiewicz, M., Shatte, A. & Greenwood, C. J. An exploratory application of machine learning methods to optimize prediction of responsiveness to digital interventions for eating disorder symptoms. *Int. J. Eat. Disord.* **55**, 845–850 (2022).
4. Günther, F., Yau, C., Elison-Davies, S. & Wong, D. On the Difficulty of Predicting Engagement with Digital Health for Substance Use. *Stud. Health Technol. Inform.* **302**, 967–971 (2023).
5. Patel, V. *et al.* Detecting common mental disorders in primary care in India: A comparison of five screening questionnaires. *Psychol. Med.* **38**, 221–8 (2008).
6. Tomitaka, S. & Furukawa, T. A. The GAD-7 and the PHQ-8 exhibit the same mathematical pattern of item responses in the general population: analysis of data from the National Health Interview Survey. *BMC Psychol.* **9**, 149 (2021).
7. Sander, J., Moessner, M. & Bauer, S. Depression, Anxiety and Eating Disorder-Related Impairment: Moderators in Female Adolescents and Young Adults. *Int. J. Environ. Res. Public. Health* **18**, 2779 (2021).
8. Killen, J. D. *et al.* Pursuit of thinness and onset of eating disorder symptoms in a community sample of adolescent girls: a three-year prospective analysis. *Int. J. Eat. Disord.* **16**, (1994).
9. Fairburn, C. G. & Beglin, S. J. Eating Disorder Examination Questionnaire. in *Cognitive Behavior Therapy and Eating Disorders* (Guildford Press, New York, NY, USA, 2008).
10. Hilbert, A. & Tuschen-Caffier, B. *Eating Disorder Examination-Questionnaire*. (dgvt-Verlag, Tübingen, Germany, 2016).
11. Herbert, B. M., Blechert, J., Hautzinger, M., Matthias, E. & Herbert, C. Intuitive eating is associated with interoceptive sensitivity. Effects on body mass index. *Appetite* **70**, 22–30 (2013).
12. Tylka, T. L. Development and psychometric evaluation of a measure of intuitive eating. *J. Couns. Psychol.* **53**, 226–240 (2006).
13. Kroenke, K., Spitzer, R. L. & Williams, J. B. W. The PHQ-9. *J. Gen. Intern. Med.* **16**, 606–613 (2001).
14. Spitzer, R. L., Kroenke, K., Williams, J. B. W. & Löwe, B. A brief measure for assessing generalized anxiety disorder: the GAD-7. *Arch. Intern. Med.* **166**, 1092–1097 (2006).
15. Saunders, J. B., Aasland, O. G., Babor, T. F., De La Fuente, J. R. & Grant, M. Development of the Alcohol Use Disorders Identification Test (AUDIT): WHO Collaborative Project on Early Detection of Persons with Harmful Alcohol Consumption-II. *Addiction* **88**, 791–804 (1993).
16. Rosenberg, M. Society and the Adolescent Self-Image. in *Society and the Adolescent Self-Image* (Princeton University Press, 1979). doi:10.1515/9781400876136.
17. Carey, K. B., Neal, D. J. & Collins, S. E. A psychometric analysis of the self-regulation questionnaire. *Addict. Behav.* **29**, 253–260 (2004).
18. Rammstedt, B., Kemper, C., Klein, M., Beierlein, C. & Kovaleva, A. *Eine Kurze Skala Zur Messung Der Fünf Dimensionen Der Persönlichkeit: Big-Five-Inventory-10 (BFI-10)*. (2012).

19. Bremer, V., Chow, P. I., Funk, B., Thorndike, F. P. & Ritterband, L. M. Developing a Process for the Analysis of User Journeys and the Prediction of Dropout in Digital Health Interventions: Machine Learning Approach. *J. Med. Internet Res.* **22**, (2020).
20. Pedersen, D. H., Mansourvar, M., Sortsø, C. & Schmidt, T. Predicting Dropouts From an Electronic Health Platform for Lifestyle Interventions: Analysis of Methods and Predictors. *J. Med. Internet Res.* **21**, (2019).
21. Zantvoort, K., Isacsson, N. H., Funk, B. & Kaldo, V. Data set size vs homogeneity – A Machine Learning study on pooling intervention data in E-Mental Health dropout predictions. *SAGE Digital Health* (2024).
22. Cote-Allard, U., Pham, M. H., Schultz, A. K., Nordgreen, T. & Torresen, J. Adherence Forecasting for Guided Internet-Delivered Cognitive Behavioral Therapy: A Minimally Data-Sensitive Approach. *IEEE J. Biomed. Health Inform.* 1–12 (2022) doi:10.1109/JBHI.2022.3204737.
23. Hornstein, S., Forman-Hoffman, V., Nazander, A., Ranta, K. & Hilbert, K. Predicting therapy outcome in a digital mental health intervention for depression and anxiety: A machine learning approach. *Digit. Health* **7**, 205520762110606 (2021).
